# Engaging Parents in Technology-Assisted Interventions for Childhood Adversity: Systematic Review

**DOI:** 10.2196/43994

**Published:** 2024-01-19

**Authors:** Grace Aldridge, Alessandra Tomaselli, Clare Nowell, Andrea Reupert, Anthony Jorm, Marie Bee Hui Yap

**Affiliations:** 1 Turner Institute for Brain and Mental Health, School of Psychological Sciences Monash University Clayton Australia; 2 School of Educational Psychology and Counselling Monash University Clayton Australia; 3 Melbourne School of Population and Global Health University of Melbourne Melbourne Australia

**Keywords:** systematic review, intervention, digital technology, parenting, children, technology, parenting program, engagement, support, adverse childhood experiences

## Abstract

**Background:**

Youth mental health problems are a major public health concern and are strongly associated with adverse childhood experiences (ACEs). Technology-assisted parenting programs can intervene with ACEs that are within a parent’s capacity to modify. However, engagement with such programs is suboptimal.

**Objective:**

This review aims to describe and appraise the efficacy of strategies used to engage parents in technology-assisted parenting programs targeting ACEs on the behavioral and subjective outcomes of engagement.

**Methods:**

Using PRISMA (Preferred Reporting Items for Systematic Reviews and Meta-Analyses) reporting guidelines, we conducted a systematic review of peer-reviewed papers that described the use of at least 1 engagement strategy in a technology-assisted parenting program targeting ACEs that are within a parent’s capacity to modify. A total of 8 interdisciplinary bibliographic databases (CENTRAL, CINAHL, Embase, OVID MEDLINE, OVID PsycINFO, Scopus, ACM, and IEEE Xplore) and gray literature were searched. The use of engagement strategies and measures was narratively synthesized. Associations between specific engagement strategies and engagement outcomes were quantitatively synthesized using the Stouffer method of combining *P* values.

**Results:**

We identified 13,973 articles for screening. Of these, 156 (1.12%) articles were eligible for inclusion, and 29 (18.2%) of the 156 were associated with another article; thus, 127 studies were analyzed. Preliminary evidence for a reliable association between 5 engagement strategies (involving parents in a program’s design, delivering a program on the web compared to face-to-face, use of personalization or tailoring features, user control features, and provision of practical support) and greater engagement was found. Three engagement strategies (professional support features, use of videos, and behavior change techniques) were not found to have a reliable association with engagement outcomes.

**Conclusions:**

This review provides a comprehensive assessment and description of the use of engagement strategies and engagement measures in technology-assisted parenting programs targeting parenting-related ACEs and extends the current evidence with preliminary quantitative findings. Heterogeneous definition and measurement of engagement and insufficient engagement outcome data were caveats to this synthesis. Future research could use integrated definitions and measures of engagement to support robust systematic evaluations of engagement in this context.

**Trial Registration:**

PROSPERO CRD42020209819; https://www.crd.york.ac.uk/prospero/display_record.php?RecordID=209819

## Introduction

### Parenting Programs to Prevent or Reduce Depression and Anxiety Disorders in Young People

Depression and anxiety disorders are major sources of the global disease burden in children and young people [1,2]. Parenting factors are known to influence the risk of developing these disorders in children and adolescents (ie, offspring aged 0-18 years, henceforth referred to as “young people”) [3,4]. Unlike other known systemic or biological factors (eg, poverty or family history of psychopathology), parenting factors are within a parent’s capacity to intervene. Parenting programs capitalize on the central role that parents and caregivers play in a young person’s development by improving the parenting skills involved in supporting their young person’s outcomes. There is good evidence to support the efficacy of parenting programs in improving young people’s mental health outcomes [5] in between-group comparisons over time when compared with a control condition (no treatment) [6] and when compared with a range of control conditions such as usual care or attention controls [7]. However, patterns of poor engagement (such as low rates of enrollment and program completion) are common in studies of face-to-face parenting programs, which limit the potential benefits of these programs at the family and population level [8,9]. Common barriers include time constraints, conflicting schedules, perceived stigma, and stress from involvement in parenting programs [10,11].

### Parenting Programs for Parents of Young People With Adverse Childhood Experiences

Barriers to engaging in parenting programs are especially prevalent in families who experience adversity, marginalization, and stress owing to socioeconomic pressure [12,13]. Parental stress is associated with maladaptive parenting behaviors (defined as parenting behaviors characterized by high hostility and low warmth [14], henceforth referred to as “maladaptive parenting”) [15]. Recent evidence suggests that maladaptive parenting is as predictive of mental disorders and suicidality in young people as more commonly known family-level adverse childhood experiences (ACEs; such as child maltreatment and interparental conflict) [16]. Therefore, there is a clear potential for parenting programs to intervene with these family-level ACEs and to reduce or prevent the risk of mental disorders in young people. Further, benefits from engaging with such programs may potentially buffer against the stress experienced because of other systemic ACEs. However, a better understanding of strategies to enhance engagement with programs targeting family-level ACEs is needed, given that target families are likely to experience greater barriers to engagement.

### Technology-Assisted Parenting Programs

Technology has the potential to minimize or overcome common barriers associated with engaging in face-to-face parenting programs. For example, technology can offer a user flexibility and choice regarding how and when they access a parenting program, as well as increased privacy. Functions such as automated reminders and content tailoring may also enhance the relevance and relationship between the program and its user [17]. The delivery and reach of existing services for parents can also be enhanced with technology, as it can carry out progress monitoring and content updates in a time-efficient manner and with fewer human resources. The potential benefits of technology-assisted parenting programs have therefore been widely explored over the past 2 decades [18], especially in recent years owing to the effects of the COVID-19 pandemic on families’ ability to access face-to-face services [19]. There is a growing body of evidence supporting the efficacy of technology-assisted parenting programs in improving parenting outcomes (including maladaptive parenting), reducing their young person’s internalizing problems [20,21] and externalizing problems [22-24], and promoting their physical and mental health [25]. Importantly, the efficacy of these programs has also been found for parenting outcomes and child problem behaviors for families experiencing socioeconomic disadvantage [26]. However, program effect sizes are often found to be small, warranting further exploration of how these effects might be enhanced.

### Parental Engagement in Technology-Assisted Parenting Programs

Engagement in a program is a key mechanism for improving target behavioral outcomes [27]. In the context of parenting programs, engagement has specifically been conceptualized as 3 discrete behavioral components: *initial* engagement, measured by both intended and actual enrollment in a program; *ongoing* engagement, as indicated by measures of attendance or program completion; and *quality* of engagement, as shown through measures of active participation (such as completing specific program components either within or beyond the program itself) [28]. It has been suggested that the *quality* of engagement is most closely related to program outcomes and, hence, is suggested to be a key mechanism for positive parenting change [29].

Several systematic reviews evaluating the effects of technology-assisted parenting programs on target parents and young people’s outcomes have also explored the use of specific engagement strategies or program features. For instance, Florean et al [24] found that parenting programs to reduce elevated or diagnosed behavior problems in young people that are delivered via videoconferencing yielded comparable effects on young people’s outcomes with the programs delivered face-to-face. They also found that the effects on both young people and parenting outcomes were comparable between conditions that provided specialized support (ie, understanding and applying program content) and conditions that provided technical support (ie, using and navigating the program) [24]. Similarly, Spencer et al [21] found that web-based parenting programs with additional clinical support (ie, access to a specialist or therapist in addition to the program) did not significantly influence the strength of program effects on a range of parenting and young people’s outcomes compared with programs without clinical support. Corralejo and Domenech Rodrigues [23] noted that although more than half of 31 included studies on behavioral parent training programs included coaching components, no study compared program effects between conditions with and without coaching. Thongseiratch et al [30] used qualitative comparative analysis to identify specific program components associated with stronger program effects on child behavioral problems. Their analysis revealed that sending reminders to parents was the only effective feature, whereas additional phone calls were associated with weaker program effects. Notably, these reviews did not consider or explore the effect of these features on program engagement outcomes despite endorsing the utility of technology in improving accessibility.

Hansen et al [20] attempted to explore the relationship between program engagement strategies and engagement outcomes in a systematic review of randomized controlled trials (RCTs) of technology-assisted parenting programs. They found that recruitment and tailoring strategies were linked to higher postintervention *study* retention rates, but limited and inconsistent reporting of *program* adherence outcomes across studies precluded drawing conclusions about the effects of such strategies on parents’ engagement with the program itself [20]. In studies on face-to-face parenting programs, reporting of data on program adherence outcomes has also been limited [8,27] or absent [28]. As program adherence measures *quality* of parental engagement with a parenting program, and *quality* of engagement is a key mechanism for changes in parenting outcomes [29], greater focus on measuring and evaluating *quality* of engagement is warranted.

### Conceptualizing Engagement in Technology-Assisted Parenting Programs

Studies of engagement with technology-assisted parenting programs are largely undertaken within the behavioral science discipline, hence engagement is typically conceptualized and measured in behavioral terms (eg, use of the program as a whole or per components, known as “dose” or “adherence,” respectively) [31]. However, the design and delivery of technology-assisted programs is informed by multiple disciplines, meaning that there are often differences in theory that result in highly varied conceptualizations of engagement in the literature [32]. For example, in the computer science and human-computer interaction disciplines, engagement is conceptualized in both behavioral *and* subjective terms, with subjective terms referring to experiences that emerge in the momentary interaction with the program [31]. Thus, conclusions drawn by behavioral science evaluations of engagement with technology-assisted programs may be both deepened and advanced by using behavioral *and* subjective measures of engagement.

To reduce the fragmentation of research objectives and findings between disciplines, Perski et al [31] proposed an integrated definition and conceptual framework of engagement, which maps both evidence-based and hypothesized influences of engagement based on available interdisciplinary literature. This comprehensive conceptualization of engagement may advance the behavioral science understanding of enhancing engagement. For instance, evidence on the influences of engagement may assist program developers to consider and design program features that enhance these influences and broaden the range of a program’s engagement strategies. In addition to program features, this framework includes *context* in its conceptualization of engagement [31]. Prior research has suggested that a program’s level of prevention (ie, universal, selective, or indicated) is an important contextual factor to consider in enhancing engagement, as the intensity of program involvement at each level (and consequently, the effort typically required of program users at each level) may require different strategies to engage users [33].

### This Systematic Review

Intervening with family-level ACEs represents an important focus in efforts to reduce or prevent the risk of mental disorders among young people. Engagement represents a key mechanism by which programs yield desired improvements in target parenting outcomes [29], and technology can potentially improve engagement by overcoming common barriers. However, to date, no study has systematically synthesized engagement strategies and outcomes in technology-assisted parenting programs. Further, the behavioral science understanding of engagement strategies and measures that can be used in the design and delivery of technology-assisted programs has likely not been sufficiently conceptualized to account for the full experience of engagement [31]. The primary aim of this review is to address this knowledge gap by (1) describing the range of engagement strategies reported in the design and delivery phases of technology-assisted parenting programs targeting an ACE, (2) exploring any patterns in the use of engagement strategies based on the program’s level of prevention, and (3) describing the range of behavioral and subjective engagement measures used in studies of technology-assisted parenting programs targeting a family-level ACE. The secondary aim of this review is to synthesize, where possible, the effects of specific engagement strategies on engagement outcomes and the associated target ACE outcomes.

## Methods

The search strategy, inclusion criteria, primary and secondary outcomes, and proposed data synthesis methods were prespecified, registered, and published on the PROSPERO database (CRD42020209819).

### Information Sources

Following consultation with academic librarians within the School of Psychological Sciences and Faculty of Information Technology at Monash University, the following 8 electronic bibliographic databases were searched: CENTRAL, CINAHL, Embase, OVID MEDLINE, OVID PsycINFO, Scopus, ACM, and IEEE Xplore.

An effective combination of search terms was designed by the first author (GA) according to the PRISMA (Preferred Reporting Items for Systematic Reviews and Meta-Analyses) statement [34] and in consultation with an academic librarian. The terms were identified based on relevant prior research and keyword search. Syntax was specific to each database. Keywords and terms used in IT databases were less specific than those used in health databases to maximize the yield of potentially eligible studies. Search terms for all databases included multiple terms for target ACE concepts (maladaptive parenting, child maltreatment, and interparental conflict), parents, interventions and programs, and technology (Multimedia Appendix 1 provides a full list).

### Search Strategy

**Figure 1:**
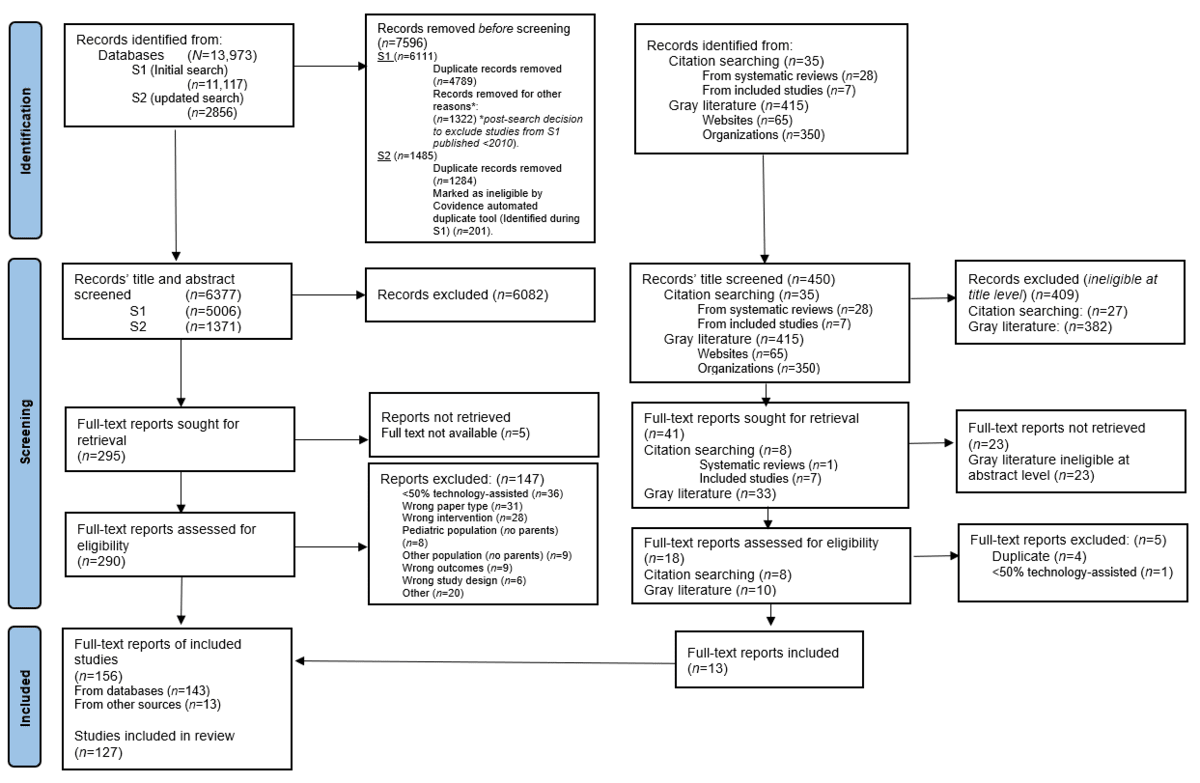
PRISMA (Preferred Reporting Items for Systematic Reviews and Meta-Analyses) flowchart of the studies selection process.

The first search was conducted on November 26, 2020. No language or date filter was applied to ensure that a diverse range of studies was retrieved. Abstracts from studies published in languages other than English were entered into Google Translate during screening to ascertain whether they were eligible for a full-text review. No such studies were eligible, hence further translational resources were not required. Although no date filter was applied during the search, a recency criterion was later applied following consultation with other authors and academics, leading to the exclusion of studies published before 2010. This decision was made given that technology-assisted interventions are more susceptible to changes over time owing to rapid advances in technology, hence research regarding these interventions’ engagement capacity may be quickly outdated. This approach is consistent with the decisions made in previous reviews [26,35]. Reference lists of relevant systematic reviews identified in the search were manually searched to identify additional studies that were either overlooked or missed in the initial electronic database search. A gray literature search was also conducted to fully exploit available data, defined as targeted website browsing of relevant authorities and organizations and search engine searching (Multimedia Appendix 1 provides documented results). The flow of studies identified, screened, and excluded based on recency criterion can be found in the PRISMA diagram (Figure 1).

Following the initial search, it was decided among the review team to exclude 2 target ACEs (parental physical and mental illness and bullying) on the basis that they are not specifically parenting behaviors and hence less within a parent’s capacity to modify. Thus, the studies returned from these 2 searches did not undergo further screening following retrieval. The PROSPERO record was updated to reflect this decision, along with the decision to apply a recency criterion and an elaboration on the proposed data synthesis methods. To ensure that the latest data were included in the review, an updated database search was conducted by the first author (GA) on November 30, 2021, to include studies published between December 2020 and November 2021. The reference lists of relevant systematic reviews identified in this search were manually searched to identify any additional studies that had been missed in the electronic database search.

### Eligibility Criteria

Studies were included in the review if the study met the following criteria: (1) published in a peer-reviewed journal or publication (except for gray literature); (2) reported on a delivered intervention targeting ≥1 of the 3 predefined, modifiable ACEs (maladaptive parenting, child maltreatment, or interparental conflict); (3) >50% of the intervention was directed at parents or caregivers of young people aged 0-18 years; (4) >50% of the intervention was delivered through technology-assisted methods or platforms; (5) described at least 1 strategy used to engage parents in the design or the delivery of the intervention; and (6) published during or after 2010. It is understandably typical for interventions targeting ACEs to self-describe from a strengths-based perspective, hence the study’s background and intervention outcome measures were checked to verify whether the intervention was intended to target ACEs if otherwise unclear. Included studies that met the following additional criteria were included for answering this review’s secondary aim: (1) a comparison group whose engagement was measured and compared with the experimental group (eg, treatment as usual, active control, attention control) and (2) between-group statistical analyses were conducted, with statistical significance reported. The peer-review criterion was also required for any gray literature that was otherwise eligible for inclusion in answering the secondary aim. Multimedia Appendix 2 provides further detail with regard to the inclusion and exclusion criteria.

### Selection Process

Following the removal of duplicate references (using both human and automated tools via Covidence v2815 systematic review software), 2 authors (GA and CN) independently double screened the titles and abstracts of studies that were identified through the first search. One author (GA) screened the titles and abstracts identified through the second database search.

### Data Collection Process

Full-text articles of potentially eligible studies identified through both searches were assessed independently by 3 authors (GA, AT, and CN), with each study assessed by 2 authors. Each author worked blinded and independently until all studies were assessed, both at the level of title and abstract and full-text screening. Discrepancies in eligibility assessment were resolved through discussion with MY. All reasons for exclusion are documented in the PRISMA diagram (Figure 1).

Extraction of key study characteristics and outcomes was completed using a standardized form on Excel (Microsoft Corporation), which was prepiloted on 10 studies (ranging in study design) by the first author (GA). All extractions were independently completed by 2 of 3 researchers (GA, AT, and CN), with the first author (GA) extracting from all studies.

### Data Items: Study Characteristics and Coding of Predictors and Outcomes

The extracted study characteristics included the country of sample population; sample size; demographics (age, sex, and socioeconomic position) of the target parent or caregiver and young person; mode and function of technology-assisted intervention components; level of intervention (eg, universal, selective, or indicated prevention); intervention design frameworks; proportion and intended duration of technology-assisted component; and type of ACE outcome targeted (ie, maladaptive parenting, child maltreatment, or interparental conflict).

The extracted predictors included reported engagement strategies or program features designed to engage parents, strategies, or features not specifically reported but identified according to definitions from prior research [17,31]. Engagement strategies (predictors) were primarily identified as attributes of a technology-assisted parenting program that prior research has identified as having an evidence-based or hypothesized influence on engagement with the program [17,31,36]. An overview of these attributes and definitions is provided in Table 1.

**Table 1:** Overview of engagement strategy codes.

Strategies and attributes			Definition
**Program design strategies**			
	Consultation	Processes or activities that seek to obtain verbal or conceptual input about the design or delivery of an intervention	
	*End user consultation* ^a^	Consultation with users of the program, defined as parents or caregiver program participants	
	*Stakeholder consultation*	Consultation with stakeholders associated with the program, defined as service or program providers or clinicians	
	*Academic expert consultation*	Consultation with academics with expertise in the program’s content	
	Testing	Processes or activities which allow users to engage with a prototype, and provide input about the experience of using the intervention	
	*End user testing*	Testing with users of the program, defined as parents or caregiver program participants	
	*Stakeholder testing*	Testing with stakeholders associated with the program, defined as service or program providers or clinicians	
	*Academic expert testing*	Testing with academics with expertise in the program’s content	
**Enrollment strategies**			
	Targeted recruitment strategy	Efforts to increase the likelihood that the intended population or sample will be recruited^b^	
	Partnerships	Efforts to collaborate with and involve relevant communities or services in the recruitment of intervention users^b^	
	Practical support	Provision of services or materials that facilitate users to use and engage with the program (eg, loaning of technological equipment, technical assistance, provision of childcare).	
**Program-specific strategies**			
	Content- behavior change techniques	Tools, features, or strategies used in the intervention to promote behavior change	
	*Goal-setting*	Encourages the user to list and set goals relevant to the intervention’s content^b^	
	*Action plans*	Supports the user to list specific behaviors or actionable strategies that they intend to complete^b^	
	*Feedback*	Allows the user to receive feedback (may be automated or via an interventionist)^b^	
	*Self-monitoring tools*	Tracks a user’s performance or status to support achieving goals^b^	
	Content: rewards	Features that offer reward upon user’s performance of a target behavior	
	*Praise*	Offers praise to the end user on any occasion^b^	
	Content: summaries	Features which provide the user with a summary of intervention content or activity^b^	
	Content: social support features	Features that facilitate the receipt of social support	
	*Web-based discussion forums*	Provides the opportunity for users to see other users use the intervention or performing the target behavior^b^	
	Content: supplementary resources	Features which centralize a range of resources relevant to the user or intervention^b^	
	Content: reminders	Features that serve to remind users about using the intervention or performing target behaviors	
	Delivery: mode	Intentional use of a given technology mode, thought to enhance engagement, to deliver intervention content^b^	
	Delivery professional support features	Features that enable remote contact with a professional	
	*Clinical support*	Provides clinical, coaching, or therapeutic support to facilitate behavioral change^b^	
	*Nonclinical support*	Provides nonclinical support to facilitate use, eg, technical, encouragement, progress monitoring^b^	
	Delivery: control features	Features that make users feel in control of and free to make choices about how to interact and use the intervention	
	*All-at-once*	Users receive the whole intervention at once and are not required to complete predetermined steps to progress through the intervention	
	*Reviewability*	Users have the ability to review old content^b^	
	Delivery: tunneling	Features that lead users through predetermined steps to progress through intervention	
	Delivery: novelty	Features that provide the user with regular content updates	
	Delivery: ease of use	Features that support the use of the intervention to feel natural	
	Delivery: personalization	Features that deliver content in a way that is adapted to the user on an individual level	
	Delivery tailoring	Features that deliver content in a way that is adapted to factors relevant to the user’s potential needs, interest, use context or other factors relevant to the user’s social group	
	Delivery: message tone	The terminology and wording used to communicate the content’s message	
	*Credibility features*	Features whose presence is designed to inculcate a feeling of trust or familiarity within the user	
	*Narrative*	The presence of a storyline to deliver intervention content or concepts	
	Delivery: esthetics and design	Visual features that are designed to be attractive to users	
	Delivery: guidance	Features that provide the user with tutorials or how-to-use guides to inculcate feelings of comfort and ease of use	
	Delivery: interactivity	Features that promote a two-way flow of information between the intervention and its user	
	*Rehearsal*	Features that invite the user to rehearse a behavior or content of the intervention	
	*Challenge*	Features that invite the user to test or apply their knowledge of program concepts^b^	
	*Gamification*	Features that stimulate users to compete with themselves or the program in achieving a target behavior	
	*Reflection*	Features that invite the user to reflect on the program content or their behavior^b^	
**Research involvement strategies**			
	Reminders	Features that serve to remind users about using or completing the program’s research component (eg, questionnaires, or measures)	
	Rewards and incentives	Features that offer reward upon user’s use or completion of the program’s research component (eg, questionnaires, or measures)	

^a^Strategy subtypes are italicized.

^b^Defined by authors.

Engagement outcomes were identified as measures of engagement. Measures were categorized by the component of engagement and defined with reference to prior research [28,31,37]. Components were also defined with reference to prior research both from the parenting intervention literature that describes behavioral components (*initial, ongoing, and quality*) [28] and the technology-assisted intervention literature that describes experiential components (*qualitative*) [31,37]. An overview of the engagement outcome measure categories and definitions, organized by components of engagement, is provided in Table 2.

Additional outcomes for studies included in the secondary analysis included between-group statistical analyses and associated *P* values for engagement outcomes and target ACE outcomes.

**Table 2:** Engagement outcome measures categorized by phases of program engagement or as qualitative measures.

Component of engagement and measure category		Definition
**Initial**		Parents’ intent or actual enrollment in a program
	Recruitment rates	Rates of parents recruited per recruitment site per month
	Enrollment rates	Rates of parents enrolling in the program
	Expressions of interest	Rates of parents expressing interest in enrolling in the program
**Ongoing**		Parents’ behavioral engagement with the program or session or module as a whole
	Ongoing frequency	Measures that provide information on how often a user visits the program
	*Attendance* ^a^	Visits indicated by attendance to a session
	*Log-ins*	Visits indicated by log-ins to a module
	Ongoing intensity	Measures that provide information on the depth of users’ engagement within the program
	*Session or module interaction*	Measures that provide information on users’ interaction with interactive features within sessions or modules.
	Ongoing completion rates	Measures that indicate when a user has completed the program or a session or module
	*Program*	Measures that indicate when a user has completed the entire program
	*Session or module*	Measures that indicate when a user has completed a session or a module in the program
	Ongoing time or duration	Measures of the duration of engagement during a visit to the program or the session or module
	*Program*	Measures of duration of engagement during the entire program
	*Session or module*	Measures of duration of engagement during a session or a module
	Retention, attrition, or dropout rates	Measures indicating users’ status of engagement with the treatment, program or study
	*Treatment or program*	Measures indicating users’ status of engagement with the treatment or program itself
	*Study*	Measures indicating users’ status of engagement with the study in which the treatment or program is delivered
**Quality**		Parents’ behavioral engagement with the programs or session or module’s specific components
	Intensity of specific component use	Measures that provide information on the depth of users’ engagement with specific components within the program or session or module
	Completion rates of specific components	Measures that indicate when a user has completed specific components within the program or a session or module
	Time or duration spent in specific components	Measures of the duration of engagement during a visit to a specific component within the program
	Adherence	Measures the extent to which the program is engaged with as intended or agreed by the parent or program developer
**Qualitative**		Parents’ subjective feedback regarding their experience of engaging with the program
	Satisfaction measures	Captures extent to which parent perceives the program met their needs and expectations
	Feedback measures	Captures a range of parents’ reactions or opinions about the program
	Semistructured interviews	Captures parents’ experiences and feelings in relation to using the program
	Think-aloud	Captures parents’ experiences of using the program in real time
	Focus groups	Captures social and contextual factors in specific population subgroups that influence engagement with the program
	Other	Other measures of subjective feedback

^a^Measure category subtypes are italicized.

### Methodological Quality Assessment and Appraisal

The included studies were found to use qualitative, quantitative, and mixed methods for reporting on and evaluating interventions. Therefore, the Mixed Methods Appraisal Tool (MMAT; version 2018; [38]) was used to analyze the methodological quality of the included studies. The MMAT includes 2 screening items to check whether a study reports on empirical data, and 5 subsequent items (which differ depending on the category of empirical study design selected) to assess the methodological quality of empirical studies. The MMAT’s 2-item screener and 5-item quality criteria were included in the prepiloted extraction template. Each included study that met the 2-item screener was then rated according to the 5-item MMAT scoring criteria (Multimedia Appendix 2 provides the methodological quality assessment decision rules). As recommended by the MMAT, the spread of ratings was interpreted per criterion to better summarize the quality of the included studies (rather than an overall score per criterion). If an included study did not meet the 2-item screener criteria, its quality could not be assessed. However, it was still included in the narrative synthesis for answering this review’s primary aim but was excluded from the quantitative synthesis for answering this review’s secondary aim. Risk of bias was independently assessed by 2 researchers, with the first author (GA) assessing all studies, and 2 researchers (AT and CN) assessing alternating studies. Discrepancies in ratings were resolved through discussion between researchers.

### Data Synthesis Methods

#### Primary Outcome

A narrative synthesis [39] was used to address the primary aim of describing the range of engagement strategies and measures used in technology-assisted parenting interventions that target ACEs and exploring any patterns to the use of engagement strategies based on the program’s level of prevention. The total number of observations of a given strategy and measure was reported along with the proportion of included studies that used a given strategy or measure.

#### Secondary Outcome

Heterogeneity in intervention features, settings, populations, and statistical tests did not allow a meta-analysis of effect sizes to be conducted. When studies examine a common variable, but results are represented by a variety of effect magnitude measures, combined significance tests are indicated [40]. The Stouffer method of combining *P* values [41] was used to synthesize results from studies eligible for the secondary outcome analysis. Where there were ≥2 independent observations of the association between the same engagement strategy and engagement outcome category in the included studies, the Stouffer method was applied to test the combined significance of this association. (all subcategories for each engagement strategy were included in pairs, as all subcategories fall under the same definition). Stouffer *z* was also applied to test the combined significance of associations between engagement outcomes and target ACE outcomes, where ≥2 independent observations of this association were identified. Stouffer *z* was calculated by dividing the sum of the *z*(*p_i_*) values by the square root of *k*, where *k* is the number of associations per pairing of the engagement strategy and outcome. If the resulting *P* value corresponded to a probability level <.01, the null hypothesis of no effect was rejected.

Before analysis, the engagement outcomes were coded into discrete types and measures for consistency (Table 2). Raw *P* values were converted to 1-tailed values before analysis to test the directional hypothesis that engagement strategies significantly increase engagement. Direction of effect was assumed toward the experimental group, given the rationale provided in all studies for testing engagement strategies, based on theory or evidence that they might have an effect.

## Results

### Study Selection

Figure 1 provides the flow of the systematic search process. A total of 13,973 records were identified by searching electronic databases, which were reduced to 6377 records after removal of duplicates and the postsearch decision to exclude studies published before 2010. Of the 6377 records whose titles and abstracts were screened, 290 (4.55%) full texts were assessed for eligibility and 147 (2.3%) were excluded (Figure 1 provides the reasons for exclusion). Manual searching of the reference lists of both relevant systematic reviews identified in the database search and included studies revealed 8 additional records that met the study criteria. A gray literature search was conducted (Multimedia Appendix 1 provides the search strategy and results); 415 records were identified, 33 records were retrieved and screened, and 10 full-text records were assessed for eligibility. Five reports were excluded (Figure 1), leaving 5 additional reports that met the study criteria. A total of 156 records were included in the review, comprising 127 separate studies (Multimedia Appendix 3 indicates which records were merged). The included studies are summarized in the *Characteristics of Included Studies* section using narrative synthesis methods for the primary outcome and Stouffer *P* analysis for the secondary outcome. Multimedia Appendix 4 details each study’s characteristics, engagement strategies, and measures used.

### Characteristics of the Included Studies

#### Study Designs

Most of the included studies were RCTs (77/127, 60.6%), followed by nonrandomized studies that estimated the effectiveness of the program (31/127, 24.4%). Other included studies had a descriptive design (5/127, 3.9%), mixed methods design (6/127, 4.7%), and qualitative design (3/127, 2.4%).

#### Participants

Studies were conducted across 16 different countries, although most were conducted in the United States (69/127, 54.3%), followed by Australia (33/127, 26%). Programs most often catered to parents of young people with a mean age between 5 and 12 years (31/127, 24.4%), although many studies (51/127, 40.2%) did not report the age of parent or caregiver’s young person. Mothers or female caregivers represented most (ie, >80%) of the sample in just more than half (69/127, 50.4%) of the included studies, whereas fathers or male caregivers comprised most of the sample in far fewer studies (8/127, 5.5%). A very small percentage of the studies reported an even spread of male and female caregivers (6/127, 4.7%). Just more than one-third (42/127, 33.1%) of the samples in the identified studies were reported as taking place in the context of socioeconomic difficulty or vulnerability, and approximately half (61/127, 48%) did not specifically report or state participants’ socioeconomic position (where possible, participant characteristics were extracted only for study participants with access to the technology-assisted intervention).

#### Programs

Most programs (97/127, 76.4%) did not report a design framework by which the program was designed or developed, although a small number reported user-centered design approaches (9/127, 7.1%). The most common ACE targeted by the programs was maladaptive parenting behaviors (98/127, 77.2%), followed by interparental conflict (19/127, 15%), and child maltreatment (10/127, 7.9%). A small percentage of the studies (6/127, 4.7%) reported targeting more than one of the target ACEs. Programs were almost equal either at the selective (48/127, 37.8%) or indicated (47/127, 37%) level of prevention, with universal programs being less common (32/127, 35.2%). The programs were primarily delivered in the participants’ home (84/127, 66.1%), and approximately a quarter of the programs were delivered at home through a health (20/127, 15.8%) or community service (11/127, 8.7%). One-fifth (26/127, 20.5%) of the programs involved the young person of the participating parent or caregiver. The programs’ technology most commonly functioned to facilitate self-directed (ie, asynchronous) learning (67/127, 52.8%), with one-third (39/127, 30.7%) of the programs combining remote clinician contact with self-directed learning (ie, synchronous). A few programs included technologies that functioned to enhance existing services (5/127, 3.9%). Most programs comprised one (54/127, 42.5%) or two (42/127, 33.1%) modes of technology in the delivery of the program, with web-based modules being the most common mode (76/127, 59.8%). Videos, videoconferencing, emails, telephone calls, and text or application messaging were also commonly used. Table 3 provides a detailed breakdown of the participants and program characteristics of the included studies.

**Table 3:** Characteristics of included studies (N=127).

Characteristics			Studies, n (%)
**Participant**			
	**Country**		
		Australia	33 (26)
		Canada	2 (1.6)
		Finland	2 (1.6)
		New Zealand	4 (3.1)
		The Netherlands	3 (2.4)
		Sweden	2 (1.6)
		United Kingdom	3 (2.4)
		United States	69 (54.3)
		Other	9 (7.1)
	**Mean age of young person at recruitment**		
		Infant age (0-12 mo)	4 (3.1)
		Toddler (1-3 y)	4 (3.1)
		Preschool age (>3-5 y)	22 (17.3)
		Primary School age (>5-12 y)	31 (24.4)
		Adolescence (>12-18 y)	15 (11.8)
		Not reported	51 (40.1)
		Age range reported	24 (19.3)
		Not applicable due to study design	5 (3.9)
	**Parent sex**		
		>80% female	69 (54.3)
		<20% female (100% male)	8 (6.3)
		50% male and female	6 (4.7)
		20%-79% female	18 (14.2)
		N/A^a^ or not reported	26 (20.5)
	**Socioeconomic position**		
		Low socioeconomic position or vulnerability	42 (33.1)
		Not reported	61 (48)
**Program**			
	**Level of prevention**		
		Universal	32 (25.2)
		Selective	48 (37.8)
		Indicated	47 (37)
	**Primary target ACEs^b^**		
		Maladaptive parenting	98 (77.2)
		Maladaptive parenting+secondary ACE	6 (4.7)
		Child maltreatment	10 (7.9)
		Interparental conflict	19 (15)
	**Target recipient**		
		Parents	96 (75.6)
		Parents (adoptive)	5 (3.9)
		Parents+young person	26 (20.5)
	**Setting**		
		Home	84 (66.1)
		Home, via health or hospital	20 (15.7)
		Home, via community or social service	11 (8.7)
		Health service or hospital	3 (2.4)
		Community setting	3 (2.4)
		Participants’ choice	5 (3.9)
		Other	1 (0.8)
	**Function of technology**		
		Self-directed learning	67 (52.7)
		Self-directed learning+remote clinician contact	39 (30.7)
		Service enhancement	5 (3.9)
		Peer support	0 (0)
		Remote clinician contact	7 (5.5)
		Remote clinician contact+peer support	1 (0.8)
		Self-directed learning+peer support	3 (2.4)
		Self-directed learning+remote clinician contact+peer support	1 (0.8)
		Other	4 (3.1)
	**Types of modes**		
		Web-based modules	76 (59.8)
		Website	8 (6.3)
		Computer, tablet, or phone apps	3 (2.4)
		Videos	25 (19.7)
		Videoconferencing	24 (18.9)
		emails	25 (19.7)
		Telephone calls	30 (23.6)
		Text or application messaging	24 (18.9)
		Podcasts	4 (3.1)
		Digital feedback	4 (3.1)
		Social media	13 (10.2)
		Multimodal; participant chooses mode	3 (2.4)
	**Total number of technology modes**		
		1	54 (42.5)
		2	42 (33.1)
		3	21 (16.5)
		4	9 (7.1)
		5	1 (0.8)
	**Design framework**		
		Community-based involvement	2 (1.6)
		User-centered design	9 (7.1)
		User-involved design	2 (1.6)
		Iterative approach	7 (5.5)
		Other	9 (7.1)
		Not reported	97 (76.4)

^a^N/A: not applicable.

^b^ACE: adverse childhood experience.

### Quality Assessment of Included Studies

All studies were assessed for quality according to the MMAT, except for 4 studies [42-45] that did not meet the MMAT screening criteria. As results from the MMAT are best understood via ratings of each study design’s criterion rather than calculating an overall score per study [38], a brief summary of the key results is provided (Multimedia Appendix 5 provides a full summary of results and results from each included study). All the criteria were met in 10% (8/77) of the included RCTs. The most commonly unmet criteria for RCT studies were insufficient outcome data (28/77, 36% of RCT studies) and insufficient adherence to assigned interventions (24/77, 31% of RCT studies). All criteria were met in 19% (6/31 of the included quantitative nonrandomized studies, with complete outcome data again being the most commonly unmet criterion, although interventions were more commonly administered as planned compared with quantitative RCT studies. Quantitative descriptive studies mostly included appropriate sampling strategies and statistical analyses for the research question, although criteria regarding sample representativeness and the risk of nonresponse bias were commonly unmet. Mixed methods studies overall adhered to the quality criteria for each method involved, but criteria regarding integration between each method’s results and divergences or inconsistencies were less commonly met. All qualitative studies met all the criteria.

### Primary Outcome

#### Engagement Strategies Used in the Design and Delivery of Programs in the Included Studies

Strategies used to influence engagement during the program’s design phase of the intervention cycle were reported in 43 studies [42-44,46-85]. The total number of strategies used per study ranged from 1 to 6 (mean 2.0). End user consultation was the most commonly used strategy (26/43, 60%), followed by user testing, stakeholder consultation, and expert consultation. Multimedia Appendix 6 provides a detailed overview of the types of strategies identified in the design phase, number of observations of each strategy, and number of studies reporting the use of each strategy (definitions for “Type of strategy” are provided in Table 1).

Strategies and program features designed to influence engagement during the delivery phase of the intervention cycle were reported in 123 studies [42,45-76,78-167]. The total number of strategies used per study ranged from 1 to 16 (mean 6.2). Interactive program features were the most commonly used strategy (85/123, 73%), followed by videos or animations providing guidance, user control features, professional support features, and behavior change techniques. Multimedia Appendix 6 provides a detailed overview of the types of strategies identified in the delivery phase, the total observations of each strategy, and the total studies reporting the use of each strategy (definitions for “Type of strategy” are provided in Table 1).

#### Overview of Measures Used in the Included Studies

Engagement was measured in 111 of the included studies [42,46,48-55,57-60,62-76,78-98,100-115,117,118,121-125, 127-146,148-159,162-167]. The most common component of behavioral engagement measured was initial engagement (83/111, 74.8%), followed by ongoing engagement (78/111, 70.3%) and quality of engagement (42/111, 37.8%). Moreover, 70.3% (78/111) of the studies used qualitative measures of engagement, which was comparable with the number of studies that used behavioral measures of ongoing engagement. Enrollment and recruitment rates were the most commonly reported measures of initial engagement, with a small number reporting expressions of interest. The most commonly used measure of ongoing engagement was session or module completion rates, followed by study retention, attrition, or dropout rates. The most used measure of quality of engagement was use of specific program components, followed by completion and time spent on specific program components. Satisfaction ratings or questionnaires were the most used measures of qualitative engagement, followed by feedback measures. Multimedia Appendix 6 provides a detailed overview of measures used in the included studies (definitions for “Component of engagement, Measure” are provided in Table 2).

### Secondary Outcome

Engagement outcomes between groups were statistically compared in 22 studies, in which 1 group received unique engagement strategies. Most studies in this subgroup were RCTs (15/22, 68%) that evaluated a program targeting maladaptive parenting (19/22, 86%) at the indicated level (10/22, 45%), involving parents only (15/22, 68%) in a home setting (14/22, 63%). The mean number of engagement strategies used was highest at the selective level of prevention (mean 10), followed by the indicated level (mean 7), and universal level (mean 7). No study evaluated measures related to initial or quality components of engagement, hence the following synthesis explores the effects of engagement strategies on the ongoing and qualitative components of engagement. Multimedia Appendix 4 details each study’s between-group engagement outcomes. Of these studies, 18 (82%) reported program engagement outcomes with *P* value data and were therefore appropriate for the analysis [49,73,75,86,87,94,97,98, 100-102,122,124,125,142,149,152].

Stouffer *P* analyses indicated that ongoing and qualitative engagement outcomes were positively and reliably associated with both user and stakeholder involvement (consultation and testing) in the program’s design, web-based formats (compared with face-to-face equivalents), provision of practical support to use the technology, personalization or tailoring program features, control features, and use of engagement strategies during the program’s design phase. Interactive program features were also reliably associated with ongoing outcomes of engagement, but not with qualitative outcomes of engagement. Clinical professional support features, videos, behavior change techniques, and reminders were not reliably associated with ongoing and qualitative outcomes of engagement. Table 4 provides a complete overview of the results.

Only 2 studies included in this subgroup statistically analyzed the relationships between engagement outcomes in the experimental group and target ACE outcomes [87,149]. Stouffer *P* analysis indicated that session completion (ongoing engagement) was positively and reliably associated with improvements in maladaptive parenting behaviors (*P*<.01). One study analyzed the relationship between parent-rated therapeutic alliance (qualitative engagement) and changes in child outcomes, but no significant association was found [142].

Among the 22 studies eligible for inclusion in the secondary analysis, 3 (14%) studies [49,87,102] reported significant positive effects of the experimental group (where unique engagement strategies were used) on changes in maladaptive parenting compared with control groups where such engagement strategies were not used. Moreover, 3 (14%) studies [73,94,149] reported nonsignificant group effects on maladaptive parenting and 5 (23%) studies reported no difference between groups on changes in maladaptive parenting [72,84,105,124] and interparental conflict [159]. One study found a significant negative effect of the experimental group (where unique engagement strategies were used) on changes in interparental conflict compared with a control group where strategies were not used [152]. Another study identified a negative effect of both experimental and control groups on interparental conflict, although this reduction was significantly lower for the experimental group than in the control group [124]. Five studies assessed engagement outcomes only and did not include a measure to assess changes in target ACE outcomes in the parenting domain [66,75,100,101,122] or included a measure that was not aligned with this study’s definition of ACEs [97]. Multimedia Appendix 4 details each study’s program effects on ACE outcomes.

**Table 4:** Findings from Stouffer P calculations between engagement strategies and measures of engagement.

Engagement strategy		Measure of engagement	
		Ongoing	Qualitative
**Design: user consultation and testing**			
	*n* of studies	3	0
	*n* of associations in Stouffer *P* value	3	0
	Stouffer *P* value	<.001^a^	N/A^b^
**Design: stakeholder consultation and testing**			
	*n* of studies	0	2
	*n* of associations in Stouffer *P* value	0	2
	Stouffer *P* value	N/A	<.001^a^
**Delivery mode: web based**			
	*n* of studies	6	9
	*n* of associations in Stouffer *P* value	7	10
	Stouffer *P* value	.001^a^	<.001^a^
**Practical support**			
	*n* of studies	5	6
	*n* of associations in Stouffer *P* value	5	6
	Stouffer *P* value	<.001^a^	<.001^a^
**Delivery: interactivity**			
	*n* of studies	4	5
	*n* of associations in Stouffer *P* value	4	6
	Stouffer *P* value	<.001^a^	.015
**Delivery: professional support features (clinical)**			
	*n* of studies	4	0
	*n* of associations in Stouffer *P* value	4	0
	Stouffer *P* value	.02	N/A
**Delivery: guidance (videos)**			
	*n* of studies	3	3
	*n* of associations in Stouffer *P* value	3	4
	Stouffer *P* value	.04	.04
**Content: behavior change techniques**			
	*n* of studies	3	4
	*n* of associations in Stouffer *P* value	3	4
	Stouffer *P* value	.08	.02
**Delivery: personalization or tailoring**			
	*n* of studies	3	2
	*n* of associations in Stouffer *P* value	3	2
	Stouffer *P* value	<.001^a^	<.001^a^
**Content: reminders**			
	*n* of studies	3	3
	*n* of associations in Stouffer *P* value	3	3
	Stouffer *P* value	.08	.03
**Delivery: control features**			
	*n* of studies	2	4
	*n* of associations in Stouffer *P* value	2	4
	Stouffer *P* value	.01^a^	<.001^a^

^a^Indicates significance (*P*<.01).

^b^N/A: not applicable.

## Discussion

### Principal Findings

This review aimed to systematically describe the range of engagement strategies and measures used in the design and delivery of technology-assisted parenting programs targeting ACEs related to modifiable parental behavior. A secondary aim of this review was to synthesize the findings from studies that examined the effects of using specific engagement strategies on engagement outcomes and explore any patterns in the associations between engagement outcomes and ACE outcomes. The following discussion synthesizes the primary and secondary outcome findings for the most commonly identified engagement strategies and other strategies included in the secondary outcome analysis. The available data did not permit the synthesis of engagement strategies and the initial and quality of engagement outcomes in the secondary outcome analysis, hence this discussion explores patterns of associations between engagement strategies and ongoing and qualitative engagement outcomes.

### Summary of Evidence

#### Use of Engagement Strategies in Technology-Assisted Programs Targeting Parent-Behavior ACEs

##### Design Strategies

Just more than one-third of the studies reported using a strategy in the program’s design phase to enhance engagement during the program’s delivery. This finding is comparable with the results from the systematic review of technology-assisted parenting programs by Hansen et al [20], which found that just more than one-third of the RCTs reported strategies to enhance engagement during the program’s design phase. In addition, this review found that approximately 1-2 strategies were used on average per study, with program user consultation and testing being among the most commonly reported strategies. Programs that involved users and stakeholders in their design were more likely to better engage users in their ongoing use and subjective experience of the program, respectively, compared with programs developed without these strategies. This finding supports the central claim of user-centered design approaches in that incorporating users’ unique experiences and knowledge into the design of a program is likely to increase acceptability and relevance to other parents during the program’s delivery [168].

##### Delivery Strategies

This review found that the use of program-specific engagement strategies or engaging program features reported in the delivery of a program was significantly more common than the use of design strategies, with studies using approximately 7 strategies on average. Although previous reviews exploring the use of engagement strategies in technology-assisted programs for parenting [20] and mental health [169] did not report the number of strategies used per study, it appears that the average number of strategies identified in this review was higher than the number of strategies identified in previous reviews. The review by Hansen et al [20] defined engagement strategies a posteriori, whereas the review by Saleem et al [169] defined engagement strategies a priori using the same conceptual framework as this study. This review used a conceptual framework to identify engagement strategies a priori as well as to identify and categorize additional strategies a posteriori. This approach allowed identification of strategies reported by the study’s authors and strategies that were not reported but were consistent with the conceptual framework, which may explain the higher number of strategies identified in the studies included in this review.

*Interactivity* was the most commonly used strategy, often in the form of challenges posed by the program to the user such as multiple-choice quizzes, check-in questions, or problem-solving exercises. Programs with interactive features were more likely to better engage users in their ongoing use (indicated by greater attendance, lower treatment dropout, greater module log-ins, and completion) compared with programs without these features. However, users’ subjective experience of programs with these features was not significantly different from the experience of users in a program without these features. Perski et al [31] identified interactivity as primarily having a hypothesized influence on engagement. Findings from this review provide preliminary support for the reliability of the influence that interactivity has on a user’s ongoing engagement in a technology-assisted parenting program.

*Guidance provided by videos* in the form of roleplays or vignettes was often used for demonstrating or modeling skills to users. Videos were not, however, more likely to better engage users in their ongoing use or subjective experience of a program compared with programs without videos. Interestingly, a reliable positive association between ongoing program engagement and target ACE outcomes (maladaptive parenting) was found in 2 programs [87,150] that included guidance provided by videos. According to social cognitive theories, demonstration and modeling are the key mechanisms by which learning occurs [170]. Therefore, it is likely that video guidance might be more relevant to measures of the *quality* of engagement (ie, what parents specifically invest in and receive from the program), which in turn may form a key mechanism for change [28,29]. No study with video guidance measured parents’ *quality* of engagement, hence this hypothesis could not be explored.

*Control features* frequently referred to features that allowed the parent to choose how they engage with the program, such as self-selection of modules or topics, and the ability to self-pace (which often took the form of delivering the content “all-at-once” or in a way that permitted end users to review content). Control features such as self-pacing and ability to review content were found overall to better engage users in their ongoing use of a program compared with programs without these features. However, this finding should be interpreted with caution as there were only 2 studies included in this analysis, one of which reported that users of a program with control features were *less* likely to engage compared with users of a program without such features [124]. Control features were also more likely to improve users’ subjective experience of a program. This finding is consistent with recent research with parents from a low socioeconomic background, which suggests that convenience and flexibility were key preferences for engaging with technology-assisted programs [171,172]. These results suggest that greater freedom in choosing how to engage with the program may enhance parents’ perceived benefit and satisfaction.

*Professional support features* were most frequently clinical in nature, such as coaching or therapist support, or professional facilitation of other clinical interventions (eg, groups or forums). The presence of professional support features was not found to better engage users with a program compared with programs without the presence of a professional. This finding does not support findings from similar reviews on digital health or mental health programs for adults [31,169], both of which found that professional support features or coaching had positive influences on ongoing measures of engagement (eg, number of log-ins and time spent on the internet). One key difference between these reviews and the current review is that programs in the current review targeted parents, however parents from low socioeconomic backgrounds have previously identified professional support features as a potentially engaging feature for technology-assisted programs [171]. Interestingly, one study in this analysis that reported a positive link between professional support and engagement also involved user consultation as a design strategy. It is possible that consulting parents about the type of professional support they would like to receive may positively influence ongoing engagement. Further, data from the included studies in this review did not permit the assessment of *professional support features’* effects on the *quality* of parents’ engagement (such as adherence) or their *qualitative* engagement (such as therapeutic alliance), hence it remains to be seen whether *professional support features* may influence parents’ engagement in other ways. Closer inspection of the studies included in this review revealed that parents who used a program with professional support features were not more likely to improve target ACE outcomes compared with parents who did not receive professional support features (Multimedia Appendix 4). This finding is consistent with previous reviews of technology-assisted parenting programs, which indicated that additional support did not make programs more effective in changing young people and parent outcomes than those without such support [21,24,30]. Spencer et al [21] argued that this suggests programs both with and without professional support may be beneficial, whereas Florean et al [24] argued that given prior research has highlighted the importance of professional support, and the number of studies in their meta-analysis was small, the lack of effect they identified should be interpreted with caution. Overall, further research is required to clarify whether professional support features can enhance both engagement with technology-assisted parenting programs and parenting outcomes. This may be achieved by designing professional support features that suit parents’ contexts based on parent user consultation and measuring the effects of *professional support features* on the *quality* of parents’ engagement or their *qualitative* engagement. These measures are more closely related both to what parents specifically invest in and receive from a program and to their experience of receiving professional support, respectively.

*Behavior change techniques* most frequently took the form of feedback (eg, as a tailored report or in response to interactive challenges) and goal setting. Including behavior change techniques in programs was not found to better engage users in their ongoing use and subjective experience of a program. However, the effects of behavior change techniques may be more relevant to target ACE outcomes than engagement outcomes, given that they are specifically designed to promote change in target parenting behavior. Inspection of the studies included in this analysis (n=3) found that all programs with behavior change techniques were more likely to improve target ACE outcomes compared with programs without behavior change techniques [47,122,147].

*Delivery mode* being web based, compared with a face-to-face mode of delivery of the same program, was more likely to engage parents in their ongoing use and subjective experience of a program. Up to 10 studies were included in this analysis, which enhanced the robustness of this finding. This suggests that technology-assisted programs’ ability to overcome a range of barriers for attending and completing sessions (ongoing engagement) is likely to enhance parents’ perceived acceptability and satisfaction (qualitative engagement) with the program. Although half of the studies included in this analysis did not measure ACE outcomes, the studies that did generally find no differences between web-based programs compared with programs delivered face-to-face. To our knowledge, this is the first review to directly compare both engagement and ACE outcomes between web-based programs versus programs delivered face-to-face. Our findings suggest that web-based programs were as effective as face-to-face programs in improving ACE outcomes related to modifiable parenting behavior; however, engaging in a web-based program may have been a more positive experience than attending face-to-face.

*Personalization or tailoring* strategies often included content recommendations based on user data, or artifacts that permitted the user to personalize their experience (eg, scrapbooks or journals). The review found that these strategies were more likely to better engage parents, consistent with prior research [31,169], providing further support for personalizing or tailoring parenting programs. Although *practical support* for using the technology-assisted components appears to have generally been included for research purposes, this review found that programs involving *practical support* were more likely to engage parents, indicating that this may be an important strategy to enhance continued engagement with a parenting program. For example, one low-cost strategy to provide *practical support* was to provide instructions, user manuals, or orientation sessions on using the technology.

Finally, this review found that the use of reminders was not more likely to engage with parents’ ongoing use or subjective experience of a program. Previous reviews have found mixed support for the use of reminders in engaging users [173,174]. This review found that *control features* and *personalization or tailoring* were more likely to improve engagement, hence offering parents control over how they receive reminders and personalizing or tailoring reminders may increase their relevance and enhance engagement.

#### Use of Engagement Strategies Across Levels of Prevention

Programs at the level of selective prevention were found to use a higher number of engagement strategies, whereas the use of engagement strategies was roughly equal between programs at the level of indicated and universal prevention. Prevention strategies are most effective when they account for variation in families’ needs, as well as families and services’ available resources and capacities [33]. Given programs at the indicated prevention level are more intensive, they may require more intense effort and greater use of strategies to promote parental engagement. All programs at the indicated level included professional support features, whereas professional support features were much less frequently included for programs at the selective level and not at all for programs at the universal level. This association may be because fewer strategies were needed to deliver programs at the indicated level owing to the presence of a clinician facilitating and supporting engagement, whereas programs delivered at the selective level made use of other features to facilitate and support parents whose young people are identified as “at risk” in self-directed learning and engagement. On the basis of the findings from this review, parents’ ongoing engagement with self-directed learning programs aimed at selective or universal prevention may be enhanced by using personalization or tailoring strategies, control features, and interactivity.

### Measures

This review found that most studies in this review reported measuring initial engagement in a program, which was likely because most studies were RCTs where reporting on measures of initial engagement or recruitment rates is required [175]. Technology-specific engagement measures, such as frequency of use, time or duration spent in the technology-assisted component, and interaction with components (eg, modules viewed and links clicked), were relatively low. However, studies including such measures tended to include >1 measure, which may in fact provide greater insight into engagement compared with focusing on one measure or domain [37]. Qualitative measures of engagement were most often in the form of satisfaction and feedback measures, which are efficient methods for understanding the perceived acceptability and usefulness of programs, evaluating the program’s acceptability and usefulness from a user perspective, and informing continued program development and refinement [37]. A very small number of studies (n=16) used measures to explore parents’ subjective experience, such as semistructured interviews, focus groups or “think aloud” techniques. Although such measures may require greater time resources, they are likely to support users to reflect on how the program affected changes in target domains. Such data may assist researchers and program developers to better understand the features or components that are specifically related to behavior change. The number of studies that measured the quality of engagement was approximately half the number of studies that measured other domains of engagement. Given that the quality of parents’ engagement is suggested to be a key mechanism for positive parenting change [76], more frequent evaluation of engagement quality is required to extend our understanding of engagement and associated change in target outcomes in technology-assisted programs.

### Strengths and Limitations of This Review

To our knowledge, this is the first systematic review of technology-assisted parenting programs to comprehensively assess and describe the use of engagement strategies and measurement of engagement outcomes. Unlike previous reviews, which have identified specific strategies to assess a priori, this review did not place any restrictions on the type of program engagement strategy or outcome, and furthermore, used a highly inclusive approach to study selection. This permitted identification of a broad range of technology-assisted parenting programs, and subsequently a broad range of strategies and measures were identified for synthesis. Findings from this review were also reported using an existing conceptual framework, as use of a shared terminology can support both advancements in understanding and integration of knowledge across disciplines. Although a meta-analysis was not possible in this review, the range of strategies and outcomes identified through an inclusive approach meant that it was possible to perform a quantitative synthesis of associations between engagement strategies and engagement outcomes. This extends the current evidence, which to date has been narratively synthesized [20]. Overall, the methodology in this review permitted a comprehensive description and preliminary quantitative assessment of engagement strategies used in technology-assisted parenting programs. This in turn is a step toward responding to calls in the wider digital health literature for assessment of all available engagement strategies to generate more robust evidence, as well as contributing to a reduction in conceptual and empirical fragmentation in digital health research [31,32].

Several limitations of the current review should be noted. First, although the use of existing conceptual frameworks [28,31,37] supported consistent definitions of identified engagement strategies and measures, some strategies and measures required new definitions based on available information from included studies. Such information was not consistently clear or adequate, hence these definitions and results associated with these strategies and measures should be interpreted with this caveat in mind. Second, available data for associations between engagement strategies and engagement outcomes were heterogeneous both in program delivery and statistical analysis. This precluded meta-analysis to estimate effect sizes, so the Stouffer *P* method was used to estimate the reliability of these associations. This method is argued to be limited by its inability to weigh studies according to their sample size, although some authors argue that *P* values are already weighted, as the *P* value itself depends on the sample size for which it is calculated [176]. However, available data for associations between engagement strategies and engagement outcomes were limited. The resulting small sample size of the studies included in the Stouffer *P* analyses should be considered when interpreting the reliability of these associations. Another critique of *P* values is that they are less clinically relevant than other measures of statistical inference such as effect sizes and CIs. However, given that engagement is not specifically a clinical outcome, we considered use of *P* values to demarcate significant change as appropriate in this context. Third, although the quality assessment of the included studies was primarily to provide a summary of the quality of the available literature, the overall quality of the studies included in the quantitative analysis was low. Most notably, just more than half of the RCTs (42/77, 55%) reported sufficient complete outcome data, so reported *P* values for engagement outcomes may have been biased by completer versus noncompleter characteristics. Fourth, fewer than half (32/77, 42%) of the study participants sufficiently adhered to the assigned program. The cutoff value applied for acceptable adherence to the program in this review was based on findings from a previous review that examined adherence to technology-assisted parenting programs [20], however this cutoff may be above the norm for technology-assisted parenting programs (see the *Recommendations for Future Research* section for further elaboration). Finally, samples of parents in the included studies were overwhelmingly mothers or female participants, underscoring the well-recognized need for better representing fathers or male caregivers in technology-assisted parenting program development and evaluation [177].

### Recommendations for Future Research

This review identified a broad range of engagement strategies that future research can draw on in the design and delivery of technology-assisted parenting interventions. The findings from this review also suggest that consulting and testing program components with parents in the design phase of a program may lead to better engagement outcomes. It is recommended that future research meaningfully involves parents in the program’s design to more effectively identify strategies that will be perceived as acceptable and useful in the delivery phase of a program, and hence enhance engagement. Furthermore, the process of engaging, incorporating, and triangulating multiple stakeholder perspectives may uncover assumptions about engagement within the literature and potentially advance the understanding of why technology-assisted parenting programs are frequently undermined by poor engagement.

Heterogeneity in measuring and evaluating engagement has been cited as precluding meta-analysis of engagement outcomes in technology-assisted parenting programs [20]. This review integrated measurement concepts from the digital intervention and parenting intervention literature to synthesize heterogeneous measures in a conceptually meaningful way, which allowed a preliminary quantitative synthesis. To continue reducing heterogeneity in measuring and evaluating engagement, future studies of technology-assisted parenting programs may consider these integrated measurement concepts in selecting a range of measures, at various stages of engagement, appropriate to a given research question. Future studies should also consider selecting nonbehavioral measures of engagement to complement behavioral measures to sufficiently capture the full experience of engaging in a technology-assisted parenting program.

Of the 87 studies whose research design involved a treatment or experimental group and a comparison group, only 22 (25%) performed statistical comparisons of parents’ engagement outcomes. Low proportions have been reported in other reviews of engagement in digital interventions in the mental health field [169]. To respond to calls for a better understanding of *how* technology-assisted parenting programs work, future research should consider using experimental study designs and statistical between-groups comparisons of both intervention *and* engagement outcomes and report standardized effect sizes to facilitate meta-analysis of engagement outcomes. Such data can extend our knowledge of the effects of specific engagement strategies on engagement outcomes, as well as better understand the associations between engagement and intervention outcomes. Yardley et al [32] argued that promoting “more engagement” in technology-assisted programs may not always be associated with positive intervention outcomes, as greater demands on a user to engage with an intervention may lead to user burden and fatigue [31]. Current standards for assessing sufficient engagement on outcomes such as attrition or adherence may therefore be too high. Therefore, this knowledge may also contribute to better understanding of what an optimal dose or “effective engagement” [32] looks like in technology-assisted parenting programs. Better understanding of sufficient engagement with a program to accomplish desired effects may have implications for how these concepts are evaluated in future studies.

### Summary and Conclusions

This review describes and appraises the range of engagement strategies and measures used in the design and delivery of technology-assisted parenting programs targeting ACEs that are within parents’ capacity to modify. Preliminary evidence was found for including involvement of users and stakeholders in the program’s design, personalization or tailoring features, control features, and provision of practical support for enhancing ongoing and qualitative outcomes of engagement. Preliminary evidence was also found for the notion that web-based parenting programs are effective in promoting ongoing engagement, which in turn may enhance overall satisfaction. Engagement strategies that were not found to enhance ongoing or qualitative engagement outcomes (ie, professional support features, videos, and behavior change techniques) may be related to the quality of parents’ engagement in a program. Using a broad range of engagement measures to sufficiently capture parents’ experience of engagement in a technology-assisted parenting program and statistically comparing engagement outcomes between groups receiving different programs to facilitate meta-analysis can advance current knowledge on the potential effects of specific strategies on engagement outcomes. However, such knowledge should serve to complement knowledge about user context when designing technology-assisted parenting programs [32,168]. There is much yet to learn about the relationship between engagement in technology-assisted parenting programs and change in target ACE outcomes, but efforts to better understand this potential mechanism for change hold significant implications for preventing or reducing the impact of ACEs at the family level and associated mental health outcomes of young people.

## Appendix

Multimedia Appendix 1 Search strategies.

Multimedia Appendix 2 Decision rules.

Multimedia Appendix 3 Additional references merged with main references.

Multimedia Appendix 4 Results of individual studies.

Multimedia Appendix 5 Results of quality assessment.

Multimedia Appendix 6 Primary outcome tables.

Multimedia Appendix 7 PRISMA checklist.

## Abbreviations

ACE: adverse childhood experience
MMAT: Mixed Methods Appraisal Tool
PRISMA: Preferred Reporting Items for Systematic Reviews and Meta-Analyses
RCT: randomized controlled trial
