# Supplementary material for: Engaging Parents in Technology-Assisted Interventions for Childhood Adversity: Systematic Review
Source: J Med Internet Res. 2024 Jan 19;26:e43994. doi: 10.2196/43994 (PMC10837762; doi:10.2196/43994)
Supplement: Multimedia Appendix 1 [file jmir_v26i1e43994_app1.docx]

# Appendix 1

# Database search strategies

In the following tables, ‘*Intervention’* ‘*Technology*-*assisted’* ‘*Child’* and ‘*ACE’* concepts are combined with the ‘AND’ Boolean operator to create final search strategy that incorporates all concepts. Separate searches for each ‘*ACE’* concept were conducted.

## PsycINFO

### (MeSH headings and keywords)

| **Concept** | **MeSH Headings** | **Keywords** |
| --- | --- | --- |
| **S1 Intervention** | Intervention  Early intervention  Family intervention  Prevention  Mental health programs  Health care services  Mental health services  Evidence based practice  Psychotherapy (exploded)  Treatment (exploded)  Online therapy  Parent training  Family life education | Intervention*  Program*  Service*  Treatment*  Training*  Therapy OR therapies  Psychotherapy OR psychotherapies  Psychoeducation  Course OR courses  Behavio?ral management  Self-train OR self-training  Self-help  Self-guide OR self-guided  Initiative*  Strategy OR strategies |
| **S2 Technology-assisted** | Internet  Computer applications  Electronic communication  Electronic learning  Online therapy  Social media  Telemedicine  Websites  Computer assisted therapy | Online  Technology  Electronic  Computer  Digital OR digitise*  Mobile*  Web-base*  Website*  Internet*  Smartphone*  Cellphone*  App OR apps  e-health  m-health  u-health  telehealth  telemedicine  SMS  Text message* |
| **S3 Child** | (None) | Child  P?ediatric  Baby OR babies  Infant OR infants  Toddler*  Preteen OR pre-teen  Teen*  Adolescen*  Youth  Minors  Early life  Youngster* |
| **S4a ACE** (maladaptive parenting) | Parenting  Authoritarian Parenting  Permissive Parenting  Parenting Styles | Parenting*  AND  Negative AND behaviour  Maladaptive  Authoritarian  Permissive  Insecure attachment  Aversive*  Inconsistent  Over-involvement  Harsh  Withdraw* |
| **S4b ACE** (child abuse) | Child abuse  Child neglect  Emotional abuse  Physical abuse  Sexual abuse  Verbal abuse | Mental*  Emotional*  Psychological  Corporal  Physical*  Sexual*  Abus*  Punishment  Punitive*  Assault*  Maltreat*  Neglect* |
| **S4c ACE** (inter-parental conflict) | Marital Conflict  Family Conflict  Domestic Violence  Intimate Partner Violence | Parent*  Inter-parental  Interspousal  Marital  Partner  Domestic  Famil*  AND  Conflict*  Discord*  Hostil*  Aggress*  Abus*  Violen*  OR  Intimate partner violence |

### Search strategy

S1

intervention*.ti,ab. (387037)

program*.ti,ab. (396949)

service*.ti,ab. (278950)

treatment*.ti,ab. (623243)

training.ti,ab. (260270)

(therapy or therapies).ti,ab. (253464)

(psychotherapy or psychotherapies).ti,ab. (89759)

psychoeducation.ti,ab. (4159)

(course or courses).ti,ab. (185651)

behavio?ral management.ti,ab. (904)

(self-train or self-training).ti,ab. (165)

self-help.ti,ab. (8323)

(self-guide or self-guided).ti,ab. (548)

initiative*.ti,ab. (42088)

(strategy or strategies).ti,ab. (312585)

Intervention/ or early intervention/ or family intervention/ or prevention/ (106864)

mental health programs/ or health care services/ or mental health services/ (82679)

evidence based practice/ (18119)

exp psychotherapy/ (204292)

exp treatment/ (1065894)

parent training/ or family life education/ (7498)

S2

online.ti,ab. (83756)

technology.ti,ab. (79218)

electronic.ti,ab. (29420)

computer.ti,ab. (69903)

(digital or digitise*).ti,ab. (23052)

mobile*.ti,ab. (16966)

(web base* or web-base*).ti,ab. (13421)

website*.ti,ab. (12745)

internet*.ti,ab. (38783)

smartphone*.ti,ab. (4038)

cellphone*.ti,ab. (172)

(app or apps).ti,ab. (7282)

ehealth.ti,ab. (723)

mhealth.ti,ab. (656)

uhealth.ti,ab. (0)

telehealth.ti,ab. (1422)

telemedicine.ti,ab. (1555)

SMS.ti,ab. (1510)

text messag*.ti,ab. (2299)

internet/ or computer applications/ or electronic communication/ or electronic learning/ or online therapy/ or social media/ or telemedicine/ or websites/ or Computer Assisted Therapy/ (61427)

S3

child*.ti,ab. (696160)

p?ediatric.ti,ab. (31190)

(baby or babies).ti,ab. (16086)

(infant or infants).ti,ab. (71333)

toddler*.ti,ab. (9375)

(preteen or pre-teen).ti,ab. (265)

teen*.ti,ab. (22072)

adolesce*.ti,ab. (236896)

youth.ti,ab. (89796)

minors.ti,ab. (2432)

early life.ti,ab. (7603)

youngster*.ti,ab. (3649)

S4a

1 parenting*.ti,ab. (38701)

2 (negative and behaviour).ti,ab. (6506)

3 style.ti,ab. (64654)

4 maladaptive.ti,ab. (16506)

5 authoritarian.ti,ab. (5272)

6 permissive.ti,ab. (3180)

7 insecure attachment.ti,ab. (2508)

8 aversive*.ti,ab. (15510)

9 inconsisten*.ti,ab. (40234)

10 overinvolve*.ti,ab. (496)

11 harsh.ti,ab. (3597)

12 withdraw*.ti,ab. (39998)

13 2 or 3 or 4 or 5 or 6 or 7 or 8 or 9 or 10 or 11 or 12 (190463)

14 1 and 13 (6550)

15 Permissive Parenting/ or Authoritarian Parenting/ or Parenting/ or Parenting Style/ (16575)

16 14 or 15 (19743)

S4b

1 mental*.ti,ab. (416503)

2 emotional*.ti,ab. (245585)

3 psychological*.ti,ab. (352059)

4 corporal.ti,ab. (2083)

5 physical*.ti,ab. (258002)

6 sexual*.ti,ab. (172462)

7 1 or 2 or 3 or 4 or 5 or 6 (1171451)

8 abus*.ti,ab. (130291)

9 punishment*.ti,ab. (18004)

10 punitive*.ti,ab. (4226)

11 assault*.ti,ab. (14471)

12 8 or 9 or 10 or 11 (161424)

13 ((mental* or emotional* or psychological* or corporal or physical* or sexual*) adj3 (abus* or punishment* or punitive* or assault*)).ti,ab. (44268)

Annotation: sexual abuse

14 maltreat*.ti,ab. (11907)

15 neglect*.ti,ab. (42850)

16 child abuse/ or child neglect/ or emotional abuse/ or physical abuse/ or sexual abuse/ or verbal abuse/ (43612)

17 13 or 14 or 15 or 16 (100898)

S4c

1 parent*.ti,ab. (271899)

2 interparental.ti,ab. (1283)

3 interspousal.ti,ab. (58)

4 marital.ti,ab. (39142)

5 domestic.ti,ab. (24567)

6 partner.ti,ab. (48420)

7 famil*.ti,ab. (433899)

8 conflict*.ti,ab. (126639)

9 discord*.ti,ab. (6337)

10 hostil*.ti,ab. (21430)

11 aggress*.ti,ab. (80376)

12 abus*.ti,ab. (130291)

13 violen*.ti,ab. (89801)

14 Marital Conflict/ or Family Conflict/ (5723)

15 Domestic Violence/ or Intimate Partner Violence/ (20788)

16 1 or 2 or 3 or 4 or 5 or 6 or 7 (677087)

17 8 or 9 or 10 or 11 or 12 or 13 (397066)

18 ((parent* or interparental or interspousal or marital or domestic or partner or famil*) adj2 (conflict* or discord* or hostil* or aggress* or abus* or violen*)).ti,ab. (40791)

19 14 or 15 or 18 (46809)

## OVID Medline

### (MeSH headings and keywords)

| **Concept** | **MeSH Headings** | **Keywords** |
| --- | --- | --- |
| **Intervention** | Health education  Consumer health information (Exploded)  Health promotion (Exploded)  Patient education as topic  Psychotherapy (exploded)  Early medical intervention | Intervention*  Program*  Service*  Treatment*  Training*  Therapy OR therapies  Psychotherapy OR psychotherapies  Psychoeducation  Course OR courses  Behavio?ral management  Self-train OR self-training  Self-help  Self-guide OR self-guided  Initiative*  Strategy OR strategies |
| **Technology-assisted** | Blogging  Online social networking  Mass media (exploded)  Television  Social media  Electronic mail  Telemedicine  Telephone  Cellphone  Videoconferencing  Internet  Internet-based intervention  Mobile applications  Video games  Web browser | Online  Technology  Electronic  Computer  Digital OR digitise*  Mobile*  Web-base*  Website*  Internet*  Smartphone*  Cellphone*  App OR apps  e-health  m-health  u-health  telehealth  telemedicine  SMS  Text message* |
| **Child** | (None) | Child  P?ediatric  Baby OR babies  Infant OR infants  Toddler*  Preteen OR pre-teen  Teen*  Adolescen*  Youth  Minors  Early life  Youngster* |
| **ACE** (child abuse) | Child abuse  Child abuse, sexual | Mental*  Emotional*  Psychological  Corporal  Physical*  Sexual*  Abus*  Punishment  Punitive*  Assault*  Maltreat*  Neglect* |
| **ACE** (maladaptive parenting) | Parenting | Parenting*  AND  Negative AND behaviour  Maladaptive  Authoritarian  Permissive  Insecure attachment  Aversive*  Inconsistent  Over-involve*  Harsh  Withdraw* |
| **ACE** (inter-parental conflict) | Family conflict  Domestic violence  Intimate partner violence  Spouse abuse | Parent*  Inter-parental  Interspousal  Marital  Partner  Domestic  Famil*  AND  Conflict*  Discord*  Hostil*  Aggress*  Abus*  Violen*  OR  Intimate partner violence |

### Search strategy

S1

intervention*.ti,ab. (844905)

program*.ti,ab. (751625)

service*.ti,ab. (443338)

treatment*.ti,ab. (3932756)

training.ti,ab. (341234)

(therapy or therapies).ti,ab. (1729179)

(psychotherapy or psychotherapies).ti,ab. (30530)

psychoeducation.ti,ab. (2279)

(course or courses).ti,ab. (547310)

behavio?ral management.ti,ab. (711)

(self-train or self-training).ti,ab. (277)

self-help.ti,ab. (5687)

(self-guide or self-guided).ti,ab. (471)

initiative*.ti,ab. (73188)

(strategy or strategies).ti,ab. (876271)

health education/ or exp consumer health information/ or exp health promotion/ or patient education as topic/ (219606)

exp Psychotherapy/ (197059)

early medical intervention/ (3054)

S2

online.ti,ab. (87171)

technology.ti,ab. (249840)

electronic.ti,ab. (146337)

computer.ti,ab. (182438)

(digital or digitise*).ti,ab. (99827)

mobile*.ti,ab. (78779)

web-base*.ti,ab. (24756)

website*.ti,ab. (20860)

internet*.ti,ab. (41500)

smartphone*.ti,ab. (7192)

cellphone*.ti,ab. (255)

(app or apps).ti,ab. (21991)

ehealth.ti,ab. (1916)

mhealth.ti,ab. (1817)

uhealth.ti,ab. (3)

telehealth.ti,ab. (3345)

telemedicine.ti,ab. (9062)

SMS.ti,ab. (4758)

text messag*.ti,ab. (3016)

blogging/ or online social networking/ or exp mass media/ or television/ or social media/ or electronic mail/ or telemedicine/ or telephone/ or cell phone/ or videoconferencing/ or internet/ or internet-based intervention/ or mobile applications/ or video games/ or web browser/ (174327)

S3

child*.ti,ab. (1240207)

p?ediatric.ti,ab. (272702)

(baby or babies).ti,ab. (62902)

(infant or infants).ti,ab. (336593)

toddler*.ti,ab. (9399)

(preteen or pre-teen).ti,ab. (226)

teen*.ti,ab. (27012)

adolescen*.ti,ab. (240209)

youth.ti,ab. (54395)

minors.ti,ab. (3644)

early life.ti,ab. (18370)

youngster*.ti,ab. (2289)

S4a

1 parenting*.ti,ab. (15458)

2 (negative and behaviour).ti,ab. (9604)

3 style.ti,ab. (37059)

4 maladaptive.ti,ab. (10839)

5 authoritarian.ti,ab. (1119)

6 permissive.ti,ab. (18206)

7 insecure attachment.ti,ab. (907)

8 aversive*.ti,ab. (11892)

9 inconsisten*.ti,ab. (74435)

10 overinvolve*.ti,ab. (249)

11 harsh.ti,ab. (7200)

12 withdraw*.ti,ab. (109831)

13 2 or 3 or 4 or 5 or 6 or 7 or 8 or 9 or 10 or 11 or 12 (276950)

14 1 and 13 (2531)

15 parenting/ (17017)

16 14 or 15 (17700)

S4b

41 child*.ti,ab. (1240207)

42 p?ediatric.ti,ab. (272702)

43 (baby or babies).ti,ab. (62902)

44 (infant or infants).ti,ab. (336593)

45 toddler*.ti,ab. (9399)

46 (preteen or pre-teen).ti,ab. (226)

47 teen*.ti,ab. (27012)

48 adolescen*.ti,ab. (240209)

49 youth.ti,ab. (54395)

50 minors.ti,ab. (3644)

51 early life.ti,ab. (18370)

52 youngster*.ti,ab. (2289)

53 41 or 42 or 43 or 44 or 45 or 46 or 47 or 48 or 49 or 50 or 51 or 52 (1788635)

54 mental*.ti,ab. (304871)

55 emotional*.ti,ab. (138538)

56 psychological*.ti,ab. (182393)

57 corporal.ti,ab. (2921)

58 physical*.ti,ab. (589043)

59 sexual*.ti,ab. (207018)

60 54 or 55 or 56 or 57 or 58 or 59 (1231210)

61 abus*.ti,ab. (121210)

62 punishment*.ti,ab. (6657)

63 punitive*.ti,ab. (1574)

64 assault*.ti,ab. (12190)

65 61 or 62 or 63 or 64 (138816)

66 60 and 65 (46109)

67 maltreat*.ti,ab. (6413)

68 neglect*.ti,ab. (50352)

69 Child Abuse, Sexual/ or Child Abuse/ (30653)

70 66 or 67 or 68 or 69 (109869)

71 19 and 40 and 53 and 70 (992)

S4c

1 parent*.ti,ab. (362312)

2 interparental.ti,ab. (443)

3 interspousal.ti,ab. (56)

4 marital.ti,ab. (27662)

5 partner.ti,ab. (63516)

6 domestic.ti,ab. (63712)

7 famil*.ti,ab. (948573)

8 conflict*.ti,ab. (107721)

9 discord*.ti,ab. (27943)

10 hostil*.ti,ab. (11893)

11 aggress*.ti,ab. (177879)

12 abus*.ti,ab. (121210)

13 violen*.ti,ab. (50433)

14 Family Conflict/ (2012)

15 Intimate Partner Violence/ or Domestic Violence/ (8992)

16 Spouse Abuse/ (7387)

17 1 or 2 or 3 or 4 or 5 or 6 or 7 (1363539)

18 8 or 9 or 10 or 11 or 12 or 13 (469533)

19 ((parent* or interparental or interspousal or marital or partner or domestic or famil*) adj2 (conflict* or discord* or hostil* or aggress* or abus* or violen*)).ti,ab. (21175)

20 14 or 15 or 16 or 19 (27271)

## CENTRAL

### (MeSH headings and keywords)

| **Concept** | **MeSH** | **Keywords** |
| --- | --- | --- |
| **Intervention** | Health education  Consumer health information (Exploded)  Health promotion (Exploded)  Patient education as topic  Psychotherapy (exploded)  Early medical intervention | Intervention*  Program*  Service*  Treatment*  Training*  Therapy OR therapies  Psychotherapy OR psychotherapies  Psychoeducation  Course OR courses  Behavio?ral management  Self-train OR self-training  Self-help  Self-guide OR self-guided  Initiative*  Strategy OR strategies |
| **Technology-assisted** | Blogging  Online social networking  Mass media (exploded)  Television  Social media  Electronic mail  Telemedicine  Telephone  Cellphone  Videoconferencing  Internet  Internet-based intervention  Mobile applications  Video games  Web browser | Online  Technology  Electronic  Computer  Digital OR digitise*  Mobile*  Web-base*  Website*  Internet*  Smartphone*  Cellphone*  App OR apps  e-health  m-health  u-health  telehealth  telemedicine  SMS  Text message* |
| **Child** | (None) | Child  P?ediatric  Baby OR babies  Infant OR infants  Toddler*  Preteen OR pre-teen  Teen*  Adolescen*  Youth  Minors  Early life  Youngster* |
| **ACE** (child abuse) | Child abuse  Child abuse, sexual | Mental*  Emotional*  Psychological  Corporal  Physical*  Sexual*  Abus*  Punishment  Punitive*  Assault*  Maltreat*  Neglect* |
| **ACE** (maladaptive parenting) | Parenting | Parenting*  AND  Negative AND behaviour  Maladaptive  Authoritarian  Permissive  Insecure attachment  Aversive*  Inconsistent  Over-involvement  Harsh  Withdraw* |
| **ACE** (inter-parental conflict) | Family conflict  Domestic violence  Intimate partner violence  Spouse abuse | Parent*  Inter-parental  Interspousal  Marital  Partner  Domestic  Famil*  AND  Conflict*  Discord*  Hostil*  Aggress*  Abus*  Violen*  OR  Intimate partner violence |

### Search strategy

S1

intervention*.ti,ab. (396674)

program*.ti,ab. (120116)

service*.ti,ab. (37682)

treatment*.ti,ab. (710900)

training.ti,ab. (85622)

(therapy or therapies).ti,ab. (332851)

(psychotherapy or psychotherapies).ti,ab. (6666)

psychoeducation.ti,ab. (2070)

(course or courses).ti,ab. (58741)

behavio?ral management.ti,ab. (202)

(self-train or self-training).ti,ab. (143)

self-help.ti,ab. (3034)

(self-guide or self-guided).ti,ab. (445)

initiative*.ti,ab. (5687)

(strategy or strategies).ti,ab. (77497)

health education/ or exp consumer health information/ or exp health promotion/ or patient education as topic/ (18261)

exp Psychotherapy/ (17813)

early medical intervention/ (386)

S2

online.ti,ab. (13794)

technology.ti,ab. (17029)

electronic.ti,ab. (15482)

computer.ti,ab. (23517)

(digital or digitise*).ti,ab. (11073)

mobile*.ti,ab. (8601)

web-base*.ti,ab. (7184)

website*.ti,ab. (4522)

internet*.ti,ab. (8648)

smartphone*.ti,ab. (3529)

cellphone*.ti,ab. (103)

(app or apps).ti,ab. (4181)

ehealth.ti,ab. (555)

mhealth.ti,ab. (1104)

uhealth.ti,ab. (4)

telehealth.ti,ab. (1021)

telemedicine.ti,ab. (1569)

SMS.ti,ab. (2040)

text messag*.ti,ab. (3422)

blogging/ or online social networking/ or exp mass media/ or television/ or social media/ or electronic mail/ or telemedicine/ or telephone/ or cell phone/ or videoconferencing/ or internet/ or internet-based intervention/ or mobile applications/ or video games/ or web browser/ (10808)

S3

child*.ti,ab. (130520)

p?ediatric.ti,ab. (31640)

(baby or babies).ti,ab. (7617)

(infant or infants).ti,ab. (38983)

toddler*.ti,ab. (1784)

(preteen or pre-teen).ti,ab. (26)

teen*.ti,ab. (2772)

adolescen*.ti,ab. (27903)

youth.ti,ab. (6924)

minors.ti,ab. (251)

early life.ti,ab. (1121)

youngster*.ti,ab. (167)

S4a

1 parenting*.ti,ab. (3564)

2 (negative and behaviour).ti,ab. (1131)

3 style.ti,ab. (4770)

4 maladaptive.ti,ab. (1144)

5 authoritarian.ti,ab. (43)

6 permissive.ti,ab. (342)

7 insecure attachment.ti,ab. (54)

8 aversive*.ti,ab. (835)

9 inconsisten*.ti,ab. (5473)

10 overinvolve*.ti,ab. (24)

11 harsh.ti,ab. (202)

12 withdraw*.ti,ab. (27217)

13 2 or 3 or 4 or 5 or 6 or 7 or 8 or 9 or 10 or 11 or 12 (40668)

14 1 and 13 (479)

15 parenting/ (1254)

16 14 or 15 (1603)

S4b

54 mental*.ti,ab. (44289)

55 emotional*.ti,ab. (19353)

56 psychological*.ti,ab. (30857)

57 corporal.ti,ab. (267)

58 physical*.ti,ab. (102389)

59 sexual*.ti,ab. (17570)

60 54 or 55 or 56 or 57 or 58 or 59 (177378)

61 abus*.ti,ab. (10992)

62 punishment*.ti,ab. (392)

63 punitive*.ti,ab. (61)

64 assault*.ti,ab. (620)

65 61 or 62 or 63 or 64 (11915)

66 60 and 65 (4589)

67 maltreat*.ti,ab. (430)

68 neglect*.ti,ab. (2232)

69 Child Abuse, Sexual/ or Child Abuse/ (531)

70 66 or 67 or 68 or 69 (7013)

71 19 and 40 and 53 and 70 (280)

S4c

1 parent*.ti,ab. (41863)

2 interparental.ti,ab. (42)

3 interspousal.ti,ab. (1)

4 marital.ti,ab. (2175)

5 partner.ti,ab. (6313)

6 domestic.ti,ab. (1678)

7 famil*.ti,ab. (42136)

8 conflict*.ti,ab. (8773)

9 discord*.ti,ab. (1784)

10 hostil*.ti,ab. (1255)

11 aggress*.ti,ab. (11137)

12 abus*.ti,ab. (10992)

13 violen*.ti,ab. (3251)

14 Family Conflict/ (96)

15 Intimate Partner Violence/ or Domestic Violence/ (278)

16 Spouse Abuse/ (198)

17 1 or 2 or 3 or 4 or 5 or 6 or 7 (83696)

18 8 or 9 or 10 or 11 or 12 or 13 (34915)

19 ((parent* or interparental or interspousal or marital or partner or domestic or famil*) adj2 (conflict* or discord* or hostil* or aggress* or abus* or violen*)).ti,ab. (2188)

20 14 or 15 or 16 or 19 (2308)

## EMBASE

### (Emtree headings and keywords)

| **Concept** | **Emtree** | **Keywords** |
| --- | --- | --- |
| **Intervention** | Early intervention  Health intervention  Preventive health service  Medical service  Mental health service  Psychotherapy  Psychoeducation | Intervention*  Program*  Service*  Treatment*  Training*  Therapy OR therapies  Psychotherapy OR psychotherapies  Psychoeducation  Course OR courses  Behavio?ral management  Self-train OR self-training  Self-help  Self-guide OR self-guided  Initiative*  Strategy OR strategies |
| **Technology-assisted** | Internet  Web-based intervention  Online social network  Online support group  Computer (clinical trial)  Mobile application  Mobile phone  Telemedicine  Telehealth | Online  Technology  Electronic  Computer  Digital OR digitise*  Mobile*  Web-base*  Website*  Internet*  Smartphone*  Cellphone*  App OR apps  e-health  m-health  u-health  telehealth  telemedicine  SMS  Text message* |
| **Child** | (None) | Child  P?ediatric  Baby OR babies  Infant OR infants  Toddler*  Preteen OR pre-teen  Teen*  Adolescen*  Youth  Minors  Early life  Youngster* |
| **ACE** (child abuse) | Child abuse (exploded) | Mental*  Emotional*  Psychological  Corporal  Physical*  Sexual*  Abus*  Punishment  Punitive*  Assault*  Maltreat*  Neglect* |
| **ACE** (maladaptive parenting) | Parental behaviour  Child rearing | Parenting*  AND  Negative AND behaviour  Maladaptive  Authoritarian  Permissive  Insecure attachment  Aversive*  Inconsistent  Over-involvement  Harsh  Withdraw* |
| **ACE** (inter-parental conflict) | Domestic violence  Partner violence | Parent*  Inter-parental  Interspousal  Marital  Partner  Domestic  Famil*  AND  Conflict*  Discord*  Hostil*  Aggress*  Abus*  Violen*  OR  Intimate partner violence |

### Search strategy

S1

intervention*.ti,ab. (1438923)

program*.ti,ab. (1205624)

service*.ti,ab. (712551)

treatment*.ti,ab. (6667826)

training.ti,ab. (576682)

(therapy or therapies).ti,ab. (3049393)

(psychotherapy or psychotherapies).ti,ab. (53611)

psychoeducation.ti,ab. (4656)

(course or courses).ti,ab. (940160)

behavio?ral management.ti,ab. (1088)

(self-train or self-training).ti,ab. (536)

self-help.ti,ab. (8576)

(self-guide or self-guided).ti,ab. (848)

initiative*.ti,ab. (126804)

(strategy or strategies).ti,ab. (1415453)

early intervention/ (25963)

health program/ (110890)

preventive health service/ or medical service/ or mental health service/ (103811)

psychotherapy/ (99764)

psychoeducation/ (8377)

S2

online.ti,ab. (177793)

technology.ti,ab. (442568)

electronic.ti,ab. (304107)

computer.ti,ab. (274939)

(digital or digitise*).ti,ab. (170885)

mobile*.ti,ab. (138167)

web-base*.ti,ab. (44414)

website*.ti,ab. (42145)

internet*.ti,ab. (71965)

smartphone*.ti,ab. (15947)

cellphone*.ti,ab. (564)

(app or apps).ti,ab. (40859)

ehealth.ti,ab. (3099)

mhealth.ti,ab. (3273)

uhealth.ti,ab. (32)

telehealth.ti,ab. (5808)

telemedicine.ti,ab. (15312)

SMS.ti,ab. (8188)

text messag*.ti,ab. (5580)

internet/ or web-based intervention/ (110999)

online social network/ or online support group/ (598)

computer/ct [Clinical Trial] (30)

mobile application/ or mobile phone/ (28073)

telemedicine/ or telehealth/ (33601)

S3

child*.ti,ab. (1945435)

p?ediatric.ti,ab. (507763)

(baby or babies).ti,ab. (110470)

(infant or infants).ti,ab. (490036)

toddler*.ti,ab. (14891)

(preteen or pre-teen).ti,ab. (336)

teen*.ti,ab. (42969)

adolescen*.ti,ab. (381395)

youth.ti,ab. (85491)

minors.ti,ab. (5406)

early life.ti,ab. (29512)

youngster*.ti,ab. (3757)

S4a

1 parenting*.ti,ab. (22226)

2 (negative and behavio?r).ti,ab. (61355)

3 style.ti,ab. (60169)

4 maladaptive.ti,ab. (18053)

5 authoritarian.ti,ab. (1673)

6 permissive.ti,ab. (23810)

7 insecure attachment.ti,ab. (1472)

8 aversive*.ti,ab. (16758)

9 inconsisten*.ti,ab. (114926)

10 overinvolve*.ti,ab. (346)

11 harsh.ti,ab. (11871)

12 withdraw*.ti,ab. (187528)

13 2 or 3 or 4 or 5 or 6 or 7 or 8 or 9 or 10 or 11 or 12 (487666)

14 1 and 13 (4358)

parental behavior/ or child rearing/ (18501)

14 or 15 (22294)

S4b

60 mental*.ti,ab. (502918)

61 emotional*.ti,ab. (235010)

62 psychological*.ti,ab. (330002)

63 corporal.ti,ab. (4277)

64 physical*.ti,ab. (1014439)

65 sexual*.ti,ab. (312862)

66 60 or 61 or 62 or 63 or 64 or 65 (2040975)

67 abus*.ti,ab. (184724)

68 punishment*.ti,ab. (9962)

69 punitive*.ti,ab. (2398)

70 assault*.ti,ab. (18091)

71 67 or 68 or 69 or 70 (210949)

72 66 and 71 (72687)

73 maltreat*.ti,ab. (9362)

74 neglect*.ti,ab. (80835)

75 exp child abuse/ (39991)

76 72 or 73 or 74 or 75 (170275)

77 21 and 46 and 59 and 76 (1672)

S4c

1 parent*.ti,ab. (559557)

2 interparental.ti,ab. (578)

3 interspousal.ti,ab. (69)

4 marital.ti,ab. (45196)

5 domestic.ti,ab. (92770)

6 partner.ti,ab. (100398)

7 famil*.ti,ab. (1427265)

8 conflict*.ti,ab. (169563)

9 discord*.ti,ab. (49380)

10 hostil*.ti,ab. (18603)

11 aggress*.ti,ab. (314619)

12 abus*.ti,ab. (184724)

13 violen*.ti,ab. (74700)

14 family conflict/ (4012)

15 domestic violence/ or partner violence/ (20707)

16 1 or 2 or 3 or 4 or 5 or 6 or 7 (2060476)

17 8 or 9 or 10 or 11 or 12 or 13 (768832)

18 ((parent* or interparental or interspousal or marital or domestic or partner or famil*) adj3 (conflict* or discord* or hostil* or aggress* or abus* or violen*)).ti,ab. (34227)

19 18 or 14 or 15 (42229)

## CINAHL

### (MeSH headings and keywords)

| **Concept** | **MeSH Headings/Thesauri** | **Keywords** |
| --- | --- | --- |
| **Intervention** | Early childhood intervention  Early intervention  Community mental health services  Family services  Child health services  Adolescent health services  Mental health services  Psychotherapy (Exploded)  Patient education  Health education  Parenting education | Intervention*  Program*  Service*  Treatment*  Training*  Therapy OR therapies  Psychotherapy OR psychotherapies  Psychoeducation  Course OR courses  Behavio?ral management  Self-train OR self-training  Self-help  Self-guide OR self-guided  Initiative*  Strategy OR strategies |
| **Technology-assisted** | Therapy, computer assisted  Computer assisted instruction  Telemedicine  Telehealth  Social media | Online  Technology  Electronic  Computer  Digital OR digitise*  Mobile*  Web-base*  Website*  Internet*  Smartphone*  Cellphone*  App OR apps  e-health  m-health  telehealth  telemedicine  SMS  Text message* |
| **Child** | (None) | Child  P?ediatric  Baby OR babies  Infant OR infants  Toddler*  Preteen OR pre-teen  Teen*  Adolescen*  Youth  Minors  Early life  Youngster* |
| **ACE** (child abuse) | Child abuse, sexual  Child abuse | Mental*  Emotional*  Psychological  Corporal  Physical*  Sexual*  Abus*  Punishment  Punitive*  Assault*  Maltreat*  Neglect* |
| **ACE** (maladaptive parenting) | Parenting  Parental behaviour | Parenting*  AND  Negative AND behaviour  Maladaptive  Authoritarian  Permissive  Insecure attachment  Aversive*  Inconsistent  Over-involvement  Harsh  Withdraw* |
| **ACE** (inter-parental conflict) | Domestic violence  Intimate partner violence | Parent*  Inter-parental  Interspousal  Marital  Partner  Domestic  Famil*  AND  Conflict*  Discord*  Hostil*  Aggress*  Abus*  Violen*  OR  Intimate partner violence |

### Search strategy

TI intervention* OR AB

intervention*

TI program* OR AB

program*

TI service* OR AB

service*

TI treatment* OR AB

treatment*

TI training* OR AB

training*

TI therapy OR TI

therapies OR AB therapy

OR AB therapies

TI psychotherapy OR AB

psychotherapy AND AB

psychotherapies AND AB

psychotherapies

TI psychoeducation OR

AB psychoeducation

TI course* OR AB

course*

TI behavio#ral

management OR AB

behavio#ral management

TI self-train OR AB selftrain

OR TI self training

OR AB self-training

TI self-help OR AB selfhelp

TI self guided OR AB

self-guided

TI initiative* OR AB

initiative*

TI strategy OR AB

strategy OR TI strategies

OR AB strategies

(MH "Early Childhood

Intervention") OR (MH

"Early Intervention")

(MH "Community Mental

Health Services") OR

(MH "Family Services")

OR (MH "Child Health

Services") OR (MH

"Adolescent Health

Services") OR (MH

"Mental Health Services")

(MH "Psychotherapy+")

(MH "Patient Education")

OR (MH "Health

Education") OR (MH

"Parenting Education")

S2

TI online OR AB online

TI smartphone* OR AB

smartphone*

TI cellphone* AND AB

cellphone*

TI app OR AB app OR TI

apps OR AB apps

TI "e-health" OR AB "ehealth"

TI "m-health" OR AB "mhealth"

TI telehealth OR AB

Telehealth

TI telemedicine OR AB

Telemedicine

TI "sms" OR AB "sms"

TI "text messag*" OR AB

"text messag*"

(MH "Internet") OR (MH

"Internet-Based

Intervention")

TI technology OR AB

Technology

(MH "Therapy, Computer

Assisted") OR (MH

"Computer Assisted

Instruction")

(MH "Telemedicine") OR

(MH "Telehealth")

(MH "Social Media")

TI electronic OR AB

Electronic

TI computer OR AB

Computer

TI digital OR AB digital

OR TI digitise* OR AB

digitise*

TI mobile* OR AB

mobile*

TI web-base* OR AB

web-base*

TI website* OR AB

website*

S3

TI child* OR AB child*

TI minors OR AB minors

TI "early life" OR AB

"early life"

TI youngster* OR AB

youngster*

TI p#ediatric OR AB

p#ediatric

TI baby OR AB baby OR

TI babies OR AB babies

TI infant OR AB infant

OR TI infants OR AB

Infants

TI preteen OR AB

preteen OR TI pre-teen

OR AB pre-teen

TI toddler* OR AB

toddler*

TI teen* OR AB teen*

TI adolescen* OR AB

adolescen*

TI youth OR AB youth

S4a

S1 TI parenting OR AB

Parenting

S2 TI "negative behavio#r"

OR AB "negative

behavio#r"

S3 TI maladaptive OR AB

Maladaptive

4 TI authoritarian OR AB

Authoritarian

S5 TI permissive OR AB

Permissive

S6 TI "insecure attachment"

OR AB "insecure

attachment"

S7 TI aversive* OR AB

aversive*

S8 TI inconsistent OR AB

Inconsistent

S9 TI over-involve* OR AB

over-involve*

S10 TI harsh OR AB harsh

S11 TI withdraw* OR AB

withdraw*

S12 S2 OR S3 OR S4 OR S5

OR S6 OR S7 OR S8 OR

S9 OR S10 OR S11 OR

S74

S13 S1 AND S12

S14 (MH "Parenting") OR

(MH "Parental Behavior")

S15 S13 OR S14

S4b

S58 TI mental* OR AB

mental*

S59 TI neglect* OR AB

neglect*

S60 (MH "Child Abuse") OR

(MH "Child Abuse,

Sexual")

S61 TI assault* OR AB

assault*

S62 TI maltreat* OR AB

maltreat*

S63 TI emotional* OR AB

emotional*

S64 TI psychological* OR AB

psychological*

S65 TI corporal OR AB

Corporal

S66 TI physical* OR AB

physical*

S67 TI sexual* OR AB

sexual*

S68 TI punitive* OR AB

punitive*

S69 TI abus* OR AB abus*

S70 TI punishment OR AB

Punishment

S71 S58 OR S63 OR S64 OR

S65 OR S66 OR S67

S72 S61 OR S68 OR S69 OR

S70

73 S71 N2 S72

S74 S59 OR S60 OR S62 OR

S73

S4c

1 TI parent* OR AB parent*

S2 TI inter-parental OR AB

inter-parental OR TI

interparental OR AB

interparental

S3 TI inter-spousal OR AB

inter-spousal OR TI

interspousal OR AB

interspousal

S4 TI marital OR AB marital

S5 TI partner OR AB partner

S6 TI domestic OR AB

Domestic

S7 TI famil* OR AB famil*

S8 S1 OR S2 OR S3 OR S4

OR S5 OR S6 OR S7

S9 TI conflict OR AB conflict

S10 TI discord OR AB discord

S11 TI hostil* OR AB hostil*

S12 TI aggress* OR AB

aggress*

S13 TI abus* OR AB abus*

S14 TI violen* OR AB violen*

S15 S9 OR S10 OR S11 OR

S12 OR S13 OR S14

S16 TI "intimate partner

violence" OR AB

"intimate partner

violence"

S17 (MH "Domestic

Violence") OR (MH

"Intimate Partner

Violence")

S18 S8 N2 S15

S19 S16 OR S17 OR S18

## SCOPUS

No thesauri available in SCOPUS, keywords searched as below.

### Keywords

| **Concept** | **Keywords** |
| --- | --- |
| **Intervention** | Intervention*  Program*  Service*  Treatment*  Training*  Therapy OR therapies  Psychotherapy OR psychotherapies  Psychoeducation  Course OR courses  Self-train OR self-training  Self-help  Self-guide OR self-guided  Initiative*  Strategy OR strategies |
| **Technology-assisted** | Online  Technology  Electronic  Computer  Digital OR digitise*  Mobile*  Web-base*  Website*  Internet*  Smartphone*  Cellphone*  App OR apps  e-health  m-health  telehealth  telemedicine  SMS  Text message* |
| **Child** | Child  P?ediatric  Baby OR babies  Infant OR infants  Toddler*  Preteen OR pre-teen  Teen*  Adolescen*  Youth  Minors  Early life  Youngster* |
| **ACE** (child abuse) | Mental*  Emotional*  Psychological  Corporal  Physical*  Sexual*  Abus*  Punishment  Punitive*  Assault*  Maltreat*  Neglect* |
| **ACE** (maladaptive parenting) | Parenting*  AND  Negative AND behaviour  Maladaptive  Authoritarian  Permissive  Insecure attachment  Aversive*  Inconsistent  Over-involvement  Harsh  Withdraw* |
| **ACE** (inter-parental conflict) | Parent*  Inter-parental  Interspousal  Marital  Partner  Domestic  Famil*  AND  Conflict*  Discord*  Hostil*  Aggress*  Abus*  Violen* |

### Search strategy

S1

((TITLE-ABS(intervention*)) OR (TITLE-ABS(program*)) OR (TITLE-ABS(treatment*)) OR (TITLE-ABS(service*)) OR (TITLE-ABS(therapy OR therapies)) OR (TITLE-ABS(psychotherapy OR psychotherapies)) OR (TITLE-ABS(psychoeducation)) OR (TITLE-ABS(course OR courses)) OR # 9 OR (TITLE-ABS("self-help")) OR (TITLE-ABS("self-train" OR "self-training")) OR (TITLE-ABS("self-guide" OR "self-guided")) OR (TITLE-ABS("initiative*")) OR (TITLE-ABS(strategy OR strategies)))

S2

((TITLE-ABS(online)) OR (TITLE-ABS(technology)) OR (TITLE-ABS(electronic)) OR (TITLE-ABS(computer)) OR (TITLE-ABS(digital OR digitise*)) OR (TITLE-ABS(mobile*)) OR (TITLE-ABS("web-base*")) OR (TITLE-ABS(internet)) OR (TITLE-ABS(smartphone*)) OR (TITLE-ABS(cellphone*)) OR (TITLE-ABS("app" OR "apps")) OR (TITLE-ABS("e-health")) OR (TITLE-ABS("m-health")) OR (TITLE-ABS("u-health")) OR (TITLE-ABS(telehealth)) OR (TITLE-ABS(telemedicine)) OR (TITLE-ABS("SMS")) OR (TITLE-ABS("text messag*")))

S3

((TITLE-ABS(child*)) OR (TITLE-ABS(p*ediatric)) OR (TITLE-ABS(baby OR babies)) OR (TITLE-ABS(infant OR infants)) OR (TITLE-ABS(toddler*)) OR (TITLE-ABS(preteen OR "pre-teen")) OR (TITLE-ABS(teen*)) OR (TITLE-ABS(adolescen*)) OR (TITLE-ABS(youth)) OR (TITLE-ABS(minors)) OR (TITLE-ABS("early life")) OR (TITLE-ABS(youngster*)))

S4a

(((TITLE-ABS("negative behavio*r")) OR (TITLE-ABS(maladaptive)) OR (TITLE-ABS(authoritarian)) OR (TITLE-ABS(permissive)) OR (TITLE-ABS("insecure attachment")) OR (TITLE-ABS(aversive*)) OR (TITLE-ABS(negative)) OR (TITLE-ABS(inconsistent)) OR (TITLE-ABS("over-involve*" OR overinvolve*)) OR (TITLE-ABS(harsh)) OR (TITLE-ABS(style*)) OR (TITLE-ABS(withdraw*))) AND (TITLE-ABS(parenting)))

S4b

((((TITLE-ABS(mental*)) OR (TITLE-ABS(emotional*)) OR (TITLE-ABS(psychological*)) OR (TITLE-ABS(corporal)) OR (TITLE-ABS(physical*)) OR (TITLE-ABS(sexual*))) AND ((TITLE-ABS(abus*)) OR (TITLE-ABS(punishment)) OR (TITLE-ABS(punitive*)) OR (TITLE-ABS(assault*)))) OR (TITLE-ABS(maltreat*)) OR (TITLE-ABS(neglect*)))

S4c

(((TITLE-ABS(parent*)) OR (TITLE-ABS(interparental OR "inter-parental")) OR (TITLE-ABS(interspousal OR "inter-spousal")) OR (TITLE-ABS(marital)) OR (TITLE-ABS(partner)) OR (TITLE-ABS(domestic)) OR (TITLE-ABS(famil*))) AND ((TITLE-ABS(conflict*)) OR (TITLE-ABS(discord*)) OR (TITLE-ABS(hostil*)) OR (TITLE-ABS(aggress*)) OR (TITLE-ABS(abus*)) OR (TITLE-ABS(violen*)))

## IEEE

### MeSH terms and Keywords

| **Concept** | **MeSH Headings/Thesauri** | **Keywords** |
| --- | --- | --- |
| **Intervention** | (None) | Intervention  Program  Service  Treatment  Service  Therapy  Course |
| **Technology-assisted** | e-health  social networking  e-learning  teleconferencing  collaborative tools  telemedicine | Online  Digital  Digitise*  Mobile  Web-base*  App  Apps  Text messag*  Telehealth |
| **Child** | Pediatrics | Baby  Child  Teen  Adolescent  Youth |
| **ACE** (child abuse) |  | Emotional abuse  Physical abuse  Sexual abuse  Maltreat*  Neglect* |
| **ACE** (maladaptive parenting) |  | Parenting |
| **ACE** (inter-parental conflict) |  | Domestic  Family  Partner  Marital  Spouse  AND  Abuse  Violence  Assault  conflict |

### Search strategy

S1

(((((Abstract:intervention OR program OR treatment OR service OR therapy OR course)))

S2

((((((((Abstract:online OR digital OR digitise* OR mobile OR “web-base*” OR “app” OR “apps” OR “text messag*” OR telehealth) OR Mesh_Terms:e-health) OR Mesh_Terms:social networking) OR Mesh_Terms:e-learning) OR Mesh_Terms:teleconferencing) OR Mesh_Terms:collaborative tools) OR Mesh_Terms:telemedicine)))

S3

(((Abstract:baby OR child OR teen OR adolescent OR youth) OR Mesh_Terms:pediatrics)))

S4a

((Abstract:"parenting"))

S4b

((Abstract:emotional abuse OR physical abuse OR sexual abuse OR maltreat OR neglect))

S4c

(((Abstract:domestic OR family OR partner OR marital OR spouse) AND Abstract:abuse OR violence OR assault OR conflict))

## ACM

### Keywords

No thesauri available in ACM, keywords searched as below.

| **Concept** | **Keywords** |
| --- | --- |
| **Intervention** | intervention*  program*  treatment*  service*  therapy  therapies  psychotherapy  psychotherapies  psychoeducation  course*  "self-help"  initiative* |
| **Technology-assisted** | Online  Technology  Electronic  Computer  Digital OR digitise*  Mobile*  Web-base*  Internet*  App OR apps  e-health  m-health  telehealth  telemedicine  SMS  Text message* |
| **Child** | Child  P?ediatric  Baby OR babies  Infant OR infants  Toddler*  Preteen OR pre-teen  Teen*  Adolescen*  Youth  Minors  Early life  Youngster* |
| **ACE** (child abuse) | Mental*  Emotional*  Psychological  Corporal  Physical*  Sexual*  Abus*  Punishment  Punitive*  Assault*  Maltreat*  Neglect* |
| **ACE** (maladaptive parenting) | Parenting |
| **ACE** (inter-parental conflict) | Domestic  Family  Parent  Marital  Spous*  AND  Conflict*  Abus*  Violen* |

### Search strategy

All keywords were searched in the abstract field.

S1

intervention* OR program* OR treatment* OR service* OR therapy OR therapies OR psychotherapy OR psychotherapies OR psychoeducation OR course* OR "self-help" OR initiative*

S2

online OR technology OR electronic OR computer* OR digital OR digitise* OR mobile* OR "web-base*" OR internet OR "app" OR "apps" OR "e-health" OR "m-health" OR "u-health" or telehealth OR telemedicine OR "SMS" OR "text messag*"

S3

child* OR p?ediatric OR baby OR babies OR infant OR infants OR toddler* OR preteen OR "pre-teen" OR teen* OR adolescen* OR youth OR minors OR "early life" OR youngster*

S4a

("parenting")

S4b

(mental* OR emotional* OR psychological* OR corporal OR physical* OR sexual*) AND (abus* OR punishment OR assault* OR neglect OR maltreat*)

S4c

(Domestic OR famil* OR parent* OR marital OR spous*) AND (conflict OR abus* OR violen*)

# Grey literature search strategies

# Targeted Website Browing

## Centre for Disease Control and Prevention - Research Database

The Prevention Research Center database allows users to search for previous or current research projects.

URL: https://nccd.cdc.gov/prcresearchprojects/Search/SearchCriteria.aspx

### Search strategy

Name of Host Institution: All

CDC Funding Source: All

Health Topics: Adolescent health, Community health, Healthcare Access, Healthy youth, Immigrant, refugee and migrant health, LGBTQ+ Health, Maternal Health, Mental Health, Violence prevention

Project Identifier: All

Research Setting: Behavioral healthcare, Child care center, City/Town, Community, County/Parish, (FQHC) Federally Qualified Health Center, Home, International, Jail or prison, Medical or clinical site, Place of worship, Primary care, Rural area, School or school district, Tele Health, Tribal nation or area, U.S./Mexico border, Urban area, Workplace

Project Status: All

Gender: All

Age: Infants and toddlers (0-3 years), Children (4-11 years), Adolescents (11 - 17 years), Adolescents (12 - 17 years)

Race and Ethnicity: All

Thematic Networks: All

## California Evidence-Based Clearinghouse for Child Welfare – Program Registry

URL: https://www.cebc4cw.org/registry/search/

### Search strategy

Programs with a delivery option of:

- **virtual (online, telephone, video, zoom, etc.)**

Programs for children ages **0**, **1**, **2**, **3**, **4**, **5**, **6**, **7**, **8**, **9**, **10**, **11**, **12**, **13**, **14**, **15**, **16**, **17** *or* **18**

## Parenting Research Centre – Publications

URL: https://www.parentingrc.org.au/publications/

Database of reports, submissions, evidence reviews and published research. Search filters or criteria not available; publications searched manually page-by-page.

## Australian Institute of Family Studies (AIFS)

The AIFS manages the following clearinghouses: Communities and Families Clearinghouse Australia; Australian Centre for the Study of Sexual Assault; Australian Family Relationships Clearinghouse; National Child Protection Clearinghouse; and Closing the Gap Indigenous Clearinghouse

URL: <https://aifs.gov.au/search/site/>

### Search strategy

- Search term: parenting intervention
- Filters: child abuse and neglect, child abuse and neglect prevention, child development, children and family conflict, divorce and separation, early intervention and prevention programs, engaging hard-to-reach families, evaluation, family counselling, family violence, family relationship education, fathers, Indigenous families, marriage and couples, parents, post separation parenting, sexual violence, sole parenting, step families,

# Search Engine Browsing

## Google

The first 100 results will be screened, and reports of relevant results will be retrieved. Prior to this, reports of relevant results will be cross-checked with results from database searches, to prevent duplication.

### Search strategy

allintext: online OR web-based OR ehealth "parenting intervention" or "parenting program"
