# Supplementary material for: Engaging Parents in Technology-Assisted Interventions for Childhood Adversity: Systematic Review
Source: J Med Internet Res. 2024 Jan 19;26:e43994. doi: 10.2196/43994 (PMC10837762; doi:10.2196/43994)
Supplement: Multimedia Appendix 2 [file jmir_v26i1e43994_app2.docx]

# Appendix 2

# Decision-making rules: study characteristics, quality assessment and p-value selection rules

## Primary outcome inclusion and exclusion criteria

### Study design characteristics

Include if:

1. Randomised controlled trials (including quasi-randomised)
2. Pilot studies
3. Observational studies
4. Cross-sectional studies
5. Longitudinal studies
6. Retrospective studies
7. Cohort studies
8. Qualitative studies

Exclude if:

1. Review or meta-analysis
2. Prospective with no results published

Grey literature characteristics

Include if:

1. Discussion paper
2. Protocol describing intervention development
3. Book or book chapters
4. Conference abstracts

Exclude if:

1. Prospective with no results published

### Participant

Age of parent

Include if:

1. Program targets parent >18 years, parent participants >18 years

Exclude if:

1. Parent participants <18 years

Age of child

Include if:

1. Program targets child <18 years (as described by the World Health Organisation (WHO), a child is defined as anyone under the age of 18 years)

Exclude if:

1. >50% Child participants >18years

### Intervention

1. >50% of program targets parents or caregivers of a child aged 0-18 years
2. Targets one or more of three pre-defined ACEs (maladaptive parenting, child maltreatment and interparental conflict)
3. >50% of the program content is delivered via technology
4. Includes at least one strategy used to engage families during either the program’s design phase or during the program’s delivery phase.

### Comparator

As the primary outcome is a narrative synthesis, no comparator criteria are required.

### Outcome

Include if:

1. The study describes at least one strategy used to engage families during either the program’s design phase or during the program’s delivery phase.

Exclude if:

1. No strategies to engage families in the program are described or reported.

## Secondary outcome inclusion and exclusion criteria

The same criteria for Participant and Intervention apply. Additional criteria for the Comparator and Outcome are described below.

### Comparator

Include if:

1. Comparator from study designs which compared engagement outcomes between two or more groups, but where use of engagement strategies was unique to (at least one) group (e.g. Treatment OR care OR service as usual, active control, attention control)

Exclude if:

1. Comparator group did not complete measures of engagement

### Outcome

Dependent variable: engagement measure

Include if:

1. Study describes measure as measure of engagement
2. Measure corresponds to pre-defined categories of engagement measures (See Table 2 in body of systematic review)
3. Comparison of engagement outcomes represented by a p-value

Exclude if:

1. Comparison of engagement outcomes not represented by a p-value

Independent variable: engagement strategy

Include if:

1. The study describes at least one strategy used to engage families during either the program’s design phase or during the program’s delivery phase.
2. A strategy corresponds to pre-defined categories of engagement strategies (See Table 1 in body of systematic review)

Exclude if:

1. No strategies to engage families in the program are described or reported.

## *Quality assessment rules*

### Completeness of outcome data

A cut-off value of 20% was applied to assess completeness of outcome data, as this value is traditionally associated with ‘large’ amounts of missing data*

1. For studies included in the primary outcome of this review, <20% of missing data on the primary outcome variable(s) as indicated by the study’s authors were assessed as meeting this criterion.
2. For studies included in the secondary outcome of this review, studies reporting <20% missing data on engagement outcome variables were assessed as meeting this criterion.

### Adherence

To determine an appropriate rate of adherence, findings from a previous review of technology-assisted parenting interventions were consulted**. Adherence was subsequently assessed as acceptable if: the reported usage of a program was >65% than the intended or full program, or; the mean percentage of participants who completed the total program was >60%.

* Sterne JAC, Savović J, Page MJ, Elbers RG, Blencowe NS, Boutron I, Cates CJ, Cheng H-Y, Corbett MS, Eldridge SM, Hernán MA, Hopewell S, Hróbjartsson A, Junqueira DR, Jüni P, Kirkham JJ, Lasserson T, Li T, McAleenan A, Reeves BC, Shepperd S, Shrier I, Stewart LA, Tilling K, White IR, Whiting PF, Higgins JPT. RoB 2: a revised tool for assessing risk of bias in randomised trials. BMJ 2019; **366**: l4898.

** Hansen A, Broomfield G, Yap MBH. A systematic review of technology‐assisted parenting programs for mental health problems in youth aged 0–18 years: Applicability to underserved Australian communities. Aust J Psychol. 2019 Dec 1;71(4):433–62.

## *P-value selection rules*

1. If both bivariate correlations and regression coefficients (controlling for covariates etc.) are reported, select the former
2. If non-significant *p*-values are not quoted, we allocate the association a conservative one-tailed *p*-value of 0.5
3. If significant *p*-values are not quoted, we allocate the association the minimum *p*-value required to indicate significance as stated by the study (i.e. if *p*<.05 then *p*=.05 allocated, or if *p*<.01 then *p*=.01 allocated)
4. Unless stated otherwise in the study we will assume two-tailed significance. This is due to the exploratory nature of many of the studies being reviewed
5. If a study reports data for overall mental health symptom measures, as well as separate symptom subscales, overall measures will be selected over individual subscales, as they tend to have better psychometric properties

Converting p-values to one-tailed rules

Unless specified by the study’s hypothesis, we assume the direction of effect trends towards the experimental group. This is because all studies’ rationale for testing engagement strategies is based on theory or evidence that they might have an effect. Therefore:

- H0: The engagement strategy does not affect the engagement outcome.
- H1: The engagement strategy does affect the engagement outcome.

The following rules were applied to arrive at one-tailed p-values to be standardised:

If one tailed and:

- Significant: original value was retained, and direction was specified as being in favour of experimental condition (positive value) or comparison (negative value) by looking to the accompanying statistic.
- Non-significant: original value was retained.

If two tailed and:

- Significant: direction of effect was determined by looking to the accompanying statistic.
  - If towards experimental: p/2.
  - If towards comparison: 1-(p/2).
- Non-significant: direction of effect was determined by looking to the accompanying statistic (if provided).
  - If statistic provided:
    - If towards experimental: p/2.
    - If towards comparison: 1-(p/2).
  - If statistic not provided: p/2 (this will always be 0.5).

Converting p-values to standardised z rules

- Significant p-values in the direction of this review’s hypothesis (see above) was assumed as right tailed before converting to standardised Z.
- Significant p-values not in the direction of this review’s hypothesis (see above) were assumed as left-tailed before converting to standardised Z.
