# Supplementary material for: Engaging Parents in Technology-Assisted Interventions for Childhood Adversity: Systematic Review
Source: J Med Internet Res. 2024 Jan 19;26:e43994. doi: 10.2196/43994 (PMC10837762; doi:10.2196/43994)
Supplement: Multimedia Appendix 3 [file jmir_v26i1e43994_app3.docx]

**Appendix 3.** Additional references merged with main references

| Main reference | Additional reference(s) |
| --- | --- |
| Chu JTW, Wadham A, Jiang Y, Whittaker R, Stasiak K, Shepherd M, et al. Development of MyTeen Text Messaging Program to Support Parents of Adolescents: Qualitative Study. JMIR MHealth and UHealth. 2019;7(11):e15664. Doi:10.2196/15664 | Chu JTW, Whittaker R, Jiang Y, Wadham A, Stasiak K, Shepherd M, et al. Evaluation of MyTeen - a SMS-based mobile intervention for parents of adolescents: a randomised controlled trial protocol. BMC Public Health. 2018;18(1):1203. doi:10.1186/s12889-018-6132-z |
| Kaplan K, Solomon P, Salzer MS, Brusilovskiy E. Assessing an Internet-based parenting intervention for mothers with a serious mental illness: a randomized controlled trial. Psychiatric Rehabilitation Journal. 2014;37(3):222–31. Doi:10.1037/prj0000080 | Kaplan KM. Assessing the impact of an internet-based parenting intervention for mothers with psychiatric disabilities: A randomized controlled trial (Internet). Dissertation Abstracts International: Section B: The Sciences and Engineering. Vol.74(10-B(E)),2014, pp. No Pagination Specified.; 2014. Available from: https://ezproxy.lib.monash.edu.au/login?url=http://ovidsp.ovid.com/ovidweb.cgi?T=JS&CSC=Y&NEWS=N&PAGE=fulltext&D=psyc11&AN=2014-99080-332 |
| Sourander A, McGrath PJ, Ristkari T, Cunningham C, Huttunen J, Lingley-Pottie P, et al. Internet-Assisted Parent Training Intervention for Disruptive Behavior in 4-Year-Old Children: A Randomized Clinical Trial. JAMA Psychiatry. 2016;73(4):378–87. Doi:10.1001/jamapsychiatry.2015.3411 | McGrath PJ, Sourander A, Lingley-Pottie P, Ristkari T, Cunningham C, Huttunen J, et al. Remote population-based intervention for disruptive behavior at age four: study protocol for a randomized trial of Internet-assisted parent training (Strongest Families Finland-Canada). BMC Public Health. 2013;13(1):985–985. Doi:10.1186/1471-2458-13-985 |
| Baker S, Sanders MR, Turner KMT, Morawska A. A randomized controlled trial evaluating a low-intensity interactive online parenting intervention, Triple P Online Brief, with parents of children with early onset conduct problems. Behaviour Research & Therapy. 2017;91:78–90. Doi:10.1016/j.brat.2017.01.016 | Baker S, Sanders MR. Predictors of Program Use and Child and Parent Outcomes of A Brief Online Parenting Intervention. Child Psychiatry & Human Development. 2017;48(5):807–17. Doi:10.1007/s10578-016-0706-8 |
| Feil EG, Baggett K, Davis B, Landry S, Sheeber L, Leve C, et al. Randomized control trial of an internet-based parenting intervention for mothers of infants. Early Childhood Research Quarterly. 2020;50:36–44. Doi:10.1016/j.ecresq.2018.11.003 | Feil EG, Baggett K, Davis B, Leve C, Landry SH. Who Participates in an Internet-Based Research Program for Mothers of Infants? A Secondary Prevention Research Study Among Low-Income Families. Journal of Applied Research on Children. 2014;5(1):1–22. e-ISSN: 21555834 (no DOI available). |
| Wade SL, Cassedy AE, Shultz EL, Zang H, Zhang N, Kirkwood MW, et al. Randomized Clinical Trial of Online Parent Training for Behavior Problems After Early Brain Injury. Journal of the American Academy of Child & Adolescent Psychiatry. 2017;56(11):930-939.e2. doi:10.1016/j.jaac.2017.09.413 | Narad ME, Taylor HG, Yeates KO, Stancin T, Kirkwood MW, Wade SL. Internet-based Interacting Together Everyday, Recovery After Childhood TBI (I-InTERACT): Protocol for a multi-site randomized controlled trial of an internet-based parenting intervention. DIGITAL HEALTH. 2017 Jan;3:205520761771942. Doi:10.1177/2055207617719423 |
| Zlotnick C, Tzilos Wernette G, Raker CA. A randomized controlled trial of a computer-based brief intervention for victimized perinatal women seeking mental health treatment. Archives of Women’s Mental Health. 2019;22(3):315–25. Doi:10.1007/s00737-018-0895-1 | Nct. Internet-Based Intervention to Improve Mental Health Outcomes for Abused Women. https://clinicaltrials.gov/show/NCT01312103 (Internet). 2011; Available from: https://ezproxy.lib.monash.edu.au/login?url=http://ovidsp.ovid.com/ovidweb.cgi?T=JS&CSC=Y&NEWS=N&PAGE=fulltext&D=cctr&AN=CN-02035150  Hegarty K, Tarzia L, Murray E, Valpied J, Humphreys C, Taft A, et al. Protocol for a randomised controlled trial of a web-based healthy relationship tool and safety decision aid for women experiencing domestic violence (I-DECIDE). BMC Public Health. 2015;15:736. Doi:10.1186/s12889-015-2072-z |
| Fletcher R, Knight T, Macdonald JA, StGeorge J. Process evaluation of text-based support for fathers during the transition to fatherhood (SMS4dads): mechanisms of impact. BMC psychology. 2019;7(1):63. Doi:10.1186/s40359-019-0338-4 | Fletcher R, Kay-Lambkin F, May C, Oldmeadow C, Attia J, Leigh L. Supporting men through their transition to fatherhood with messages delivered to their smartphones: a feasibility study of SMS4dads. BMC Public Health. 2017 Dec;17(1):953. Doi:10.1186/s12889-017-4978-0 |
| Antonini TN, Raj SP, Oberjohn KS, Cassedy A, Makoroff KL, Fouladi M, et al. A pilot randomized trial of an online parenting skills program for pediatric traumatic brain injury: improvements in parenting and child behavior. Behavior Therapy. 2014;45(4):455–68. Doi:10.1016/j.beth.2014.02.003 | Raj SP, Antonini TN, Oberjohn KS, Cassedy A, Makoroff KL, Wade SL. Web-Based Parenting Skills Program for Pediatric Traumatic Brain Injury Reduces Psychological Distress Among Lower-Income Parents. Journal of Head Trauma Rehabilitation. 2015;30(5):347–56. Doi:10.1097/HTR.0000000000000052  Nct. Intervention for IPV Perinatal Women- RCT. https://clinicaltrials.gov/show/NCT02370394 (Internet). 2015; Available from: https://ezproxy.lib.monash.edu.au/login?url=http://ovidsp.ovid.com/ovidweb.cgi?T=JS&CSC=Y&NEWS=N&PAGE=fulltext&D=cctr&AN=CN-01480509 |
| Koziol-McLain J, Vandal AC, Wilson D, Nada-Raja S, Dobbs T, McLean C, et al. Efficacy of a Web-Based Safety Decision Aid for Women Experiencing Intimate Partner Violence: Randomized Controlled Trial. Journal of Medical Internet Research. 2018;19(12):e426. Doi:10.2196/jmir.8617 | Nct. Intervention for IPV Perinatal Women- RCT. https://clinicaltrials.gov/show/NCT02370394 (Internet). 2015; Available from: https://ezproxy.lib.monash.edu.au/login?url=http://ovidsp.ovid.com/ovidweb.cgi?T=JS&CSC=Y&NEWS=N&PAGE=fulltext&D=cctr&AN=CN-01480509  Koziol-McLain J, Vandal AC, Nada-Raja S, Wilson D, Glass NE, Eden KB, et al. A web-based intervention for abused women: the New Zealand isafe randomised controlled trial protocol. BMC Public Health. 2015;15:56. Doi:10.1186/s12889-015-1395-0 |
| Holden GW, Brown AS, Baldwin AS, Croft Caderao K. Research findings can change attitudes about corporal punishment. Child Abuse & Neglect. 2014 May;38(5):902–8. Doi:10.1016/j.chiabu.2013.10.013 | Holden GW, Brown AS. Brief online education. In: Gershoff ET, Lee SJ, editors. Ending the physical punishment of children: A guide for clinicians and practitioners (Internet). Washington: American Psychological Association; 2020 (cited 2022 Aug 15). p. 13–8. Available from: http://content.apa.org/books/16141-002. Doi:10.1037/0000162-002 |
| Enebrink P, Högström J, Forster M, Ghaderi A. Internet-based parent management training: A randomized controlled study. Behav Res Ther. 2012;50(4):240–9. Doi:10.1016/j.brat.2012.01.006 | Högström J, Enebrink P, Melin B, Ghaderi A. Eighteen-Month Follow-Up of Internet-Based Parent Management Training for Children with Conduct Problems and the Relation of Homework Compliance to Outcome. Child Psychiatry and Human Development. 2015;46(4):577–88. Doi:10.1007/s10578-014-0498-7 |
| Breitenstein SM, Fogg L, Ocampo EV, Acosta DI, Gross D. Parent Use and Efficacy of a Self-Administered, Tablet-Based Parent Training Intervention: A Randomized Controlled Trial. JMIR Mhealth Uhealth. 2016;4(2):e36–e36. Doi:10.2196/mhealth.5202 | Breitenstein SM, Brager J, Ocampo EV, Fogg L. Engagement and Adherence With *ez* PARENT, an mHealth Parent-Training Program Promoting Child Well-Being. Child Maltreat. 2017 Nov;22(4):295–304. Doi:10.1177/1077559517725402 |
| Yap MBH, Mahtani S, Rapee RM, Nicolas C, Lawrence KA, Mackinnon A, et al. A Tailored Web-Based Intervention to Improve Parenting Risk and Protective Factors for Adolescent Depression and Anxiety Problems: Postintervention Findings From a Randomized Controlled Trial. Journal of Medical Internet Research. 2018;20(1):e17. Doi:10.2196/jmir.9139 | Yap MBH, Cardamone-Breen MC, Rapee RM, Lawrence KA, Mackinnon AJ, Mahtani S, et al. Medium-Term Effects of a Tailored Web-Based Parenting Intervention to Reduce Adolescent Risk of Depression and Anxiety: 12-Month Findings From a Randomized Controlled Trial. Journal of Medical Internet Research. 2019;21(8):e13628. Doi:10.2196/13628 |
| Sim WH, Fernando LMN, Jorm AF, Rapee RM, Lawrence KA, Mackinnon AJ, et al. A tailored online intervention to improve parenting risk and protective factors for child anxiety and depression: Medium-term findings from a randomized controlled trial. Journal of Affective Disorders. 2020;277:814–24. Doi:10.1016/j.jad.2020.09.019 | Fernando LMN, Sim WH, Jorm AF, Rapee R, Lawrence KA, Yap MBH. Parenting Resilient Kids (PaRK), an online parenting program to prevent anxiety and depression problems in primary school-aged children: Study protocol for a randomised controlled trial. Trials. 2018 Dec;19(1):236. Doi:10.1186/s13063-018-2605-8 |
| Sanders MR, Dittman CK, Farruggia SP, Keown LJ. A comparison of online versus workbook delivery of a self-help positive parenting program. Journal of Primary Prevention. 2014;35(3):125–33. Doi:10.1007/s10935-014-0339-2 | Dittman CK, Farruggia SP, Palmer ML, Sanders MR, Keown LJ. Predicting success in an online parenting intervention: The role of child, parent, and family factors. Journal of Family Psychology. 2014 Apr;28(2):236–43. Doi:10.1037/a0035991 |
| Morgan AJ, Rapee RM, Salim A, Goharpey N, Tamir E, McLellan LF, et al. Internet-Delivered Parenting Program for Prevention and Early Intervention of Anxiety Problems in Young Children: Randomized Controlled Trial. Journal of the American Academy of Child & Adolescent Psychiatry. 2017;56(5):417-425.e1. doi:10.1016/j.jaac.2017.02.010 | Morgan AJ, Rapee RM, Bayer JK. Increasing response rates to follow-up questionnaires in health intervention research: Randomized controlled trial of a gift card prize incentive. Clinical Trials. 2017;14(4):381–6. Doi:10.1177/1740774517703320 |
| Fletcher R, Hammond C, Faulkner D, Turner N, Shipley L, Read D, et al. Stayin’ on Track: the feasibility of developing Internet and mobile phone-based resources to support young Aboriginal fathers. Aust J Prim Health. 2017;23(4):329. Doi:10.1071/PY16151 | Faulkner D, Hammond C, Nisbet L, Fletcher R. How do young aboriginal fathers in Australia ‘stay on track’? - perspectives on the support networks of aboriginal fathers. Journal of Family Studies. 2021;27(1):146–59. Doi:10.1080/13229400.2018.1537193 |
| Spence SH, Prosser SJ, March S, Donovan CL. Internet-delivered cognitive behavior therapy with minimal therapist support for anxious children and adolescents: predictors of response. Journal of Child Psychology & Psychiatry & Allied Disciplines. 2020;61(8):914–27. Doi:10.1111/jcpp.13257 | Spence SH, Donovan CL, March S, Gamble A, Anderson R, Prosser S, et al. Online CBT in the Treatment of Child and Adolescent Anxiety Disorders: Issues in the Development of BRAVE–ONLINE and Two Case Illustrations. Behav Cogn Psychother. 2008 Jul;36(4):411–30. Doi:10.1017/S135246580800444X |
| Piotrowska PJ, Tully LA, Collins DAJ, Sawrikar V, Hawes D, Kimonis ER, et al. ParentWorks: Evaluation of an Online, Father-Inclusive, Universal Parenting Intervention to Reduce Child Conduct Problems. Child Psychiatry & Human Development. 2020;51(4):503–13. Doi:10.1007/s10578-019-00934-0 | Tully LA, Piotrowska PJ, Collins DAJ, Mairet KS, Hawes DJ, Kimonis ER, et al. Study protocol: evaluation of an online, father-inclusive, universal parenting intervention to reduce child externalising behaviours and improve parenting practices. BMC Psychol. 2017;5(1):21–21. Doi:10.1186/s40359-017-0188-x  Dadds MR, Sicouri G, Piotrowska PJ, Collins DAJ, Hawes DJ, Moul C, et al. Keeping Parents Involved: Predicting Attrition in a Self-Directed, Online Program for Childhood Conduct Problems. Journal of Clinical Child & Adolescent Psychology. 2019;48(6):881–93. Doi:10.1080/15374416.2018.1485109 |
| Breitenstein SM, Fehrenbacher C, Holod AF, Schoeny ME. A Randomized Trial of Digitally Delivered, Self-Administered Parent Training in Primary Care: Effects on Parenting and Child Behavior. Journal of Pediatrics. 2021;231:207-214.e4. doi:10.1016/j.jpeds.2020.12.016 | Breitenstein SM, Schoeny M, Risser H, Johnson T. A study protocol testing the implementation, efficacy, and cost effectiveness of the ezParent program in pediatric primary care. Contemporary Clinical Trials. 2016 Sep;50:229–37. Doi:10.1016/j.cct.2016.08.017 |
| Metcalfe RE, Matulis JM, Cheng Y, Stormshak EA. Therapeutic alliance as a predictor of behavioral outcomes in a relationally focused, family-centered telehealth intervention. Journal of Marital & Family Therapy. 2021;47(2):473–84. Doi:10.1111/jmft.12517 | Danaher BG, Seeley JR, Stormshak EA, Tyler MS, Caruthers AS, Moore KJ, et al. The Family Check-Up Online Program for Parents of Middle School Students: Protocol for a Randomized Controlled Trial. JMIR Res Protoc. 2018;7(7):e11106–e11106. Doi:10.2196/11106  Stormshak EA, Matulis JM, Nash W, Cheng Y. The Family Check-Up Online: A Telehealth Model for Delivery of Parenting Skills to High-Risk Families With Opioid Use Histories. Frontiers in Psychology (Internet). 2021;12. Doi:10.3389/fpsyg.2021.695967 |
| Kavanagh DJ, Connolly J, Fisher J, Halford WK, Hamilton K, Hides L, et al. The Baby Steps Web Program for the Well-Being of New Parents: Randomized Controlled Trial. J Med Internet Res. 2021;23(11):e23659–e23659. Doi:10.2196/23659 | Kavanagh D, Sanders D. Does an online wellbeing program (Baby Steps) prevent perinatal distress in first-time fathers and mothers? Archives of Women’s Mental Health. 2020;23 (2):294–5. Doi:10.1007/s00737-019-00953-9 |
| Kirkman JJL, Hawes DJ, Dadds MR. An Open Trial for an E-Health Treatment for Child Behavior Disorders I: Social Acceptability, Engagement, and Therapeutic Process. Evidence-Based Practice in Child and Adolescent Mental Health. 2016 Oct;1(4):196–212. Doi:10.1080/23794925.2016.1230481 | Kirkman JJL, Hawes DJ, Dadds MR. An Open Trial for an E-Health Treatment for Child Behavior Disorders II: Outcomes and Clinical Implications. Evidence-Based Practice in Child and Adolescent Mental Health. 2016 Oct;1(4):213–29. Doi:10.1080/23794925.2016.1230482 |
