# Supplementary material for: Engaging Parents in Technology-Assisted Interventions for Childhood Adversity: Systematic Review
Source: J Med Internet Res. 2024 Jan 19;26:e43994. doi: 10.2196/43994 (PMC10837762; doi:10.2196/43994)
Supplement: Multimedia Appendix 4 [file jmir_v26i1e43994_app4.docx]

# Appendix 4

# Included Studies’ Characteristics, Engagement Strategies and Measures Used, Between-Group Engagement Outcomes and Between-Group ACE Outcomes

## **Table S1.** Summary of Characteristics in Included Studies

| Study (main reference) | Study design | Country | Mean age of youth | Parent gender | SEP | Level of prevention | Primary target ACE | Target recipient | Setting | Function of technology | Types of modes | Total no. of modes | Design framework | Program name |
| --- | --- | --- | --- | --- | --- | --- | --- | --- | --- | --- | --- | --- | --- | --- |
| Agazzi H, Hayford H, Thomas N, Ortiz C, Salinas-Miranda A. A (2021)* | Implementation study | USA | 3.17yrs | 86.8% female | Not reported | Universal | Maladaptive parenting | Parent | Home | Remote clinician contact | Videoconferencing  Telephone calls  Text messages | 3 | Not reported | iHOT-DOCTS (BPT) |
| Antonini TN, Raj SP, Oberjohn KS, Cassedy A, Makoroff KL, Fouladi M, et al. (2014)* | Pilot RCT | USA | 3-9 yrs | Not reported | Not reported | Indicated | Maladaptive parenting | Parent | Home (via health service or hospital) | Self-directed learning + remote clinician contact | Videoconferencing  Videos | 2 | Not reported | i-INTERACT |
| Baggett K, Davis B, Feil E, Sheeber L, Landry S, Leve C, et al. (2017) | RCT | USA | 3.5-7.5mo | 95.1% female | Low income and SEP | Selective | Child maltreatment | Parent + child | Home | Self-directed learning + remote clinician contact | Online modules  Telephone calls  Digital feedback | 3 | Not reported | Baby-Net |
| Baggett KM, Davis B, Feil EG, Sheeber LL, Landry SH, Carta JJ, et al. (2010) | RCT | USA | M=4.4mo | Not reported | Low income | Selective | Maladaptive parenting | Parent + child | Home | Self-directed learning + remote clinician contact + peer support | Digital feedback  Online modules  Telephone calls  Social media | 4 | Not reported | Infant-Net |
| Baggett KM, Davis B, Sheeber LB, Ammerman RT, Mosley EA, Miller K, et al. (2020) | Cross-sectional analytic study of an RCT | USA | 5.16 (2.82)yrs | 100% female | Low SEP (income and from non-dominant cultural background) | Indicated | Maladaptive parenting | Parent | Home | Self-directed learning + remote clinician contact | Online modules | 1 | Not reported | Mom and Baby Net |
| Baker M, Biringen Z, Meyer-Parsons B, Schneider A. (2020) | Pilot study (using randomised allocation and experimental design) | USA | 3.5yrs | 83% women | High SEP | Selective | Maladaptive parenting | Parent + child | Home | Self-directed learning + remote clinician contact | Videoconferencing  Website | 2 | Not reported | Emotional Attachment and Emotional Availability (EA2) |
| Baker S, Sanders MR, Turner KMT, Morawska A. A (2017) | RCT | Australia | 4.57yrs | Not reported | Not reported | Selective | Maladaptive parenting | Parent | Home | Self-directed learning | Online modules  Email  Telephone calls | 3 | Not reported | Triple P Online - Brief |
| Becher EH, Cronin S, McCann E, Olson KA, Powell S, Marczak MS. (2015) | Evaluation study | USA | Not reported | 100%female | Not reported | Selective | Interparental conflict | Parent | Home | Self-directed learning | Online modules | 1 | Not reported | Parents Forever |
| Bloom TL, Glass NE, Case J, Wright C, Nolte K, Parsons L. (2014) | Feasibility study | USA | Not reported (perinatal) | 77.36% female | Low SEP/ vulnerable population | Indicated | Interparental conflict | Parent | Participants' choice | Service enhancement | Website | 1 | Not reported | Not reported |
| Bodenmann G, Hilpert P, Nussbeck FW, Bradbury TN. (2014) | RCT | Switzerland | Not reported | 54.71% female | Not reported | Universal | Interparental conflict | Parent+others | Home | Self-directed learning + remote clinician contact | Videos  Telephone calls | 2 | Not reported | Couple Coping Enhancement Training-DVD |
| Boekhorst MG, Hulsbosch LP, Nyklicek I, Spek V, Kastelein A, Bogels S, et al. (2021) | Evaluation study (as part of an RCT) | The Netherlands | 3.5(.23)yrs | Not reported | High SEP | Selective | Maladaptive parenting | Parent | Home | Self-directed learning | Online modules | 1 | Not reported | Mindful Parenting Training |
| Breaux R, Shroff DM, Cash AR, Swanson CS, Carlton C, Bertollo JR, et al. (2021)* | Evaluation study | USA | 12.79(1.1)yrs | 100% female | Not reported | Indicated | Maladaptive parenting | Parent | Home (via health service or hospital) | Remote clinician contact | Videoconferencing | 1 | Not reported | RELAX |
| Breitenstein SM, Fehrenbacher C, Holod AF, Schoeny ME. (2021) | RCT | USA | M = 2.2 (7.9)yrs | Not reported | Low SEP | Universal | Maladaptive parenting | Parent + child | Home | Self-directed learning | Online modules  Text messages | 2 | Not reported | ezPARENT |
| Breitenstein SM, Fogg L, Ocampo EV, Acosta DI, Gross D. (2016)* | RCT | USA | M = 4 (approx) yrs | 50/50 | Low income | Selective | Maladaptive parenting | Parent + child | Home (via health service or hospital) | Self-directed learning | Online modules  Text messages | 2 | Not reported | ezParent (adapted from Chicago Parent Program) |
| Breitenstein SM, Shane J, Julion W, Gross D. Developing the eCPP: (2015) | Development paper | USA | Not reported | 91% female | Low SEP | Selective | Maladaptive parenting | Parent and child | Community setting | Self-directed learning | Online modules  Text messages | 2 | Iterative design | eCPP (Chicago Parent Program) |
| Brophy-Herb HE, Moyses K, Shrier C, Rymanowicz K, Pilkenton A, Dalimonte-Merckling D, et al. (2021) | Analytical cross-sectional | USA | 22.95mo(11.17) | 89.9% female | Low income | Universal | Maladaptive parenting | Parent + child | Home | Self-directed learning + remote clinician contact | Online modules | 1 | Not reported | BEES (Building Early Emotional Skills) |
| Cardamone-Breen MC, Jorm AF, Lawrence KA, Rapee RM, Mackinnon AJ, Yap MBH. (2018) | RCT | Australia | 13.6 (1.03)yrs | 97.7% female | Not reported | Universal | Maladaptive parenting | Parent | Home | Self-directed learning | Website | 1 | User-centred design | Partners in Parenting (PiP) - Single Session |
| Cefai J, Smith D, Pushak RE. (2010)* | RCT | Australia | 11.9(1.8)yrs | 86.4% female | Not reported | Universal | Maladaptive parenting | Parent and child | Participants' choice | Self-directed learning | Online modules | 1 | Not reported | Parenting Wisely |
| Choi H, Kim S, Ko H, Kim Y, Park CG. (2016) | Quasi-experimental RCT | Korea | M not reported (Range:11-16)yrs | 88.5% female | Not reported | Universal | Maladaptive parenting | Parent + child | Home | Self-directed learning (?) | Online modules  Telephone calls  Text messages | 3 | Not reported | Stepping Stone |
| Chu JTW, Wadham A, Jiang Y, Whittaker R, Stasiak K, Shepherd M, et al. (2019) | Qualitative study | New Zealand | Not reported (range: 10-15yrs) | 100% female | Not reported (Hard to reach population) | Universal | Maladaptive parenting | Parent | Home | Self-directed learning | Text messages | 1 | Not reported | MyTeen |
| Comer JS, Furr JM, Miguel EM, Cooper-Vince CE, Carpenter AL, Elkins RM, et al. (2017)* | RCT | USA | 3.8(8)yrs | Not reported | Not reported | Indicated | Maladaptive parenting | Parent | Home | Remote clinician contact | Videoconferencing | 1 | Not reported | Internet Parent Child Interaction Therapy (I-PCIT) |
| Cotter KL, Bacallao M, Smokowski PR, Robertson CIB. (2013) | Quasi-experimental study | USA | Not reported (eligibility 11-15yo) | 100% male | Low SEP | Selective | Maladaptive parenting | Parent | Home | Self-directed learning | Online modules | 1 | Not reported | Parenting Wisely |
| Czymoniewicz-Klippel M, Chesnut R, DiNallo J, Perkins D. (2019) | Feasibility study | USA | 7.09(1.69)yrs | 99% female | Not reported | Universal | Maladaptive parenting | Parent | Home | Self-directed learning | Online modules  Email | 2 | Not reported | Grow Online |
| Dadds MR, Thai C, Mendoza Diaz A, Broderick J, Moul C, Tully LA, et al. (2019)* | RCT | Australia | 6.79 (1.68)yrs | 85.5% female | Low SEP (rurality) | Indicated | Maladaptive parenting | Parent + child | Home | Self-directed learning + remote clinician contact | Videos  Videoconferencing | 2 | Not reported | AccessEI |
| Day JJ, Sanders MR. (2018)* | RCT | Australia | 2-8yo (m 3.5) | 86% female | Not reported | Selective | Maladaptive parenting | Parent | Home | Self-directed learning + remote clinician contact | Online modules  Telephone calls  Text messages  Email | 4 | Not reported | Triple P Online (TPOL) |
| DeGarmo DS, Jones JA. (2019) | RCT | USA | M = 7.88yrs | 100% female | Not reported | Selective | Maladaptive parenting | Parent | Home | Self-directed learning | Online modules  Email | 2 | Not reported | Fathering Through Change (FTC) |
| Donovan CL, March S. (2014) | RCT | Australia | M = 4.08 (.76)yrs | Not reported | Not reported (average-high average) | Indicated | Maladaptive parenting | Parent | Home | Self-directed learning + remote clinician contact | Online modules  Telephone calls  Email | 3 | Not reported | Adapted from BRAVE-ONLINE |
| Doss BD, Roddy MK, Llabre MM, Georgia Salivar E, Jensen-Doss A. (2020) | RCT (Secondary data analysis of both groups) | USA | M 8.21years-old, SD 4.30 | 94 mothers, 75 fathers | Not reported (Average) | Indicated | Interparental conflict | Parent and child | Home | Self-directed learning + remote clinician contact | Online modules  Participants' choice (telephone or videoconferencing)  Social media | 3 | Not reported | OurRelationship |
| Ehrensaft MK, Knous-Westfall HM, Alonso TL. (2016) | RCT | USA | Not reported | 80.4% female | At risk group | Selective | Maladaptive parenting | Parent | Home | Self-directed learning | Online modules  Telephone calls | 2 | Not reported | Triple P Online (TPOL) |
| Enebrink P, Högström J, Forster M, Ghaderi A. (2012). | RCT | Sweden | 3-12yo (mean 6.83) | Female (n = 19) and male (n = 11) | High SEP | Indicated | Maladaptive parenting | Parent | Home | Self-directed learning + remote clinician contact | Online modules | 1 | Not reported | Parent Management Training (PMT) |
| Epstein M, Oesterle S, Haggerty KP. (2019) | RCT | USA | Not reported (range: 11-13) | 100% female | Not reported | Universal | Maladaptive parenting | Parent | Home | Self-directed learning + peer support | Social media  Videos | 2 | Not reported | Parenting in the Middle School Years (PIMSY) |
| Farris JR, Bert SSC, Nicholson JS, Glass K, Borkowski JG. (2013) | RCT | USA | M not reported (2-3yrs) | 86.2% female | Not reported | Universal | Maladaptive parenting | Parent + child | Home | Self-directed learning | Videos  Email | 2 | Not reported | Adventures in Parenting |
| Fletcher R, Hammond C, Faulkner D, Turner N, Shipley L, Read D, et al. (2017) | Feasibility study | Australia | Not reported ('young child') | 50% female | 25% from small rural town | Universal | Maladaptive parenting | Service providers (delivering parenting program) | Home | Self-directed learning + remote clinician contact | Text messages  Website  Videos  Telephone calls | 4 | User-involved design | SMS4DADS |
| Feil EG, Baggett K, Davis B, Landry S, Sheeber L, Leve C, et al. (2020) | RCT | USA | M = 4.41, sd 2.05 (3.5-7.5months) | 44% female | Low income and SEP | Selective | Maladaptive parenting | Parent | Home | Self-directed learning + remote clinician contact | Online modules  Telephone calls  Digital feedback | 3 | Not reported | ePALS |
| Ferraro AJ, Oehme K, Bruker M, Arpan L, Opel A. (2020) | Pilot study | USA | Not reported | 100%female | High SEP | Selective | Interparental conflict | Parent | Home | Self-directed learning | Online modules | 1 | Not reported | SuccessfulCoparenting After Divorce |
| Fleming GE, Kohlhoff J, Morgan S, Turnell A, Maiuolo M, Kimonis ER. (2021) | Effectiveness trial | Australia | 3.02 (0.73)yrs | Not reported | Not reported | Indicated | Maladaptive parenting | Parents | Home | Remote clinician contact | Videoconferencing | 1 | Not reported | Internet Parent Child Interaction Therapy (I-PCIT) |
| Fletcher R, Campbell L, Sved Williams A, Rawlinson C, Dye J, Baldwin A, et al. (2019) | Descriptive paper | Australia | Infant | 62% female | Not reported | Indicated | Maladaptive parenting | Parent | Home | Self-directed learning | Text messages | 1 | Other | SMS4PerinatalParents |
| Fletcher R, Knight T, Macdonald JA, StGeorge J. (2019) | Process evaluation study | Australia | Not reported | 50% male/female | Average | Selective | Maladaptive parenting | Parent | Home | Self-directed learning | Text messages | 1 | Iterative design | SMS4DADS |
| Fletcher R, May C, Wroe J, Hall P, Cooke D, Rawlinson C, et al. (2016) | Development paper | Australia | 6.8 months | Mothers n = 56; fathers n = 46; unknown n = 4) | High SEP | Selective | Maladaptive parenting | Parent | Home | Self-directed learning | Text messages | 1 | Iterative design | SMS4Dads |
| Flujas-Contreras JM, Garcia-Palacios A, Gomez I. (2021) | Single arm non-randomised controlled trial | Spain | M = 7.84; SD = 3.48 | Not reported | Not reported | Universal | Maladaptive parenting | Parent + child | Home | Self-directed learning | Online modules  Emails | 2 | Not reported | Parenting Forest |
| Ford-Gilboe M, Varcoe C, Scott-Storey K, Perrin N, Wuest J, Wathen CN, et al. (2020) | RCT | Canada | Not reported | 100% female | Low SEP | Indicated | Interparental conflict | Parent+others | Home | Self-management | Online modules | 1 | Not reported | iCan Plan 4 Safety |
| Fox J, Archard PJ. (2018) | Development paper | UK | N/A | Female | Not reported | Selective | Maladaptive parenting | Social workers | Home (via community or social service) | Self-directed learning | Online modules | 1 | Other | Not reported |
| Franke N, Keown LJ, Sanders MR. (2020) | RCT | New Zealand | m=4.0yrs | 84.3% female | Average | Selective | Maladaptive parenting | Parent | Home | Self-directed learning | Online modules  Telephone calls | 2 | Not reported | Triple P Online (TPOL) |
| Fulgoni CMF, Melvin GA, Jorm AF, Lawrence KA, Yap MBH. (2019) | Feasibility study | Australia | Not reported | Not reported | Not reported | Indicated | Maladaptive parenting | Parent | Home | Self-directed learning + remote clinician contact | Online modules  Videoconferencing  Email | 3 | User-involved design | Therapist-Assisted Online Parenting Strategies |
| Gelatt VA, Adler-Baeder F, Seeley JR. (2010) | Evaluation study (using randomised allocation and experimental design) | USA | Not reported (11-15yrs) | 87% female | Average-to high | Selective | Maladaptive parenting + interparental conflict | Parent | Home | Self-directed learning | Online modules  Email | 2 | Other | Parenting Toolkit: Skills for Stepfamilies |
| Ghaderi A, Kadesjo C, Bjornsdotter A, Enebrink P. (2018) | RCT | Sweden | Not reported (range: 10-13yrs) | 92% female | Average | Indicated | Maladaptive parenting | Parent | Home | Self-directed learning | Online modules | 1 | Not reported | iComet |
| Gulirmak K, Orak OS. (2020) | Quasi-experimental RCT | Turkey | M not reported (<19yrs) | Not reported | Not reported | Universal | Child maltreatment | Parent | Home (via health service or hospital) | Self-directed learning | Online modules  Videos  Email | 3 | Not reported | Not reported |
| H. Mohammadinasab, M. Mazaheri, M. Reazaeizade, M. Heydari. (2020) | Case study | Iran | 13yrs | Not reoprted | High SEP | Universal | Maladaptive parenting | Parent and child | Home | Self-directed learning | Videos  Text messages | 2 | Other | Not reported |
| Hamil J, Gier E, Garfield CF, Tandon D. (2021) | Pilot study | USA | Not reported (range: prenatal - 18 months) | 92.2% female | High vulnerability (home visitation context) | Selective | Maladaptive parenting + interparental conflict | Parent | Home | Self-directed learning | Text messages | 1 | Not reported | Fathers and Babies (FAB) |
| Hegarty K, Tarzia L, Valpied J, Murray E, Humphreys C, Taft A, et al. (2019) | RCT | Australia | Not reported | 85.4% female | Low SEP | Indicated | Interparental conflict | Parent + child | Home | Self-directed learning + action planning | Online modules | 1 | Not reported | I-DECIDE |
| Hemdi A, Daley D. (2017) | RCT | Saudi Arabia | 63.18 (13.68) months | 94.1% female | High SEP | Indicated | Maladaptive parenting | Parent | Home | Self-directed learning + Remote clinician contact | Text messages | 1 | Not reported | Not reported |
| Hinton S, Sheffield J, Sanders MR, Sofronoff K. (2017) | RCT | Australia | 6.33 (2.43)yrs | Female | Not reported | Indicated | Maladaptive parenting | Parent | Home | Self-directed learning + remote clinician contact | Online modules  Telephone calls  Email  Social media | 4 | Not reported | Triple P Online – Disability (TPOL-D) |
| Holden GW, Brown AS, Baldwin AS, Croft Caderao K. (2014) | Exploratory study (using randomised allocation and experimental design) | USA | Not reported | 59% female | Not reported | Universal | Child maltreatment | Parent | Home | Self-directed learning | Website | 1 | Not reported | Not reported |
| Hudson DB, Campbell-Grossman C, Hertzog M. (2012) | Longitudinal experimental design | USA | 1-6months | Mothers | Low SEP | Selective | Maladaptive parenting + Child maltreatment | Parent | Home (via health service or hospital) | Self-directed learning + remote clinician contact | Website  Social media  Email | 3 | Not reported | New Mothers Network (NMN) |
| Huebner DM, Rullo JE, Thoma BC, McGarrity LA, Mackenzie J. (2013) | Pilot study | USA | 18.6yrs | 100% female | Not reported | Universal | Maladaptive parenting | Parent | Home | Education entertainment | Videos | 1 | Not reported | Lead with Love |
| Irvine AB, Gelatt VA, Hammond M, Seeley JR. (2015) | RCT | USA | 13.1 yrs (SD=1.4). | Not reported | Low income | Selective | Maladaptive parenting | Parent + child | Community setting | Self-directed learning | Online modules | 1 | Other | Parenting Toolkit |
| James Riegler L, Raj SP, Moscato EL, Narad ME, Kincaid A, Wade SL. (2020) | Single arm pilot trial | USA | Not reported (range 3-9) | Not reported | Low income | Selective | Maladaptive parenting | Parent | Home | Self-directed learning + remote clinician contact | Online modules  Videoconferencing | 2 | Not reported | Online Parenting Pro-Tips (OPPT) |
| Jones DJ, Forehand R, Cuellar J, Parent J, Honeycutt A, Khavjou O, et al. (2014)* | Pilot RCT | USA | 5.57(1.27)yrs | 100% female | Low income | Indicated | Maladaptive parenting | Parent and child | Home (via health service or hospital) | Self-directed learning + remote clinician contact | Videoconferencing  Videos  Digital feedback  Text messages | 4 | Iterative design | TE-HNC (Technology-enhanced Helping the Non Compliant Child) |
| Jones DJ, Loiselle R, Zachary C, Georgeson AR, Highlander A, Turner P, et al. (2021)* | Evaluation study (using randomised allocation and experimental design) | USA | 3.95(0.79)yrs | Male | Low income | Indicated | Maladaptive parenting | Parent | Home (via health service or hospital) | Self-directed learning + remote clinician contact | Videoconferencing  Computer/Tablet/Phone applications (Apps)  Telephone calls  Videos  Text messages | 5 | Not reported | TE-HNC (Technology-enhanced Helping the Non Compliant Child) |
| Jones S, Calam R, Sanders M, Diggle PJ, Dempsey R, Sadhnani V. (2014) | Pilot RCT | UK | 6.13yo (completers) and 8.00yo (non-completers) | Not reported | Low SEP | Selective | Maladaptive parenting | Parent + child | Home | Self-directed learning | Online modules | 1 | Not reported | Triple P Parenting Media |
| Kaplan K, Solomon P, Salzer MS, Brusilovskiy E. (2014) | RCT | USA | Not reported | 58% female | Vulnerable population? | Indicated | Maladaptive parenting | Parent | Home | Self-directed learning | Online modules  Social media | 2 | Not reported | Not reported |
| Kavanagh DJ, Connolly J, Fisher J, Halford WK, Hamilton K, Hides L, et al. (2021)* | RCT | Australia | Infants/Neonates | 85.71% female | Not reported | Universal | Interparental conflict | Parent | Home (via health service or hospital) | Self-directed learning | Online modules  Text messages | 2 | Not reported | Baby Steps Wellbeing |
| Khor SPH, Fulgoni CM, Lewis D, Melvin GA, Jorm AF, Lawrence K, et al. (2021) | Single arm trial | Australia | 15.02 (SD 1.56) yrs | 80% female | Average | Indicated | Maladaptive parenting | Parent and child | Home | Self-directed learning + remote clinician contact | Online modules  Videoconferencing  Email | 3 | User-centred design | Therapist Assisted Online Parenting Strategies (TOPS) |
| Kirkman JJL, Hawes DJ, Dadds MR. (2016)* | Non-randomised trial | Australia | 7.76(2.68)yrs | Not reported | Low SEP (low SEIFA rank) | Indicated | Maladaptive parenting | Women | Home | Self-directed learning + remote clinician contact | Videos  Videoconferencing | 2 | Not reported | Integrated Family Intervention for Child Conduct Problems (BPT) |
| Kopystynska O, Turner JJ, Schramm DG, Higginbotham B. (2020) | Cross-sectional study | USA | Not reported | 100% male | Not reported | Selective | Interparental conflict | Parent | Home (via community or social service) | Self-directed learning | Online modules | 1 | Not reported | Divorce Education |
| Koziol-McLain J, Vandal AC, Wilson D, Nada-Raja S, Dobbs T, McLean C, et al. (2018) | RCT | New Zealand | Not reported | Women | Low SEP | Indicated | Interparental conflict | Parent | Home | Self-management | Online modules  Email  Telephone calls | 3 | Not reported | iSafe |
| Lawrence KA, Cardamone-Breen MC, Green J, Yap MBH, Jorm AF, Rapee RM. (2017) | N/A | Australia | M not reported (range 11-18yrs) | 79.1% female | Highly educated | Universal | Maladaptive parenting | Parent | Home | Self-directed learning | Online modules | 1 | User-centred design | Partners in Parenting (PiP) |
| Loew B, Rhoades G, Markman H, Stanley S, Pacifici C, White L, et al. (2012) | Pilot RCT | USA | Not reported | Not reported | Not reported | Selective | Interparental conflict | Parent | Home (via community or social service) | Self-directed learning | Online modules | 1 | Not reported | Prevention and Relationship Education Program (PREP) |
| Love SM, Sanders MR, Metzler CW, Prinz RJ, Kast EZ. (2013) | Cross-sectional study, qualitative study | USA | Not reported | 94.4% female | Low SEP | Selective | Child maltreatment | Parent | Home | Self-directed learning + peer support | Videos  Website | 3 | Not reported | Triple P Online (TPOL) |
| Love SM, Sanders MR, Turner KMT, Maurange M, Knott T, Prinz R, et al. (2016) | Feasibility study | USA | M not reported. (2yo-12yo) | 50% male/female | Low SEP, high vulnerability | Indicated | Maladaptive parenting | Parent | Participants' choice | Self-directed learning + peer support | Online modules  Social media | 2 | Community-based involvement | Triple P Online Community (TPOC) |
| Mast JE, Antonini TN, Raj SP, Oberjohn KS, Cassedy A, Makoroff KL, et al. (2014) | Pilot study (using randomised allocation and experimental design) | USA | Not reported (range 3-9yo) | 100% female | Not reported | Indicated | Maladaptive parenting + Child maltreatment | Parent | Home (via health service or hospital) | Self-directed learning + remote clinician contact | Videoconferencing  Online modules  Participants' choice (telephone or email) | 3 | Not reported | I-InTERACT |
| May CD, Fletcher R. (2019) | Development paper | Australia | N/A | 100% female | N/A | Selective | Maladaptive parenting | Stakeholders | Home | Self-directed learning | Text messages | 1 | Not reported | SMS4Dads |
| May CD, St George JM, Lane S. (2021) | Evaluation study | Australia | M(ode) = 6 | 90% female | Average | Selective | Maladaptive parenting | Parents | Home | Self-directed learning | Text messages | 1 | Iterative design | SMS4DADS |
| Mello MJ, Bromberg JR, Baird J, Wills H, Gaines BA, Lapidus G, et al. (2019) | Feasibility study (using randomised allocation and experimental design) | USA | 15.8 yrs | 91.3% mothers | Not reported | Indicated | Maladaptive parenting | Parent and child | Home (via health service or hospital) | Self-directed learning | Online modules  Text messages  Social media | 3 | Not reported | Parenting Wisely |
| Metcalfe RE, Matulis JM, Cheng Y, Stormshak EA. (2021) | Evaluation study | USA | 11-13yrs (7.8% of children aged 11, 39.6% aged 12, and 22.5% aged 13) | 97.95% female | Low SEP | Universal | Maladaptive parenting | Parent | Home (via community or social service) | Self-directed learning | Online modules  Text messages  Telephone calls | 3 | Iterative design | Family Check Up Online |
| Metzler CW, Sanders MR, Rusby JC, Crowley RN. (2012) | Cross-sectional study | USA | 3-6yrs | 79% female | Average | Universal | Maladaptive parenting | Parent | Home | Self-directed learning | Videos | 1 | Not reported | Triple P Parenting Media |
| Morawska A, Tometzki H, Sanders MR. (2014) | RCT | Australia | 5.9 (2.27) yrs | 100% female | Not reported | Universal | Maladaptive parenting | Parent | Home | Self-directed learning | Podcasts  Email | 2 | Not reported | Triple P Podcast series |
| Morgan AJ, Rapee RM, Bayer JK. (2016)* | Pilot RCT | Australia | M = 4.7 yrs, SD = 0.8 | 94.6% female | Not reported | Selective | Maladaptive parenting | Parent | Home | Self-directed learning + remote clinician contact | Online modules  Telephone calls  Email | 3 | User-centred design | Cool Little Kids Online |
| Morgan AJ, Rapee RM, Salim A, Goharpey N, Tamir E, McLellan LF, et al. (2017) | RCT | Australia | M = 4.8 yrs (1.0) | 85% female | Not reported | Selective | Maladaptive parenting | Parent | Home | Self-directed learning | Online modules  Telephone calls  Email  Text messages | 4 | User-centred design | Cool Little Kids |
| Murry VM, Berkel C, Liu N. (2018)* | RCT | USA | 11.4yrs | 100% male | Low SEP | Selective | Maladaptive parenting | Parent and child | Community setting | Self-directed learning + Remote clinician contact | Online modules | 1 | User-centred design | Pathways for African American Success (PAAS) |
| Narad ME, Minich N, Taylor HG, Kirkwood MW, Brown TM, Stancin T, et al. (2015) | RCT | USA | 12-17yo | 86% female | Not reported | Indicated | Maladaptive parenting | Parent + child | Home | Self-directed learning + remote clinician contact | Online modules  Videoconferencing | 2 | Not reported | Counselor-Assisted Problem-Solving (CAPS) |
| Nickerson AB, Livingston JA, Kamper-DeMarco K. (2018) | RCT | USA | 6.62(2.55) | 50%female | Not reported | Universal | Child maltreatment | Parent | Home | Self-directed learning | Videos  Email  Text messages | 3 | Not reported | Second Step |
| O’Shea A, Kaplan K, Solomon P, Salzer MS. (2019) | RCT | USA | Not reported | Not reported | Vulnerable population | Indicated | Maladaptive parenting | Parent | Home | Self-directed learning | Online modules  Social media | 2 | Not reported | Not reported |
| Olthuis JV, McGrath PJ, Cunningham CE, Boyle MH, Lingley-Pottie P, Reid GJ, et al. (2018) | RCT | Canada | 8.9 yrs | 100% female | Not reported (range of low and higher income earners) | Indicated | Maladaptive parenting | Parent | Home (via health service or hospital) | Self-directed learning + remote clinician contact | Telephone calls  Videos | 2 | Not reported | Strongest Families |
| Owen DA, Hutchings J. (2017) | Feasibility study | Wales | Not reported (range 3-8 yrs) | 59.9% male | Not reported (looks average-high) | Universal | Maladaptive parenting | Parent | Home | Self-directed learning | Online modules | 1 | Not reported | COPING Online |
| Perrino T, Estrada Y, Huang S, St George S, Pantin H, Cano MA, et al. (2018) | RCT (Secondary data analysis of intervention group only) | USA | 14.1 (0.7) yrs | 100% female | Low income | Indicated | Maladaptive parenting | Parent + child | Home (via community or social service) | Self-directed learning + remote clinician contact | Videos  Videoconferencing | 2 | Not reported | eHealth Familias Unidas |
| Piotrowska PJ, Tully LA, Collins DAJ, Sawrikar V, Hawes D, Kimonis ER, et al. (2020) | Single arm trial | Australia | 5.88 (3.49) yrs | 100% femle | Not reported (Average-high) | Universal | Maladaptive parenting + interparental conflict | Parent | Home | Self-directed learning | Online modules  Email | 2 | Not reported | ParentWorks |
| Porzig-Drummond R, Stevenson RJ, Stevenson C. (2015) | RCT | Australia | 5.21 (1.74) yrs | 56% female | Above average SEP | Indicated | Maladaptive parenting | Parent and child | Home | Self-directed learning | Videos  Email | 2 | Not reported | 1-2-3 Magic |
| Potharst ES, Boekhorst MGBM, Cuijlits I, van Broekhoven KEM, Jacobs A, Spek V, et al. (2019) | RCT | Netherlands | M = 3.5 (0.23) yrs | Mothers | High SEP | Universal | Maladaptive parenting | Parents | Home | Self-directed learning | Online modules | 1 | Not reported | Mindful Parenting |
| Prinz RJ, Metzler CW, Sanders MR, Rusby JC, Cai C. (2021) | RCT | USA | 3-7yo (M= 57.4mo (15.2) | Not reported | Not reported | Selective | Maladaptive parenting | Parent | Home | Self-directed learning | Online modules  Telephone calls | 2 | Not reported | Triple P Online (TPOL) |
| Rabbitt SM, Carrubba E, Lecza B, McWhinney E, Pope J, Kazdin AE. (2016)* | RCT | USA | 6-13yo (M = 8.48, SD = 1.87) | 71% female | Not reported | Indicated | Maladaptive parenting | Parent + child | Home (via health service or hospital) | Self-directed learning + remote clinician contact | Videoconferencing  Participants' choice (telephone or email) | 2 | Not reported | Parent Management Training (PMT) |
| Ragavan MI, Ferre V, Bair-Merritt M. (2020) | Formative evaluation study | USA | N/A | 76% female | Not reported | Indicated | Interparental conflict | Parent* | Health setting | Self-directed learning | Computer/Tablet/Phone applications (Apps) | 1 | Not reported | THRIVE |
| Raj SP, Shultz EL, Zang H, Zhang N, Kirkwood MW, Taylor HG, et al. (2018) | RCT | USA | 6.29 (1.96) yrs | 100% female | Average | Indicated | Maladaptive parenting | Parent + child | Home (via health service or hospital) | Self-directed learning + remote clinician contact | Online modules  Videoconferencing | 2 | Not reported | i-INTERACT |
| Razuri EB, Hiles Howard AR, Parris SR, Call CD, DeLuna JH, Hall JS, et al. (2016) | RCT | USA | 8.18 (2.13) yrs | 100% female | Not reported | Selective | Maladaptive parenting | Parent | Home | Self-directed learning | Online modules | 1 | Not reported | Trust-Based Relational Intervention |
| Richardson HL. (2021) | RCT | USA | Not reported (range 1-5) | Not reported | Low income | Universal | Maladaptive parenting | Parent | Health setting | Self-directed learning | Online modules | 1 | Not reported | Play Nicely |
| Ristkari T, Kurki M, Suominen A, Gilbert S, Sinokki A, Kinnunen M, et al. (2019) | Comparison study | Finland | 4yo | 93.2% female | Not reported | Selective | Maladaptive parenting | Parent | Home (via health service or hospital) | Self-directed learning + remote clinician contact | Online modules  Telephone calls  Email | 3 | Other | Strongest Families Smart Website (SFSW) |
| Rizzo CJ, Houck C, Barker D, Collibee C, Hood E, Bala K. (2020) | RCT | USA | M = 13(.7) yrs | Not reported | Low income | Universal | Maladaptive parenting | Parent + child | Participants' choice | Self-directed learning | Online modules | 1 | Not reported | Project STRONG |
| Rudd BN, Holtzworth-Munroe A, Reyome JG, Applegate AG, D’Onofrio BM. (2015) | RCT | USA | 36.16mo | Not reported | Low income | Selective | Interparental conflict | Parent | Participants' choice | Self-directed learning | Online modules | 1 | Not reported | Proud to Parent |
| Russell BS, Maksut JL, Lincoln CR, Leland AJ. (2016) | Cross-sectional study | USA | Not reported | 50% male/female | High SEP | Universal | Maladaptive parenting | Parent | Other (workplace) | Group learning | Videos | 1 | Not reported | Not reported |
| Sanders MR, Baker S, Turner KMT. (2012) | RCT | Australia | M = 4.7 yrs (2-9- yrs) | 95.5% female | Not reported | Selective | Maladaptive parenting + interparental conflict | Parent | Home | Self-directed learning | Online modules  Podcasts | 2 | Not reported | Triple P Online (TPOL) |
| Sanders MR, Dittman CK, Farruggia SP, Keown LJ. (2014)* | RCT | New Zealand | M = 5.63 yrs (3 and 8 yrs) | 100% male | Average | Selective | Maladaptive parenting | Parent | Home | Self-directed learning | Online modules  Podcasts | 2 | Not reported | Triple P Online (TPOL) |
| Sawyer A, Kaim A, Le HN, McDonald D, Mittinty M, Lynch J, et al. (2019) | RCT | Australia | Not reported (perinatal) | 86.3% female | Not reported (appears high) | Indicated | Maladaptive parenting | Parent | Home (via health service or hospital) | Self-directed learning + remote clinician contact | Social media  Computer/Tablet/Phone applications (Apps) | 2 | Iterative design | eMUMS |
| Scholer SJ, Hudnut-Beumler J, Dietrich MS. (2010) | RCT | USA | M=2.4, SD 1.2 (1-5 years) | 91% female | Low income | Universal | Child maltreatment | Parent | Health setting | Service enhancement | Videos | 1 | Not reported | Play Nicely |
| Schramm DG, McCaulley G. (2012)* | Analytical cross-sectional | USA | Not reported | Not specified (mostly men) | Not reported | Selective | Interparental conflict | Parent | Home | Self-directed learning | Videos | 1 | Not reported | Focus on Kids (Online) |
| Self-Brown S, Cowart-Osborne M, Baker E, Thomas A, Boyd C, Chege E, et al. (2015) | Pilot study | USA | Not reported (2 and 5yrs) | 100% male | Low SEP | Selective | Child maltreatment | Parent + child | Home (via health service or hospital) | Service enhancement | Online modules | 1 | Not reported | Dad2K |
| Self-Brown S, Reuben K, Perry EW, Bullinger LR, Osborne MC, Bielecki J, et al. (2020) | Mixed methods study | USA, Canada and Australia | Not reported | 90% female | Not reported (vulnerable/high risk given program context) | Indicated | Child maltreatment | Parent | Home (via community or social service) | Remote clinician contact | Videoconferencing  Text messages | 2 | Not reported | SafeCare (virtual) |
| Self-Brown SR, C. Osborne M, Rostad W, Feil E. (2017) | Implementation study | USA | Not reported | Not parents (service providers) | Low SEP | Indicated | Child maltreatment | Service providers (delivering parenting program) | Home (via community or social service) | Service enhancement | Online modules | 1 | Not reported | SafeCare |
| Sheeber LB, Seeley JR, Feil EG, Davis B, Sorensen E, Kosty DB, et al. (2012) | Pilot evaluation (using randomised allocation and experimental design) | USA | 4.7 yrs (0.7) | 60% female | Low income | Selective | Maladaptive parenting | Parent | Home (via community or social service) | Self-directed learning + remote clinician contact | Online modules  Telephone calls  Social media | 3 | Other | Mom-Net |
| Sim WH, Fernando LMN, Jorm AF, Rapee RM, Lawrence KA, Mackinnon AJ, et al. (2020) | RCT | Australia | 9.79yrs | Not reported | Not reported | Selective | Maladaptive parenting | Parent | Home | Self-directed learning | Online modules  Telephone calls  Email | 3 | User-centred design | Parenting Resilient Kids (PaRK) |
| Sourander A, McGrath PJ, Ristkari T, Cunningham C, Huttunen J, Lingley-Pottie P, et al. (2016) | RCT | Finland | 4yo | 80% female | Not reported | Indicated | Maladaptive parenting | Parent | Home (via health service or hospital) | Self-directed learning + remote clinician contact | Online modules  Telephone calls | 2 | Not reported | Strongest Families Smart Website |
| Spence SH, Prosser SJ, March S, Donovan CL. (2020) | Quantitative non-randomised | Australia | 12.00 yrs (2.50) | Mother (90%), father (10%) | High SEP | Indicated | Maladaptive parenting | Parent | Home | Self-directed learning + remote clinician contact | Online modules  Videoconferencing  Telephone calls  Email | 4 | Not reported | BRAVE-ONLINE |
| Stalker KC, Rose RA, Bacallao M, Smokowski PR. (2018) | Evaluation study | USA | Not reported (range: 11-15) | 85% female | Low SEP | Selective | Maladaptive parenting | Parent + child | Home | Self-directed learning | Online modules | 1 | Other | Parenting Wisely |
| Stevens J, Scribano PV, Marshall J, Nadkarni R, Hayes J, Kelleher KJ. (2015) | RCT | USA | 5.1yrs | Women | Low income | Indicated | Interparental conflict | Parent | Home (via health service or hospital) | Remote clinician contact | Telephone calls | 1 | Community-based involvement | Not reported |
| Sung JY, Mumper E, Schleider JL. (2021) | RCT | USA | 6.77 yrs (1.93) | 92.3% female | Not reported | Indicated | Maladaptive parenting | Parent | Home | Self-directed learning | Online modules | 1 | Not reported | Project EMPOWER |
| Taylor LC, Leary KA, Boyle AE, Bigelow KE, Henry T, DeRosier M. (2015) | RCT | USA | 14.01yo | 89% female | Not reported | Selective | Maladaptive parenting | Parent | Home | Self-directed learning | Online modules | 1 | Not reported | Adolescent ParentWays |
| Thomson M. (2011) | Overview paper | Australia | N/A | 100% female | N/A | Indicated | Interparental conflict | N/A | Home (via community or social service) | Service enhancement | Videoconferencing  Telephone calls | 2 | Not reported | Family Dispute Resolution project |
| Tiwari A, Yuk H, Pang P, Fong DYT, Yuen F, Humphreys J, et al. (2012) | RCT | Hong Kong | Not reported | 74.9%female | Financial hardship | Indicated | Interparental conflict | Parent | Home | Remote clinician contact | Telephone calls | 1 | Not reported | Not reported |
| Traube DE, Hsiao HY, Rau A, Hunt-O’Brien D, Lu L, Islam N. (2020) | Pilot feasibility study | USA | M=9.86mo (0-3yo) | 100% female | Not reported | Selective | Child maltreatment | Parent + child | Home (via health service or hospital) | Remote clinician contact + peer support | Videoconferencing | 1 | Not reported | Parents as Teachers |
| Tully LA, Piotrowska PJ, Collins DAJ, Frick PJ, Anderson V, Moul C, et al. (2019) | Cross-sectional analytic study | Australia | M not reported (majority 6-9) | 85% female | Not reported | Universal | Maladaptive parenting | Parent | Home | Self-directed learning | Videos  Podcasts | 2 | Other | ParentWorks |
| Turner JJ, Kopystynska O, Bradford K, Schramm DG, Higginbotham BJ. (2021) | Analytical cross-sectional | USA | Not reported | 56% female | Not reported (average) | Indicated | Interparental conflict | Parents | Home (via community or social service) | Self-directed learning | Videos | 1 | Not reported | Online Divorce Education |
| van der Zanden RAP, Speetjens PAM, Arntz KSE, Onrust SA. (2010) | Pilot study | Netherlands | 6.7 years | 100% female | Not reported | Selective | Maladaptive parenting | Parent | Home | Self-directed learning + remote clinician contact | Social media  Email | 2 | Not reported | ChinUp Parents (KopUpOuders) |
| Wade SL, Cassedy AE, Shultz EL, Zang H, Zhang N, Kirkwood MW, et al. (2017) | RCT | USA | 5.4(2.2) | Not reported | Not reported | Indicated | Maladaptive parenting | Parent and child | Home | Self-directed learning + remote clinician contact | Videoconferencing  Online modules | 2 | User-centred design | i-INTERACT |
| White L, Delaney R, Pacifici C, Nelson C, Dickinson SL, Golzarri-Arroyo L. (2019) | RCT | USA | M not reported (range: 3-12 years) | 100% female | Not reported | Selective | Maladaptive parenting | Parent | Home (via community or social service) | Self-directed learning | Videos  Online modules | 2 | Not reported | Foster Parent College |
| Yap MBH, Mahtani S, Rapee RM, Nicolas C, Lawrence KA, Mackinnon A, et al. (2018) | RCT | Australia | 13.68 years (SD 1.06) | 5/7 female | Not reported (high education) | Selective | Maladaptive parenting | Parent + child | Home | Self-directed learning | Online modules  Telephone calls  Email | 3 | User-centred design | Partners in Parenting (PiP) |
| Yap MBH, Martin PD, Jorm AF. (2018) | Cross-sectional study | Australia | 14 yrs | 85.81% female | Not reported | Universal | Maladaptive parenting | Parent + child | Home | Self-directed learning | Website | 1 | Not reported | Parenting Strategies |
| Zlotnick C, Tzilos Wernette G, Raker CA. (2019) | RCT | USA | N/A (perinatal) | 100% male | Most receive public aid | Indicated | Interparental conflict | Parent | Home (via health service or hospital) | Self-directed learning + remote clinician contact | Online modules  Telephone calls | 2 | Not reported | Strength For U in Relationship Empowerment (SURE ) |

* = study included in secondary outcome analysis

## **Table 2.** Summary of Engagement Strategies and Engagement Measures of Studies Included in Primary Outcome, by Intervention Phases

| **Intervention**  **Phase** | **Study (main reference)** | **Engagement strategy(ies) reported and identified** | **Engagement strateg(ies) coded** | **Total delivery** | **Total design** | **Engagement measure(s) reported** | **Engagement measure(s) coded** | **Component of engagement** |
| --- | --- | --- | --- | --- | --- | --- | --- | --- |
| Design |  |  |  |  |  |  |  |  |
|  | Breitenstein SM, Shane J, Julion W, Gross D. Developing the eCPP: (2015) | Expert advisory group | *Design - expert consultation* | 0 | 6 | Talk aloud technique | *Think-aloud* | Qualitative |
|  |  | User advisory group | *Design - end user consultation* |  |  | Usability survey | *Feedback measures* | Qualitative |
|  |  | Pre-testing using 'talk aloud' technique | *Design - end user testing* |  |  |  |  |  |
|  |  | Provision of dinner, free childcare, and a $20 gift card | *Practical support* |  |  |  |  |  |
|  |  | User testing of components | *Design - end user testing* |  |  |  |  |  |
|  |  | Prototype program testing with users | *Design - end user testing* |  |  |  |  |  |
|  | Chu JTW, Wadham A, Jiang Y, Whittaker R, Stasiak K, Shepherd M, et al. (2019) | Targeted recruitment strategy | *Targeted recruitment strategy* | 2 | 1 | Qualitative analysis of discussions | *Focus groups* | Qualitative |
|  |  | Reimbursement (research assessment only) | *Research involvement - rewards* |  |  |  |  |  |
|  |  | Focus groups | *Design - end user consultation* |  |  |  |  |  |
|  | Fletcher R, Knight T, Macdonald JA, StGeorge J. (2019) | Expert advisory group | *Design - expert consultation* | 1 | 2 | None |  |  |
|  |  | Modified Delphi method | *Design - expert consultation* |  |  |  |  |  |
|  |  | Literacy screening | *Delivery - ease of use* |  |  |  |  |  |
|  | Fletcher R, May C, Wroe J, Hall P, Cooke D, Rawlinson C, et al. (2016) | Practitioner consultation | *Design - stakeholder consultation* | 1 | 4 | Acceptability interview | *Feedback measures* | Qualitative |
|  |  | Expert review | *Design - expert consultation* |  |  | Brief survey | *Feedback measures* | Qualitative |
|  |  | User consultation | *Design - end user consultation* |  |  |  |  |  |
|  |  | User testing | *Design - end user testing* |  |  |  |  |  |
|  |  | Text messages | *Delivery - mode* |  |  |  |  |  |
|  | Fox J, Archard PJ. (2018) | Stakeholder consultation- focus groups | *Design - stakeholder consultation* | 2 | 2 | Focus group | *Focus groups* | Qualitative |
|  |  | User consultation | *Design - end user consultation* |  |  |  |  |  |
|  |  | DVD educational videos | *Delivery - guidance (videos)* |  |  |  |  |  |
|  |  | Online directory of available resource | *Content - supplemental resources* | |  |  |  |  |
|  | Lawrence KA, Cardamone-Breen MC, Green J, Yap MBH, Jorm AF, Rapee RM. (2017) | Series of focus group workshops | *Design - end user consultation* | 0 | 1 | None |  |  |
|  | Love SM, Sanders MR, Metzler CW, Prinz RJ, Kast EZ. (2013) | Targeted recruitment strategy | *Targeted recruitment strategy* | 2 | 2 | Researcher observations | *Other: observation* | Qualitative |
|  |  | Focus groups | *Design - end user consultation* |  |  | Online survey | *Other: survey* | Qualitative |
|  |  | Online survey | *Design - end user consultation* |  |  |  |  |  |
|  |  | Gifts for participation | *Research involvement - rewards* |  |  |  |  |  |
|  | May CD, Fletcher R. (2019) | Reference group | *Design - stakeholder consultation* | 0 | 1 | Reviewer ratings | *Feedback measures* | Qualitative |
|  | Metzler CW, Sanders MR, Rusby JC, Crowley RN. (2012) | Focus group | *Design - end user consultation* | 1 | 2 | Response rates | *Enrolment rates* | Initial |
|  |  | Provision of prototype intervention for parent review | *Design - end user testing* |  |  |  |  |  |
|  |  | Tunnelled access through review activities | *Delivery - control features (tunnelling)* | | |  |  |  |
|  | Ragavan MI, Ferre V, Bair-Merritt M. (2020) | Stakeholder consultation | *Design - stakeholder consultation* | 0 | 4 | Mobile App Rating Scale | *Intensity (specific component usage)* | Quality |
|  |  | User consultation | *Design - end user consultation* |  |  | Qualitative interviews | *Interviews* | Qualitative |
|  |  | Stakeholder testing | *Design - stakeholder testing* | |  |  |  |  |
|  |  | User testing | *Design - end user testing* |  |  |  |  |  |
| Design + Delivery | |  |  |  |  |  |  |  |
|  | Choi H, Kim S, Ko H, Kim Y, Park CG. (2016) | Parent interviews about useful program content and format. | *Design - end user consultation* | 6 | 1 | Session evaluation | *Feedback measures* | Qualitative |
|  |  | Didactic exercises | *Delivery - guidance* |  |  | Retention | *Retention/Attrition/Dropout (study)* | Ongoing |
|  |  | Video clips modelling | *Delivery - guidance (videos)* |  |  | Qualitative interviews | *Interviews* | Qualitative |
|  |  | Personal feedback | *Delivery - personalisation* |  |  |  |  |  |
|  |  | Assignment and practice sessions | *Delivery - interactivity (rehearsal)* | |  |  |  |  |
|  |  | Q&A board | *Delivery - professional support features (non-clinical)* | | | | |  |
|  |  | Email reminders | *Content - reminders* |  |  |  |  |  |
|  | DeGarmo DS, Jones JA. (2019) | Stakeholder consultation (Judges, court administrators) | *Design - stakeholder consultation* | 10 | 2 | Attrition | *Retention/Attrition/Dropout (study)* | Ongoing |
|  |  | User consultation (Fathers) | *Design - end user consultation* |  |  | Module completion | *Completion rates (session/module)* | Ongoing |
|  |  | Video sequences | *Delivery - guidance (videos)* |  |  |  |  |  |
|  |  | Web-based interactivity | *Delivery - interactivity* |  |  |  |  |  |
|  |  | Web-based social connectivity and networking | *Content - social support features (discussion forum)* | | | | |  |
|  |  | Email/text prompting | *Content - reminders* |  |  |  |  |  |
|  |  | Electronic journal | *Delivery - personalisation* |  |  |  |  |  |
|  |  | Goal-setting | *Content - behaviour change techniques (goal setting)* | | | | |  |
|  |  | Knowledge tests | *Delivery - interactivity (challenge)* | |  |  |  |  |
|  |  | Printable materials | *Delivery - control features (reviewability)* | | | |  |  |
|  |  | Theory of instruction base | *Delivery - ease of use* |  |  |  |  |  |
|  |  | Tunnelling of core modules | *Delivery - control features (tunnelling)* | | |  |  |  |
|  | Fulgoni CMF, Melvin GA, Jorm AF, Lawrence KA, Yap MBH. (2019) | Content analysis of participant feedback | *Design - end user feedback* | 8 | 4 | Acceptability qualitative interview | *Feedback measures* | Qualitative |
|  |  | Qualitative interviews with parents piloting the program | *Design - end user feedback* |  |  | Feedback and input on in-session activities | *Interviews* | Qualitative |
|  |  | Online survey for mental health professionals | *Design - stakeholder consultation* | |  |  |  |  |
|  |  | Orientation module | *Delivery - ease of use* |  |  |  |  |  |
|  |  | Goal setting | *Content - behaviour change techniques (goal setting)* | | | | |  |
|  |  | Motivational interviewing to address barriers and enablers to goal setting | *Delivery - interactivity (reflection)* | |  |  |  |  |
|  |  | Coaching session | *Delivery - professional support features (clinical)* | | | |  |  |
|  |  | Self-selection of modules | *Delivery - control features* |  |  |  |  |  |
|  |  | Email summaries of coaching sessions | *Content - summaries* |  |  |  |  |  |
|  |  | Tailored feedback report | *Content - behaviour change techniques (feedback + tailoring)* | | | | |  |
|  |  | Interactive activities | *Delivery - interactivity* |  |  |  |  |  |
|  | Gelatt VA, Adler-Baeder F, Seeley JR. (2010) | Formative research | *Design - end user consultation* | 6 | 1 | Number of visits to program website | *Frequency (logins)* | Ongoing |
|  |  | Targeted recruitment strategy | *Targeted recruitment strategy* |  |  | Time spent on website | *Time or duration (program)* | Ongoing |
|  |  | Video vignette | *Delivery - narrative* |  |  | Number of individual pages viewed | *Intensity (module interaction)* | Ongoing |
|  |  | Interactive check-in questions | *Delivery - interactivity (challenge)* | |  | User satisfaction | *Satisfaction measures* | Qualitative |
|  |  | Downloadable resources in customised learning library | *Delivery - control features (reviewability)* | | | |  |  |
|  |  | Action Plan | *Content - behaviour change techniques (action plans)* | | | | |  |
|  |  | Rewards/incentives | *Research involvement - rewards* |  |  |  |  |  |
|  | Hamil J, Gier E, Garfield CF, Tandon D. (2021) | Expert consultation | *Design - expert consultation* | 5 | 3 | Qualitative interview | *Interviews* | Qualitative |
|  |  | Focus groups with service providers | *Design - stakeholder consultation* | |  | Session completion | *Completion rates (session/module)* | Ongoing |
|  |  | Focus groups with users | *Design - end user consultation* |  |  | Acceptability survey | *Feedback measures* | Qualitative |
|  |  | Practical support and reimbursement to attend focus groups and use text messaging functions. | *Practical support* |  |  | Post-intervention interviews | *Feedback measures* | Qualitative |
|  |  | Flexible delivery options- f2f or text messaging. | *Delivery - mode* |  |  |  |  |  |
|  |  | Worksheets | *Delivery - interactivity (challenge)* | |  |  |  |  |
|  |  | Text message reminders | *Content - reminders* |  |  |  |  |  |
|  |  | Videos | *Delivery - guidance (videos)* |  |  |  |  |  |
|  | Khor SPH, Fulgoni CM, Lewis D, Melvin GA, Jorm AF, Lawrence K, et al. (2021) | Parent stakeholder consultations | *Design - end user consultation* | 9 | 2 | Module completion | *Completion rates (session/module)* | Ongoing |
|  |  | Adolescent stakeholder consultations | *Design - end user consultation* |  |  | Satisfaction | *Satisfaction measures* | Qualitative |
|  |  | Tailored feedback report | *Content - behaviour change techniques (feedback + tailoring)* | | | Attrition | *Retention/Attrition/Dropout (study)* | Ongoing |
|  |  | Recommended AND Self-selection of modules | *Delivery - control features* |  |  |  |  |  |
|  |  | Tunnelled access to modules | *Delivery - control features (tunnelling)* | | |  |  |  |
|  |  | Email reminders | *Research involvement - reminders* |  |  |  |  |  |
|  |  | Activities and quizzes with feedback | *Delivery - interactivity (challenge)* | |  |  |  |  |
|  |  | Goal setting exercises | *Content - behaviour change techniques (goal setting)* | | | | |  |
|  |  | Therapist coaching via videoconferencing | *Delivery - professional support features (clinical)* | | | |  |  |
|  |  | Annotated slide emailed to parent | *Content - behaviour change techniques (feedback)* | | | |  |  |
|  |  | Reimbursement (research assessment only) | *Research involvement - rewards* |  |  |  |  |  |
|  | Metcalfe RE, Matulis JM, Cheng Y, Stormshak EA. (2021) | Focus groups (clinicians) | *Design - stakeholder consultation* | 10 | 3 | Think aloud technique | *Think-aloud* | Qualitative |
|  |  | Usability testing using think-aloud techniques | *Design - end user testing* |  |  | System Usability Scale | *Feedback measures* | Qualitative |
|  |  | Experience-based feedback | *Design - end user consultation* |  |  | Pilot feedback | *Feedback measures* | Qualitative |
|  |  | Tailored feedback | *Content - behaviour change techniques (feedback + tailoring)* | | | Time spent on active participation on website | *Intensity (specific component usage)* | Quality |
|  |  | Motivational interviewing techniques | *Delivery - interactivity (reflection)* | |  | Duration of time with therapeutic coach | *Time or duration (specific component)* | Quality |
|  |  | Practical strategies | *Delivery - guidance* |  |  | Therapeutic alliance survey | *Satisfaction measures* | Qualitative |
|  |  | Videos | *Delivery - guidance (videos)* |  |  | Consumer satisfaction survey | *Satisfaction measures* | Qualitative |
|  |  | Animations | *Delivery - guidance (animation)* |  |  |  |  |  |
|  |  | Choice to receive automated push text messages | *Delivery - control features + novelty* | |  |  |  |  |
|  |  | Online library | *Delivery - control features (reviewability)* | | | |  |  |
|  |  | Check-up summary | *Content - summaries* |  |  |  |  |  |
|  |  | Online coach via videoconferencing | *Delivery - professional support features (clinical)* | | | |  |  |
|  |  | Renumeration | *Research involvement - rewards* |  |  |  |  |  |
| Delivery |  |  |  |  |  |  |  |  |
|  | Agazzi H, Hayford H, Thomas N, Ortiz C, Salinas-Miranda A. A (2021)* | Lectures | *Delivery - guidance* | 7 | 0 | Participant satisfaction | *Satisfaction measures* | Qualitative |
|  |  | Videos | *Delivery - guidance (videos)* |  |  | Attendance | *Frequency (attendance)* | Ongoing |
|  |  | Group discussion | *Content - social support features (discussion forum)* | | | | |  |
|  |  | Class activities | *Delivery - interactivity* |  |  |  |  |  |
|  |  | Online delivery | *Delivery - mode* |  |  |  |  |  |
|  |  | Manuals mailed to homes | *Practical support* |  |  |  |  |  |
|  |  | Technology support calls | *Practical support* |  |  |  |  |  |
|  | Antonini TN, Raj SP, Oberjohn KS, Cassedy A, Makoroff KL, Fouladi M, et al. (2014)* | Demonstration videos | *Delivery - guidance (videos)* | 7 | 0 | Satisfaction with coaching | *Satisfaction measures* | Qualitative |
|  |  | Didactic exercises | *Delivery - guidance* |  |  | Adherence index | *Adherence* | Quality |
|  |  | Live coaching via videoconferencing | *Delivery - professional support features (clinical)* | | | Time spent online for study related activities | *Time or duration (specific component)* | Quality |
|  |  | Tailored didactive information | *Delivery - tailoring* |  |  |  |  |  |
|  |  | In-home first session | *Delivery - guidance* |  |  |  |  |  |
|  |  | Computer loaning | *Practical support* |  |  |  |  |  |
|  |  | $50 compensation | *Practical support* |  |  |  |  |  |
|  | Baggett K, Davis B, Feil E, Sheeber L, Landry S, Leve C, et al. (2017) | Targeted recruitment strategy | *Targeted recruitment strategy* | 8 | 0 | Satisfaction with remote coaching | *Satisfaction measures* | Qualitative |
|  |  | Video-based teaching | *Delivery - guidance (videos)* |  |  | Sessions completed | *Completion rates (session/module)* | Ongoing |
|  |  | Check-in questions | *Delivery - interactivity (challenge)* | |  | Satisfaction | *Satisfaction measures* | Qualitative |
|  |  | Individualised feedback | *Content - behaviour change techniques (feedback + personalisation)* | | | | | |
|  |  | Action planning | *Content - behaviour change techniques (action plans)* | | | | |  |
|  |  | Progression requirements | *Delivery - control features (tunnelling)* | | |  |  |  |
|  |  | Coach call with individualised support | *Delivery - professional support features (clinical)* | | | |  |  |
|  |  | Computer loaning and instruction | *Practical support* |  |  |  |  |  |
|  | Baggett KM, Davis B, Feil EG, Sheeber LL, Landry SH, Carta JJ, et al. (2010) | Videos with diverse samples | *Delivery - guidance (videos) + credibility* | 8 | 0 | Time spent - self-directed instructional pages | *Time or duration (specific component)* | Quality |
|  |  | Summary of key concepts | *Content - summaries* |  |  | Time spent - session summary pages | *Time or duration (specific component)* | Quality |
|  |  | Homework + check in questions | *Delivery - interactivity (challenge)* | |  | Time spent - daily activities | *Time or duration (specific component)* | Quality |
|  |  | Recorded mother-infant interactions | *Content - behaviour change techniques (feedback + personalisation)* | | | Time spent - video pages | *Time or duration (specific component)* | Quality |
|  |  | Coach telephone call for co-review and individualised support | *Delivery - professional support features (clinical)* | | | Time spent - coach call pages + Sense of connection | *Time or duration (specific component)* | Quality |
|  |  | Online bulletin board for group support | *Content - social support features (discussion forum)* | | | Time spent - bulletin board | *Time or duration (specific component)* | Quality |
|  |  | Ability to review content | *Delivery - control features (reviewability)* | | | Program completion | *Completion rates (program)* | Ongoing |
|  |  | Provision of laptop and internet | *Practical support* |  |  | Time spent in session | *Time or duration (specific component)* | Quality |
|  | Baggett KM, Davis B, Sheeber LB, Ammerman RT, Mosley EA, Miller K, et al. (2020) | Community outreach and capacity building | *Targeted recruitment strategy* | 1 | 2 | No. of consents | *Enrolment rates* | Initial |
|  |  | Partnerships with agencies | *Design - partnerships* |  |  | No. of initial session completions | *Completion rates (session/module)* | Ongoing |
|  |  | Recruitment video describing program for self-referral | *Targeted recruitment strategy* |  |  |  |  |  |
|  | Baker M, Biringen Z, Meyer-Parsons B, Schneider A. (2020) | Program materials mailed | *Practical support* | 6 | 0 | Activities completed | *Completion rates (specific component)* | Quality |
|  |  | Instructional videos | *Delivery - guidance (videos)* |  |  | Satisfaction rating | *Satisfaction measures* | Qualitative |
|  |  | Professional facilitator | *Delivery - professional support features (clinical)* | | | Satisfaction rating | *Satisfaction measures* | Qualitative |
|  |  | Self-reflective activities | *Delivery - interactivity (reflection)* | |  | Attendance | *Frequency (attendance)* | Ongoing |
|  |  | Group discussion | *Content - social support features (discussion forum)* | | | | |  |
|  |  | Individual session | *Delivery - personalisation* |  |  |  |  |  |
|  | Baker S, Sanders MR, Turner KMT, Morawska A. A (2017) | Reminders (emails and phone calls) | *Content - reminders* | 9 | 0 | Retention (Research) | *Retention/Attrition/Dropout (study)* | Ongoing |
|  |  | Practical/technical support | *Delivery - Professional support features (non-clinical)* | | | Time spent in module | *Time or duration (session or module)* | Ongoing |
|  |  | Reimbursement (research evaluation and clinic visit) | *Research involvement - rewards* |  |  | Feedback questionnaire | *Feedback measures* | Qualitative |
|  |  | Interactive exercises | *Delivery - interactivity* |  |  | Client satisfaction | *Satisfaction measures* | Qualitative |
|  |  | Personalised content | *Delivery - personalisation* |  |  |  |  |  |
|  |  | Semi-structured pathway through modules | *Delivery - control features (tunnelling)* | | |  |  |  |
|  |  | Videos modelling | *Delivery - guidance (videos)* |  |  |  |  |  |
|  |  | Downloadable resources | *Delivery - control features (reviewability)* | | | |  |  |
|  |  | Goal setting informed by values, beliefs, and traditions | *Content - Behaviour change techniques (Goal setting)* | | | | |  |
|  | Becher EH, Cronin S, McCann E, Olson KA, Powell S, Marczak MS. (2015) | Use of evaluation data and feedback to continually improve | *Design - iterative approach* | 5 | 1 | None |  |  |
|  |  | Reimbursement - raffle (research assessment only) | *Research involvement - rewards* |  |  |  |  |  |
|  |  | Videos (as education or modelling) | *Delivery - guidance (videos)* |  |  |  |  |  |
|  |  | Content quizzes | *Delivery - interactivity (challenge)* | |  |  |  |  |
|  |  | Visual diagrams and multimedia | *Delivery - aesthetics* |  |  |  |  |  |
|  | Bloom TL, Glass NE, Case J, Wright C, Nolte K, Parsons L. (2014) | Expert consultation | *Design - expert consultation* | 5 | 2 | Time taken to complete session | *Time or duration (session or module)* | Ongoing |
|  |  | User testing and feedback | *Design - end user testing* |  |  |  |  |  |
|  |  | Checklists and sliding bars | *Delivery - interactivity* |  |  |  |  |  |
|  |  | Tailored summary of preferences | *Delivery - tailoring* |  |  |  |  |  |
|  |  | Personalised safety plan | *Delivery - personalisation* |  |  |  |  |  |
|  |  | Ability to save and return and print | *Delivery - control features (reviewability)* | | | |  |  |
|  |  | Gift card incentives | *Research involvement - rewards* |  |  |  |  |  |
|  | Bodenmann G, Hilpert P, Nussbeck FW, Bradbury TN. (2014) | Technical support | *Delivery - Professional support features (non-clinical)* | 4 | 0 | Self-reported processing time (login and logout time) | *Time or duration (session or module)* | Ongoing |
|  |  | Interactive exercises | *Delivery - interactivity* |  |  | Self-reported technique implementation in daily life (Likert scale) | *Intensity (specific component usage)* | Quality |
|  |  | Tests, self-evaluation | *Delivery - interactivity (challenge + reflection)* | | | Drop-out rates | *Retention/Attrition/Dropout (study)* | Ongoing |
|  |  | Video examples | *Delivery - guidance (videos)* |  |  |  |  |  |
|  | Boekhorst MG, Hulsbosch LP, Nyklicek I, Spek V, Kastelein A, Bogels S, et al. (2021) | Trainer in video format | *Delivery - guidance (videos)* | 5 | 0 | Adherence (No. of sessions completed) | *Adherence* | Quality |
|  |  | Tasks and exercises | *Delivery - interactivity (challenge)* | |  |  |  |  |
|  |  | Additional written information about exercises | *Delivery - guidance* |  |  |  |  |  |
|  |  | Homework exercises | *Delivery - interactivity (rehearsal)* | |  |  |  |  |
|  |  | Prompts for reflective practice | *Delivery - interactivity (reflection)* | |  |  |  |  |
|  | Breaux R, Shroff DM, Cash AR, Swanson CS, Carlton C, Bertollo JR, et al. (2021)* | Targeted recruitment strategy | *Targeted recruitment strategy* | 7 | 0 | Attendance | *Frequency (attendance)* | Ongoing |
|  |  | Materials mailed to participants | *Practical support* |  |  | Acceptability survey | *Feedback measures* | Qualitative |
|  |  | Online delivery | *Delivery - mode* |  |  | Homework completion | *Completion rates (specific component)* | Quality |
|  |  | Interactive activities | *Delivery - interactivity* |  |  |  |  |  |
|  |  | Clinician involvement via videoconferencing | *Delivery - professional support features (clinical)* | | | |  |  |
|  |  | Weekly plans | *Content - behaviour change techniques (action plans)* | | | | |  |
|  |  | Point system to earn rewards | *Content - rewards* |  |  |  |  |  |
|  | Breitenstein SM, Fehrenbacher C, Holod AF, Schoeny ME. (2021) | All parents received a tablet computer | *Practical support* | 7 | 0 | Module completion | *Completion rates (session/module)* | Ongoing |
|  |  | Didactive teaching via video narration | *Delivery - guidance (videos)* |  |  | Satisfaction | *Satisfaction measures* | Qualitative |
|  |  | Video vignettes | *Delivery - narrative* |  |  |  |  |  |
|  |  | Questions about vignettes | *Delivery - interactivity (challenge)* | |  |  |  |  |
|  |  | Interactive activities | *Delivery - interactivity* |  |  |  |  |  |
|  |  | Sequential/tunnelled completion | *Delivery - control features (tunnelling)* | | |  |  |  |
|  |  | Automatic text messages to remind or reinforce completion | *Content - reminders* |  |  |  |  |  |
|  | Breitenstein SM, Fogg L, Ocampo EV, Acosta DI, Gross D. (2016)* | Advisory groups | *Design - stakeholder consultation* | 13 | 3 | Time spent on pages | *Time or duration (program)* | Ongoing |
|  |  | Prototype development and testing with stakeholders | *Design - stakeholder testing* |  |  | Time spent in modules | *Time or duration (session or module)* | Ongoing |
|  |  | Prototype development and testing with users | *Design - end user testing* |  |  | Number of program visits | *Frequency (logins)* | Ongoing |
|  |  | Targeted recruitment strategy | *Targeted recruitment strategy* |  |  | Module completion | *Completion rates (session/module)* | Ongoing |
|  |  | Tunnelled delivery | *Delivery - control features (tunnelling)* | | | Module activity completion | *Completion rates (specific component)* | Quality |
|  |  | Reviewability | *Delivery - control features (reviewability)* | | | Adherence Index | *Adherence* | Quality |
|  |  | Video vignettes of parent-child interactions | *Delivery - guidance (videos)* |  |  | Satisfaction | *Satisfaction measures* | Qualitative |
|  |  | Knowledge questions | *Delivery - interactivity (challenge)* | |  |  |  |  |
|  |  | Text reminders with encouraging language | *Content - reminders + rewards (praise)* | | | |  |  |
|  |  | Game activities | *Delivery - interactivity (gamification)* | | |  |  |  |
|  |  | Practice assignments | *Delivery - interactivity (rehearsal)* | |  |  |  |  |
|  |  | Bag of tricks' (collection of strategies parent has saved) | *Delivery - control features (reviewability)* | | | |  |  |
|  |  | Culturally and contextually relevant design | *Delivery - aesthetics + credibility* | |  |  |  |  |
|  |  | Badges | *Content - rewards* |  |  |  |  |  |
|  |  | Description and tutorial | *Practical support* |  |  |  |  |  |
|  |  | Loaning of tablets | *Practical support* |  |  |  |  |  |
|  | Brophy-Herb HE, Moyses K, Shrier C, Rymanowicz K, Pilkenton A, Dalimonte-Merckling D, et al. (2021) | Targeted recruitment strategy | *Targeted recruitment strategy* | 7 | 0 | Session completion | *Completion rates (session/module)* | Ongoing |
|  |  | Video examples | *Delivery - guidance (videos)* |  |  | Attrittion | *Retention/Attrition/Dropout (study)* | Ongoing |
|  |  | Personal reflection | *Delivery - interactivity (reflection)* | |  | Observation | *Other: observation* | Qualitative |
|  |  | Hands-on experiences | *Delivery - interactivity (rehearsal)* | |  |  |  |  |
|  |  | Anecdotes and scenarios | *Delivery - narrative* |  |  |  |  |  |
|  |  | Discussion forum (moderated by professional) | *Content - social support features (discussion forum)* | | | | |  |
|  |  | Materials to support PCIs. | *Practical support* |  |  |  |  |  |
|  | Cardamone-Breen MC, Jorm AF, Lawrence KA, Rapee RM, Mackinnon AJ, Yap MBH. (2018) | User consultation with reference group of parents of adolescents | *Design - end user consultation* | 3 | 1 | Amount of feedback read | *Intensity (specific component usage)* | Quality |
|  |  | Tailored feedback | *Content - behaviour change techniques (feedback + tailoring)* | | | Satisfaction questionnaire | *Satisfaction measures* | Qualitative |
|  |  | Actionable strategies | *Delivery - guidance* |  |  |  |  |  |
|  |  | Reimbursement (per research evaluation) | *Research involvement - rewards* |  |  |  |  |  |
|  | Cefai J, Smith D, Pushak RE. (2010)* | Basic instruction provision | *Practical support* | 7 | 0 | Qualitative questions | *Interviews* | Qualitative |
|  |  | Video clips modelling | *Delivery - guidance (videos)* |  |  | Satisfaction | *Satisfaction measures* | |
|  |  | Online delivery of program | *Delivery - mode* |  |  | Attrition- treatment | *Retention/Attrition/Dropout (intervention)* | |
|  |  | Option re. amount of program to complete | *Delivery - control features* |  |  | Attrition- research | *Retention/Attrition/Dropout (study)* | |
|  |  | Takehome material (workbook) | *Delivery - control features (reviewability)* | | | |  |  |
|  |  | Reflective activities | *Delivery - interactivity (reflection)* | |  |  |  |  |
|  |  | Multiple-choice question quiz | *Delivery - interactivity (challenge)* | |  |  |  |  |
|  | Choi H, Kim S, Ko H, Kim Y, Park CG. (2016) | Parent interviews about useful program content and format. | *Design - end user consultation* | 6 | 1 | Session evaluation | *Feedback measures* | |
|  |  | Didactic exercises | *Delivery - guidance* |  |  | Retention | *Retention/Attrition/Dropout (study)* | |
|  |  | Video clips modelling | *Delivery - guidance (videos)* |  |  | Qualitative interviews | *Interviews* |  |
|  |  | Personal feedback | *Delivery - personalisation* |  |  |  |  |  |
|  |  | Assignment and practice sessions | *Delivery - interactivity (rehearsal)* | |  |  |  |  |
|  |  | Q&A board | *Delivery - professional support features (non-clinical)* | | | | |  |
|  |  | Email reminders | *Content - reminders* |  |  |  |  |  |
|  | Comer JS, Furr JM, Miguel EM, Cooper-Vince CE, Carpenter AL, Elkins RM, et al. (2017)* | Incentives for assessment | *Research involvement - rewards* | 4 | 0 | Sessions until mastery | *Adherence* | Quality |
|  |  | Equipment kit provision | *Practical support* |  |  | Client Satisfaction Questionnaire | *Satisfaction measures* | Qualitative |
|  |  | Online delivery | *Delivery - mode* |  |  | Barriers to treatment | *Other: perceived barriers to treatment* | Qualitative |
|  |  | Live coaching via videoconferencing | *Delivery - professional support features (clinical)* | | | |  |  |
|  | Cotter KL, Bacallao M, Smokowski PR, Robertson CIB. (2013) | Video vignettes | *Delivery - guidance (videos)* | 4 | 0 | None |  |  |
|  |  | Feedback on answers about parenting choices | *Content - behaviour change techniques (feedback)* | | | |  |  |
|  |  | Self-paced | *Delivery - control features* |  |  |  |  |  |
|  |  | Reimbursement (research assessment only) | *Research involvement - rewards* |  |  |  |  |  |
|  | Czymoniewicz-Klippel M, Chesnut R, DiNallo J, Perkins D. (2019) | Online option | *Delivery - mode* | 6 | 0 | Satisfaction | *Satisfaction measures* | Qualitative |
|  |  | Testing instructions prior to starting program | *Practical support* |  |  | Engagement | *Feedback measures* | Qualitative |
|  |  | Email reminder to start program | *Content - reminders* |  |  | Qualitative interview | *Interviews* | Qualitative |
|  |  | Reflection activities on skills practice | *Delivery - interactivity (reflection)* | |  |  |  |  |
|  |  | Interactive activities | *Delivery - interactivity* |  |  |  |  |  |
|  |  | Multiple choice/short-answer questions | *Delivery - interactivity (challenge)* | |  |  |  |  |
|  | Dadds MR, Thai C, Mendoza Diaz A, Broderick J, Moul C, Tully LA, et al. (2019)* | eHealth option | *Delivery - mode* | 7 | 0 | Number of treatment sessions | *Completion rates (session/module)* | Ongoing |
|  | Study A | Video modules | *Delivery - guidance (videos)* |  |  | Duration of treatment sessions | *Time or duration (specific component)* | Quality |
|  |  | Interactive pop-up exercises | *Delivery - interactivity* |  |  | Therapeutic relationship | *Feedback measures* | Qualitative |
|  |  | Requirement to watch video before session | *Delivery - control features (tunnelling)* | | | Satisfaction | *Satisfaction measures* | Qualitative |
|  |  | Videoconference sessions with therapist | *Delivery - professional support features (clinical)* | | | Attrition (Research) | *Retention/Attrition/Dropout (study)* | Ongoing |
|  |  | Therapist unlocks module | *Delivery - control features (tunnelling)* | | |  |  |  |
|  |  | Accommodation for rural families to travel | *Practical support* |  |  |  |  |  |
|  | Study B | eHealth option | *Delivery - mode* | 7 | 0 | Number of treatment sessions | *Completion rates (session/module)* | Ongoing |
|  |  | Video modules | *Delivery - guidance (videos)* |  |  | Duration of treatment sessions | *Feedback measures* | Qualitative |
|  |  | Interactive pop-up exercises | *Delivery - interactivity* |  |  | Therapeutic relationship | *Feedback measures* | Qualitative |
|  |  | Requirement to watch video before session | *Delivery - control features (tunnelling)* | | | Satisfaction | *Satisfaction measures* | Qualitative |
|  |  | Videoconference sessions with therapist | *Delivery - professional support features (clinical)* | | | Attrition (Research) | *Retention/Attrition/Dropout (study)* | Ongoing |
|  |  | Therapist unlocks module | *Delivery - control features (tunnelling)* | | | Satisfaction | *Satisfaction measures* | Qualitative |
|  |  | Accommodation for rural families to travel | *Practical support* |  |  |  |  |  |
|  | Day JJ, Sanders MR. (2018)* | Videos to teach skills and demonstrate strategies | *Delivery - guidance (videos)* | 9 | 0 | Call completion | *Completion rates (specific component)* | Quality |
|  |  | Interactive activities | *Delivery - interactivity* |  |  | Call duration | *Time or duration (specific component)* | Quality |
|  |  | Downloadable resources | *Delivery - control features (reviewability)* | | | Module completion | *Completion rates (session/module)* | Ongoing |
|  |  | Dynamically generated workbook for tracking progress | *Content - behaviour change techniques (self-monitoring)* | | | Time spent in module | *Time or duration (session or module)* | Ongoing |
|  |  | Reminder texts | *Content - reminders* |  |  |  |  |  |
|  |  | Module summaries to partner (via email) | *Delivery - control features (reviewability)* | | | |  |  |
|  |  | Goal setting | *Content - behaviour change techniques (goal setting)* | | | | |  |
|  |  | Action planning (prevention plan) | *Content - behaviour change techniques (action plans)* | | | | |  |
|  |  | Practitioner support sessions via telephone | *Delivery - professional support features (clinical)* | | | |  |  |
|  | Donovan CL, March S. (2014) | Interactive features | *Delivery - interactivity* | 6 | 0 | Session completion | *Completion rates (session/module)* | Ongoing |
|  |  | Quizzes | *Delivery - interactivity (challenge)* | |  | Satisfaction | *Satisfaction measures* | Qualitative |
|  |  | Psychoeducation | *Delivery - guidance* |  |  |  |  |  |
|  |  | Practical strategies | *Delivery - guidance* |  |  |  |  |  |
|  |  | Telephone consultation with online therapist | *Delivery - professional support features (clinical)* | | | |  |  |
|  |  | Weekly emails to encourage and reinforce | *Delivery - professional support features (clinical)* | | | |  |  |
|  | Doss BD, Roddy MK, Llabre MM, Georgia Salivar E, Jensen-Doss A. (2020) | Personalised feedback | *Content - behaviour change techniques (feedback + personalisation)* | 3 | 0 | Time spent on call | *Time or duration (specific component)* | Quality |
|  |  | Self-selecting topic feature | *Delivery - control features* |  |  | Completion rates | *Completion rates (program)* | Ongoing |
|  |  | Coach call via telephone or video conferencing | *Delivery - professional support features (non-clinical)* | | | | |  |
|  | Ehrensaft MK, Knous-Westfall HM, Alonso TL. (2016) | Introduction by mentor to orient user | *Delivery - guidance* | 11 | 0 | Attrition | *Retention/Attrition/Dropout (study)* | Ongoing |
|  |  | Videos modelling | *Delivery - guidance (videos)* |  |  | Module completion | *Completion rates (session/module)* | Ongoing |
|  |  | Interactive exercises | *Delivery - interactivity* |  |  |  |  |  |
|  |  | Personalised goal setting | *Content - behaviour change techniques (goal setting + personalisation)* | | | | | |
|  |  | Personalised feedback | *Content - behaviour change techniques (feedback + personalisation)* | | | | | |
|  |  | Downloadable worksheets | *Delivery - control features (reviewability)* | | | |  |  |
|  |  | Personalised parent workbook | *Delivery - personalisation* |  |  |  |  |  |
|  |  | Tunnelling of module order | *Delivery - control features (tunnelling)* | | |  |  |  |
|  |  | Telephone reminders and rapport maintenance (by RA) | *Content - reminders* |  |  |  |  |  |
|  |  | Mailing to participants (birthday and holiday cards, etc.) | *Content - Social support features* |  |  |  |  |  |
|  |  | Reimbursement (research assessment only) | *Research involvement - rewards* |  |  |  |  |  |
|  | Enebrink P, Högström J, Forster M, Ghaderi A. (2012). | Videos of parent-child interaction | *Delivery - guidance (videos)* | 7 | 0 | Duration of support | *Time or duration (specific component)* | Quality |
|  |  | Session homework | *Delivery - interactivity (challenge + rehearsal)* | | | Sessions completed | *Completion rates (session/module)* | Ongoing |
|  |  | Supplemental resources | *Delivery - control features* |  |  |  |  |  |
|  |  | Multiple-choice questions | *Delivery - interactivity (challenge)* | |  |  |  |  |
|  |  | Online diary | *Content - behaviour change techniques (self-monitoring)* | | | | |  |
|  |  | Practitioner provision of tailored feedback | *Delivery - professional support features (clinical)* | | | |  |  |
|  |  | Monitored group discussion boards | *Content - social support features (discussion forum)* | | | | |  |
|  | Epstein M, Oesterle S, Haggerty KP. (2019) | Survey payment | *Incentives (research only)* | 5 | 0 | Activity log | *Completion rates (specific component)* | Quality |
|  |  | Updated images and language | *Delivery - message tone* |  |  | Qualitative analysis of posts | *Interviews* | Qualitative |
|  |  | Educational videos | *Delivery - guidance (videos)* |  |  | Completion of each chapter and activity | *Completion rates (specific component)* | Quality |
|  |  | Worksheets | *Delivery - interactivity (challenge)* | |  | Satisfaction | *Satisfaction measures* | Qualitative |
|  |  | Facebook group to facilitate completion and allow parents to connect | *Content - social support features (discussion forum)* | | | | |  |
|  | Farris JR, Bert SSC, Nicholson JS, Glass K, Borkowski JG. (2013) | Recorded lectures | *Delivery - guidance (videos)* | 4 | 0 | Attrition | *Retention/Attrition/Dropout (study)* | Ongoing |
|  |  | Parallel prompts | *Delivery - interactivity (challenge)* | |  | Compliance | *Completion rates (session/module)* | Ongoing |
|  |  | Weekly access | *Delivery - control features (tunnelling)* | | |  |  |  |
|  |  | Email reminders | *Content - reminders* |  |  |  |  |  |
|  | Fletcher R, Hammond C, Faulkner D, Turner N, Shipley L, Read D, et al. (2017) | Community partnership | *Design - partnerships* | 5 | 2 | Feedback | *Feedback measures* | Qualitative |
|  |  | Discussions with fathers and community members | *Design - end user consultation* |  |  | Clicks on pushed messages | *Intensity (module interaction)* | Ongoing |
|  |  | Videos made with participants | *Delivery - credibility* |  |  | Responses to pushed messages | *Intensity (specific component usage)* | Quality |
|  |  | Push text messages | *Delivery - mode* |  |  | Frequency of calls made | *Intensity (specific component usage)* | Quality |
|  |  | Mood Tracker | *Content - behaviour change techniques (self-monitoring)* | | | Visits to website | *Frequency (logins)* | Ongoing |
|  |  | Follow up calls from mentor (if distressed) | *Content - social support features* |  |  | Evaluation of website | *Feedback measures* | Qualitative |
|  |  | Website with community links and social media | *Content - social support features* |  |  |  |  |  |
|  | Feil EG, Baggett K, Davis B, Landry S, Sheeber L, Leve C, et al. (2020) | Video-based teaching | *Delivery - guidance (videos)* | 7 | 0 | Time spent in each component | *Time or duration (specific component)* | Quality |
|  |  | Check-in questions | *Delivery - interactivity (challenge)* | |  | Number of sessions | *Completion rates (session/module)* | Ongoing |
|  |  | Individualised feedback | *Content - behaviour change techniques (feedback + personalisation)* | | | Satisfaction | *Satisfaction measures* | Qualitative |
|  |  | Action planning | *Content - behaviour change techniques (action plans)* | | | | |  |
|  |  | Option of two languages | *Delivery - ease of use* |  |  |  |  |  |
|  |  | Coach call with individualised support | *Delivery - professional support features (clinical)* | | | |  |  |
|  |  | Computer loaning and instruction | *Practical support* |  |  |  |  |  |
|  | Ferraro AJ, Oehme K, Bruker M, Arpan L, Opel A. (2020) | Videos | *Delivery - guidance (videos)* | 5 | 0 | Video utility survey | *Feedback measures* | Qualitative |
|  |  | Professional narrator | *Delivery - credibility* |  |  |  |  |  |
|  |  | Images of diverse group of families | *Delivery - credibility* |  |  |  |  |  |
|  |  | Narration from children and couples | *Delivery - credibility* |  |  |  |  |  |
|  |  | Reflective prompts | *Delivery - interactivity (reflection)* | |  |  |  |  |
|  | Fleming GE, Kohlhoff J, Morgan S, Turnell A, Maiuolo M, Kimonis ER. (2021) | Practical support | *Practical support* | 2 | 0 | Treatment attrition | *Retention/Attrition/Dropout (intervention)* | Ongoing |
|  |  | Online mode | *Delivery - mode* |  |  | Satisfaction | *Satisfaction measures* | Qualitative |
|  |  |  |  |  |  | Homework compliance | *Completion rates (specific component)* | Quality |
|  | Fletcher R, Campbell L, Sved Williams A, Rawlinson C, Dye J, Baldwin A, et al. (2019) | Stakeholder consultation | *Design - stakeholder consultation* | 4 | 2 | Recruitment rates | *Enrolment rates* | Initial |
|  |  | User consultation | *Design - end user consultation* |  |  | Clicks on embedded links | *Intensity (module interaction)* | Ongoing |
|  |  | Targeted recruitment strategy | *Targeted recruitment strategy* |  |  | Response rates | *Intensity (module interaction)* | Ongoing |
|  |  | Links to resources | *Content - supplemental resources* | |  | Accetability survey | *Feedback measures* | Qualitative |
|  |  | Message schedule was tailored to gestation and infant’s age | *Delivery - tailoring* |  |  | Request to leave/dropout | *Retention/Attrition/Dropout (intervention)* | Ongoing |
|  |  | Interactive mood tracker | *Content - behaviour change techniques (self-monitoring)* | | | Qualitative usefulness interview | *Interviews* | Qualitative |
|  | Flujas-Contreras JM, Garcia-Palacios A, Gomez I. (2021) | 24/7 access | *Delivery - control features (all-at-once)* | 10 | 0 | Module completion | *Completion rates (session/module)* | Ongoing |
|  |  | Sequential organisation | *Delivery - control features (tunnelling)* | | | Satisfaction | *Satisfaction measures* | Qualitative |
|  |  | Values-based goal-setting | *Content - behaviour change techniques (goal setting)* | | | | |  |
|  |  | Video instruction | *Delivery - guidance (videos)* |  |  |  |  |  |
|  |  | Exercises | *Delivery - interactivity* |  |  |  |  |  |
|  |  | Downloadable worksheets | *Delivery - control features (reviewability)* | | | |  |  |
|  |  | Reminder emails | *Content - reminders* |  |  |  |  |  |
|  |  | Encouragement emails | *Content - reminders + rewards (praise)* | | | |  |  |
|  |  | Progress report | *Content - behaviour change techniques (feedback)* | | | |  |  |
|  |  | Ability to review/redo modules | *Delivery - control features (reviewability)* | | | |  |  |
|  | Ford-Gilboe M, Varcoe C, Scott-Storey K, Perrin N, Wuest J, Wathen CN, et al. (2020) | Developed for inclusiveness and fit for diverse groups | *Delivery - credibility* | 7 | 0 | Retention | *Retention/Attrition/Dropout (study)* | Ongoing |
|  |  | Interactive activities | *Delivery - interactivity* |  |  |  |  |  |
|  |  | Reimbursement (research assessment only) | *Research involvement - rewards* |  |  |  |  |  |
|  |  | Personalised detailed action plan of strategies and resources | *Content - behaviour change techniques (action plans + personalisation)* | | | | | |
|  |  | Wording to acknowledge and respect differences and uphold women's agency | *Delivery - message tone* |  |  |  |  |  |
|  |  | Ability to modify and further personalise plan | *Delivery - control features* |  |  |  |  |  |
|  |  | Reminders (research assessment only) | *Research involvement - reminders* |  |  |  |  |  |
|  | Franke N, Keown LJ, Sanders MR. (2020) | Audio-visual representation of information | *Delivery - guidance* | 3 | 0 | Consumer satisfaction | *Satisfaction measures* | Qualitative |
|  |  | Interactive exercises | *Delivery - interactivity* |  |  | Retention | *Retention/Attrition/Dropout (study)* | Ongoing |
|  |  | Telephone consultations to tailor implementation | *Delivery - professional support features (clinical)* | | | Program completion | *Completion rates (program)* | Ongoing |
|  | Ghaderi A, Kadesjo C, Bjornsdotter A, Enebrink P. (2018) | Provision of interpreters during recruitment | *Targeted recruitment strategy* | 5 | 0 | Initial engagement | *Completion rates (session/module)* | Ongoing |
|  |  | Videos of parent-child interactions | *Delivery - guidance (videos)* |  |  | Drop out | *Retention/Attrition/Dropout (intervention)* | Ongoing |
|  |  | Multiple-choice questions about content | *Delivery - interactivity (challenge)* | |  | Session completion | *Completion rates (session/module)* | Ongoing |
|  |  | Immediate feedback | *Content - behaviour change techniques (feedback)* | | | |  |  |
|  |  | Assessment incentives | *Research involvement - rewards* |  |  |  |  |  |
|  | Gulirmak K, Orak OS. (2020) | Videos | *Delivery - guidance* | 2 | 0 | None |  |  |
|  |  | Images, voiceover and written content | *Delivery - ease of use* |  |  |  |  |  |
|  | H. Mohammadinasab, M. Mazaheri, M. Reazaeizade, M. Heydari. (2020) | Qualitative interviews with parents | *Design - end user consultation* | 2 | 2 | None |  |  |
|  |  | Qualitative interviews with psychologists | *Design - stakeholder consultation* | |  | Enrolment | *Enrolment rates* | Initial |
|  |  | Video tutorials | *Delivery - guidance (videos)* |  |  |  |  |  |
|  |  | Use of a low-resource platform (WhatsApp) | *Delivery - mode* |  |  |  |  |  |
|  | Hegarty K, Tarzia L, Valpied J, Murray E, Humphreys C, Taft A, et al. (2019) | Targeted recruitment strategy | *Targeted recruitment strategy* | 6 | 0 | Completion rates | *Completion rates (specific component)* | Quality |
|  |  | Choice of modules | *Delivery - control features* |  |  | Process evaluation interview | *Interviews* | Qualitative |
|  |  | Contemplation Ladder to assess readiness for action | *Delivery - interactivity (reflection)* | |  | Retention rates (research only) | *Retention/Attrition/Dropout (study)* | Ongoing |
|  |  | Tailored action plan if ready | *Content - behaviour change techniques (action plans) + tailoring* | | | | | |
|  |  | Motivational interviewing component to prompt reflection if not ready | *Delivery - interactivity (reflection)* | |  |  |  |  |
|  |  | Problem-solving exercises | *Delivery - interactivity (challenge)* | |  |  |  |  |
|  | Hemdi A, Daley D. (2017) | Targeted recruitment strategy | *Targeted recruitment strategy* | 3 | 0 | Time spent reading manual (non-tech assisted) | *Time or duration (specific component)* | Quality |
|  |  | Therapist support | *Delivery - professional support features (clinical)* | | | Usefulness rating | *Satisfaction measures* | Qualitative |
|  |  | Flexible communication options | *Delivery - control features* |  |  | Attendance | *Frequency (attendance)* | Ongoing |
|  | Hinton S, Sheffield J, Sanders MR, Sofronoff K. (2017) | Video-based modelling of parenting skills | *Delivery - guidance (videos)* | 9 | 1 | Number of contacts | *Intensity (specific component usage)* | Quality |
|  |  | Parent-driven branching to review/gain information | *Delivery - control features* |  |  |  |  |  |
|  |  | Personalised goal setting | *Content - behaviour change techniques (goal setting)* | | | | |  |
|  |  | Probes/exercises to support mastery | *Delivery - interactivity (challenge)* | |  |  |  |  |
|  |  | Focus group consultation re. desired additional support | *Design - end user consultation* |  |  |  |  |  |
|  |  | Personalised timetable | *Content - behaviour change techniques (self-monitoring + personalisation)* | | | | | |
|  |  | Supplementary resources via mail | *Content - supplemental resources* | |  |  |  |  |
|  |  | Weekly telephone or email sessions | *Delivery - professional support features (clinical)* | | | |  |  |
|  |  | Reminders for questionnaires | *Research involvement - reminders* |  |  |  |  |  |
|  |  | Private Facebook 'parent support group' | *Content - social support features (discussion forum)* | | | | |  |
|  | Holden GW, Brown AS, Baldwin AS, Croft Caderao K. (2014) | Before and after quiz | *Delivery - interactivity (challenge)* | 1 | 0 | None |  |  |
|  | Hudson DB, Campbell-Grossman C, Hertzog M. (2012) | Electronic library | *Content - supplemental resources* | 5 | 0 | None |  |  |
|  |  | Discussion forum | *Content - social support features (discussion forum)* | | | | |  |
|  |  | Email dialogue | *Delivery - professional support features (clinical)* | | | |  |  |
|  |  | Loaning of all technological equipment | *Practical support* |  |  |  |  |  |
|  |  | Reimbursement (research assessment only) | *Research involvement - rewards* |  |  |  |  |  |
|  | Huebner DM, Rullo JE, Thoma BC, McGarrity LA, Mackenzie J. (2013) | Multi-level promotional campaign (national media, online searching, social networking, outreach to professional health orgs). | *Targeted recruitment strategy* | 5 | 1 | Responses to film- parent | *Feedback measures* | Qualitative |
|  |  | Focus groups with parents | *Design - end user consultation* |  |  | Responses to film- youth | *Feedback measures* | Qualitative |
|  |  | Emotionally-charged message tones | *Delivery - message tone* |  |  |  |  |  |
|  |  | Video guidance of behaviour | *Delivery - guidance (videos)* |  |  |  |  |  |
|  |  | Motivational interviewing approach | *Delivery - interactivity (reflection)* | |  |  |  |  |
|  |  | Ethically diverse characters | *Delivery - credibility* |  |  |  |  |  |
|  | Irvine AB, Gelatt VA, Hammond M, Seeley JR. (2015) | Video vignettes | *Delivery - narrative* | 8 | 0 | Satisfaction questionnaire | *Satisfaction measures* | Qualitative |
|  |  | Simplified reading level | *Delivery - ease of use* |  |  | Time spent per visit | *Time or duration (session or module)* | Ongoing |
|  |  | Check in questions | *Delivery - interactivity (challenge)* | |  |  |  |  |
|  |  | Multicultural cast of actors narrating as explanations | *Delivery - message tone + credibility* | |  |  |  |  |
|  |  | Reflective questions | *Delivery - interactivity (reflection)* | |  |  |  |  |
|  |  | Personal action plan | *Content - behaviour change techniques (action plans + personalisation)* | | | | | |
|  |  | Technical support | *Delivery - professional support features (non-clinical)* | | | | |  |
|  |  | Incentives | *Research involvement - rewards* |  |  |  |  |  |
|  | James Riegler L, Raj SP, Moscato EL, Narad ME, Kincaid A, Wade SL. (2020) | Focus groups | *Design - end user consultation* | 7 | 1 | Enrolment rates | *Enrolment rates* | Initial |
|  |  | Orientation session | *Practical support* |  |  | Sessions completed | *Completion rates (session/module)* | Ongoing |
|  |  | Didactic information | *Delivery - guidance* |  |  | Attrition | *Retention/Attrition/Dropout (intervention)* | |
|  |  | Video clips modelling skills | *Delivery - guidance (videos)* |  |  |  |  |  |
|  |  | Interactive exercises to reinforce | *Delivery - interactivity (rehearsal)* | |  |  |  |  |
|  |  | Tele-psychotherapy coaching | *Delivery - professional support features (clinical)* | | | |  |  |
|  |  | Skills practice between sessions | *Delivery - interactivity (rehearsal)* | |  |  |  |  |
|  |  | Information fed from parent to therapist | *Delivery - tailoring* |  |  |  |  |  |
|  | Jones DJ, Forehand R, Cuellar J, Parent J, Honeycutt A, Khavjou O, et al. (2014)* | Stakeholder consultation: researchers, clinicians, industry partners and health economists. | *Design - stakeholder consultation* | 9 | 2 | Consumer Satisfaction question | *Satisfaction measures* | Qualitative |
|  |  | Iterative feedback and modifications | *Design - iterative approach* |  |  | Attrittion | *Retention/Attrition/Dropout (study)* | Ongoing |
|  |  | Targeted recruitment strategy | *Targeted recruitment strategy* |  |  | Attendance | *Frequency (attendance)* | Ongoing |
|  |  | Provision of smartphone | *Practical support* |  |  | Work completion | *Completion rates (specific component)* | Quality |
|  |  | Short videos for psycho-ed and modelling | *Delivery - guidance (videos)* |  |  |  |  |  |
|  |  | Daily surveys | *Content - behaviour change techniques (self-monitoring)* | | | | |  |
|  |  | Mid-week video calls for reinforcement and feedback | *Delivery - professional support features (clinical)* | | | |  |  |
|  |  | Weekly videotaped practice | *Content - behaviour change techniques (feedback)* | | | |  |  |
|  |  | Text reminders | *Content - reminders* |  |  |  |  |  |
|  |  | Pushed content to parent | *Delivery - novelty* |  |  |  |  |  |
|  |  | Parent survey content fed to therapist | *Delivery - tailoring* |  |  |  |  |  |
|  | Jones DJ, Loiselle R, Zachary C, Georgeson AR, Highlander A, Turner P, et al. (2021)* | Targeted recruitment strategy | *Targeted recruitment strategy* | 12 | 0 | Session attendance | *Frequency (attendance)* | Ongoing |
|  |  | Online format | *Delivery - mode* |  |  | Mid-week call participation | *Completion rates (specific component)* | Quality |
|  |  | Short videos for psycho-ed and modelling | *Delivery - guidance (videos)* |  |  | Homework completion | *Completion rates (specific component)* | Quality |
|  |  | Daily surveys | *Content - behaviour change techniques (self-monitoring)* | | | Consumer satisfation | *Satisfaction measures* | Qualitative |
|  |  | Mid-week video calls for reinforcement | *Delivery - professional support features (clinical)* | | | |  |  |
|  |  | Weekly videotaped practice | *Content - behaviour change techniques (feedback)* | | | |  |  |
|  |  | Text reminders | *Content - reminders* |  |  |  |  |  |
|  |  | Pushed content to parent | *Delivery - novelty* |  |  |  |  |  |
|  |  | Parent survey content fed to therapist | *Delivery - tailoring* |  |  |  |  |  |
|  |  | Mobile application fed to therapist via web portal | *Delivery - tailoring* |  |  |  |  |  |
|  |  | Homework checklist | *Content - behaviour change techniques (self-monitoring)* | | | | |  |
|  |  | Reimbursement (research assessment only) | *Research involvement - rewards* |  |  |  |  |  |
|  | Jones S, Calam R, Sanders M, Diggle PJ, Dempsey R, Sadhnani V. (2014) | Audio-visual material to supplement workbook | *Content - supplemental resources* | 1 | 0 | Attrition (Research) | *Retention/Attrition/Dropout (study)* | Ongoing |
|  | Kaplan K, Solomon P, Salzer MS, Brusilovskiy E. (2014) | Expert consultation | *Design - expert consultation* | 4 | 1 | Feedback questionnaire | *Feedback measures* | Qualitative |
|  |  | Quiz | *Delivery - interactivity (challenge)* | |  | Quiz completion | *Completion rates (specific component)* | Quality |
|  |  | Homework assignments | *Delivery - interactivity (rehearsal)* | |  |  |  |  |
|  |  | Tailored to age of child | *Delivery - tailoring* |  |  |  |  |  |
|  |  | Online forum for peer support | *Content - social support features (discussion forum)* | | | | |  |
|  | Kavanagh DJ, Connolly J, Fisher J, Halford WK, Hamilton K, Hides L, et al. (2021)* | Encouraging and non-judgement tone | *Delivery - message tone* | 8 | 0 | Log-ins | *Frequency (logins)* | Ongoing |
|  |  | Goal-setting, problem-solving and behavioural activation tool | *Content - behaviour change techniques (goal-setting + action plans)* | | | Duration of use | *Time or duration (program)* | Ongoing |
|  |  | Scrapbook tool | *Delivery - personalisation* |  |  | Modules viewed | *Intensity (module interaction)* | Ongoing |
|  |  | Personalised dashboard | *Delivery - personalisation* |  |  | Program satisfaction | *Satisfaction measures* | Qualitative |
|  |  | SMS reminders | *Content - reminders* |  |  |  |  |  |
|  |  | Quizzes | *Delivery - interactivity (challenge)* | |  |  |  |  |
|  |  | Self- selection and no limitation on the pace of module access | *Delivery - control features* |  |  |  |  |  |
|  |  | Assessment rewards | *Research involvement - rewards* |  |  |  |  |  |
|  | Kirkman JJL, Hawes DJ, Dadds MR. (2016)* | eHealth option | *Delivery - mode* | 11 | 0 | Survey about engagement | *Feedback measures* | Qualitative |
|  |  | Website training session and take home use of manual | *Delivery - ease of use* |  |  | Module completion | *Completion rates (session/module)* | Ongoing |
|  |  | Video modules | *Delivery - guidance (videos)* |  |  | Satisfaction survey | *Satisfaction measures* | Qualitative |
|  |  | Interactive pop-up exercises | *Delivery - interactivity* |  |  | Number of logins | *Frequency (logins)* | Ongoing |
|  |  | Downloadable worksheets | *Delivery - control features (reviewability)* | | | Acceptability survey | *Feedback measures* | Qualitative |
|  |  | Automated text message and email reminders | *Content - reminders* |  |  | Survey about engagement- parent rated | *Feedback measures* | Qualitative |
|  |  | Parent subjective data fed to clinician | *Delivery - tailoring* |  |  | Satisfaction survey | *Satisfaction measures* | Qualitative |
|  |  | Moderated discussion board | *Content - social support features (discussion forum + professional support features)* | | | Survey about attitude to therapist | *Feedback measures* | Qualitative |
|  |  | Clinician unlocks modules | *Delivery - control features (tunnelling)* | | | The Scale to Assess the Therapeutic Relationship in Community Mental Health Care - patient version | *Feedback measures* | Qualitative |
|  |  | Unable to fast-forward | *Delivery - control features (tunnelling)* | | | The Scale to Assess the Therapeutic Relationship in Community Mental Health Care - clinician version | *Feedback measures* | Qualitative |
|  |  | Videoconferencing sessions with clinician | *Delivery - professional support features (clinical)* | | | Session attendance | *Frequency (attendance)* | Ongoing |
|  |  |  |  |  |  | Website acceptability | *Feedback measures* | Qualitative |
|  |  |  |  |  |  | Treatment retention | *Retention/Attrition/Dropout (intervention)* | Ongoing |
|  | Kopystynska O, Turner JJ, Schramm DG, Higginbotham B. (2020) | Videos | *Delivery - guidance (videos)* | 4 | 0 | None |  |  |
|  |  | Vignettes | *Delivery - narrative* |  |  |  |  |  |
|  |  | Checkpoint questions | *Delivery - interactivity (challenge)* | |  |  |  |  |
|  |  | Certificate of completion | *Content - rewards* |  |  |  |  |  |
|  | Koziol-McLain J, Vandal AC, Wilson D, Nada-Raja S, Dobbs T, McLean C, et al. (2018) | Targeted recruitment strategy | *Targeted recruitment strategy* | 7 | 0 | Retention | *Retention/Attrition/Dropout (study)* | Ongoing |
|  |  | Email reminders | *Research involvement - reminders* |  |  |  |  |  |
|  |  | Minor wording changes made for New Zealand context | *Delivery - message tone + credibility* | |  |  |  |  |
|  |  | Women choosing priority most important for them | *Content - behaviour change techniques (goal setting)* | | | | |  |
|  |  | Feedback (summarising priorities, scored feedback on level of danger) | *Content - behaviour change techniques (feedback + tailoring)* | | | | |  |
|  |  | Interactive process/activities | *Delivery - interactivity* |  |  |  |  |  |
|  |  | Tailored action plan | *Content - behaviour change techniques (action plans) + tailoring* | | | | | |
|  | Loew B, Rhoades G, Markman H, Stanley S, Pacifici C, White L, et al. (2012) | Interactive quizzes | *Delivery - interactivity (challenge)* | 10 | 0 | Module completion | *Completion rates (session/module)* | Ongoing |
|  |  | Dramatized vignettes | *Delivery - narrative* |  |  | Program feedback | *Feedback measures* | Qualitative |
|  |  | Feedback to quizzes | *Content - behaviour change techniques (feedback)* | | | Program satisfaction | *Satisfaction measures* | Qualitative |
|  |  | Video of narrator | *Delivery - guidance (videos)* |  |  |  |  |  |
|  |  | Printable hand-outs | *Delivery - control features (reviewability)* | | | |  |  |
|  |  | Reviewability of previous content | *Delivery - control features (reviewability)* | | | |  |  |
|  |  | Visual components of instructional content | *Delivery - aesthetics* |  |  |  |  |  |
|  |  | Navigational panel | *Delivery - guidance* |  |  |  |  |  |
|  |  | Text-only converting options | *Delivery - ease of use* |  |  |  |  |  |
|  |  | Comment sending features | *Content - social support features (discussion forum)* | | | | |  |
|  | Love SM, Sanders MR, Turner KMT, Maurange M, Knott T, Prinz R, et al. (2016) | Video-based modelling of parenting skills | *Delivery - guidance (videos)* | 13 | 0 | Satisfaction with the program | *Satisfaction measures* | Qualitative |
|  |  | Culturally diverse parent voxpops | *Delivery - credibility* |  |  | Focus groups | *Focus groups* | Qualitative |
|  |  | Personalised goal setting | *Content - behaviour change techniques (goal setting)* | | | Satisfaction with the program | *Satisfaction measures* | Qualitative |
|  |  | Review and feedback | *Content - behaviour change techniques (feedback)* | | | Module completion | *Completion rates (session/module)* | Ongoing |
|  |  | Interactive exercises | *Delivery - interactivity* |  |  |  |  |  |
|  |  | Downloadable worksheets and podcasts for session content review | *Delivery - control features (reviewability)* | | | |  |  |
|  |  | Automated text message and email prompts | *Content - reminders* |  |  |  |  |  |
|  |  | Responsive design for range of devices | *Delivery - mode* |  |  |  |  |  |
|  |  | Discussion boards | *Content - social support features (discussion forum)* | | | | |  |
|  |  | Badges' | *Content - rewards* |  |  |  |  |  |
|  |  | Virtual identity | *Content - social support features* |  |  |  |  |  |
|  |  | Accredited facilitator to moderate boards | *Delivery - professional support features (non-clinical)* | | | | |  |
|  |  | Provision of broadband to hosting agencies where needed | *Practical support* |  |  |  |  |  |
|  | Mast JE, Antonini TN, Raj SP, Oberjohn KS, Cassedy A, Makoroff KL, et al. (2014) | Demonstration videos | *Delivery - guidance (videos)* | 8 | 0 | Program feedback | *Feedback measures* | Qualitative |
|  |  | Didactic exercises | *Delivery - guidance* |  |  |  |  |  |
|  |  | Live coaching | *Delivery - professional support features (clinical)* | | | |  |  |
|  |  | Bi-weekly phone or email contact | *Delivery - professional support features (clinical)* | | | |  |  |
|  |  | Tailored didactive information | *Delivery - tailoring* |  |  |  |  |  |
|  |  | In-home first session | *Delivery - guidance* |  |  |  |  |  |
|  |  | Computer loaning | *Practical support* |  |  |  |  |  |
|  |  | $50 compensation | *Research involvement - rewards* |  |  |  |  |  |
|  | May CD, St George JM, Lane S. (2021) | Iterative process of development | *Design - iterative approach* | 3 | 3 | Clicks on pushed messages | *Intensity (module interaction)* | Ongoing |
|  |  | Working and reference group of multiple stakeholders | *Design - end user consultation* |  |  | Response rates | *Intensity (specific component usage)* | Quality |
|  |  | Reference group of multiple stakeholders | *Design - end user consultation* |  |  | Response ratings | *Satisfaction measures* | Qualitative |
|  |  | Pushed messages | *Delivery - novelty* |  |  | Response to final survey question | *Completion rates (program)* | Ongoing |
|  |  | Irregular scheduling | *Delivery - novelty* |  |  | Acceptability ratings | *Feedback measures* | Qualitative |
|  |  | Pushed messages for feedback | *Delivery - interactivity* |  |  |  |  |  |
|  | Mello MJ, Bromberg JR, Baird J, Wills H, Gaines BA, Lapidus G, et al. (2019) | Video scenarios | *Delivery - narrative* | 7 | 0 | Frequency of engagement | *Intensity (specific component usage)* | Quality |
|  |  | Skills practice | *Delivery - interactivity (rehearsal)* | |  | Frequency of engagement | *Intensity (specific component usage)* | Quality |
|  |  | Interactive quizzes | *Delivery - interactivity (challenge)* | |  | Acceptability survey | *Feedback measures* | Qualitative |
|  |  | Parent forum | *Delivery - professional support features (clinical)* | | | |  |  |
|  |  | Text messages | *Content - reminders* |  |  |  |  |  |
|  |  | Choice of theme to learn about | *Delivery - control features* |  |  |  |  |  |
|  |  | Gift card incentives | *Research involvement - rewards* |  |  |  |  |  |
|  | Morawska A, Tometzki H, Sanders MR. (2014) | Conversational format | *Delivery - message tone* | 4 | 0 | Attrition | *Retention/Attrition/Dropout (study)* | Ongoing |
|  |  | Downloadable podcasts | *Delivery - control features (reviewability)* | | | Satisfaction questionnaire | *Satisfaction measures* | Qualitative |
|  |  | Staggered delivery | *Delivery - control features (tunnelling)* | | |  |  |  |
|  |  | Email reminders | *Content - reminders* |  |  |  |  |  |
|  | Morgan AJ, Rapee RM, Bayer JK. (2016)* | Stakeholder consultation | *Design - stakeholder consultation* | 8 | 2 | No. of calls made | *Intensity (specific component usage)* | Quality |
|  |  | Usability testing | *Design - end user testing* |  |  | Program use: number of logins | *Frequency (logins)* | Ongoing |
|  |  | Email reminders after inactivity | *Content - reminders* |  |  | Program use: number of modules accessed | *Intensity (module interaction)* | Ongoing |
|  |  | Telephone support | *Delivery - professional support features (clinical)* | | | Program use: number of modules completed | *Completion rates (session/module)* | Ongoing |
|  |  | Virtual coach | *Delivery - guidance* |  |  | Program use: time spent logged in | *Time or duration (session or module)* | |
|  |  | Case examples | *Delivery - narrative* |  |  | Program use: reasons for not completing | *Other: reasons for non-completion* | Qualitative |
|  |  | Stories from other parents | *Delivery - narrative + credibility* |  |  | Satisfaction and feedback: frequency of practice | *Satisfaction measures* | Qualitative |
|  |  | Interactive worksheets | *Delivery - interactivity* |  |  |  |  |  |
|  |  | Online diary | *Content - behaviour change techniques (self-monitoring)* | | | | |  |
|  |  | All-at-once delivery | *Delivery - control features (all-at-once)* | | | |  |  |
|  | Morgan AJ, Rapee RM, Salim A, Goharpey N, Tamir E, McLellan LF, et al. (2017) | Stakeholder consultation | *Design - stakeholder consultation* | 12 | 2 | Questionnaire completion | *Retention/Attrition/Dropout (study)* | Ongoing |
|  |  | Usability testing | *Design - end user testing* |  |  | Program use: number of logins | *Frequency (logins)* | Ongoing |
|  |  | Responsive design on a range of devices | *Delivery - mode* |  |  | Program use: number of modules accessed | *Intensity (module interaction)* | Ongoing |
|  |  | Reminder and summary emails | *Content - reminders* |  |  | Program use: number of modules completed | *Completion rates (session/module)* | Ongoing |
|  |  | SMS reminders after inactivity | *Content - reminders* |  |  | Program use: time spent logged in | *Time or duration (program)* | Ongoing |
|  |  | Telephone support when requested | *Delivery - professional support features (clinical) + control features* | | | Program use: reasons for not completing | *Other: reasons for non-completion* | Qualitative |
|  |  | Virtual coach | *Delivery - guidance* |  |  | Satisfaction and feedback: frequency of practice | *Satisfaction measures* | Qualitative |
|  |  | Case examples | *Delivery - narrative* |  |  |  |  |  |
|  |  | Stories from other parents | *Delivery - narrative + credibility* |  |  |  |  |  |
|  |  | Interactive worksheets | *Delivery - interactivity* |  |  |  |  |  |
|  |  | Voucher incentives | *Research involvement - rewards* |  |  |  |  |  |
|  |  | Online diary | *Content - behaviour change techniques (self-monitoring)* | | | | |  |
|  |  | Tunnelled module delivery | *Delivery - control features (tunnelling)* | | |  |  |  |
|  |  | New content per module | *Delivery - novelty* |  |  |  |  |  |
|  | Murry VM, Berkel C, Liu N. (2018)* | User testing | *Design - end user testing* | 9 | 2 | Enrolment | *Enrolment rates* | Initial |
|  |  | Consideration of cultural relevance | *Design – stakeholder consultation* |  |  | Attendance | *Frequency (attendance)* | Ongoing |
|  |  | Culturally relevant avatars | *Delivery - credibility* |  |  | Satisfaction questionnaire | *Satisfaction measures* | Qualitative |
|  |  | Conjoint family sessions | *Content - social support features* |  |  | Attrittion | *Retention/Attrition/Dropout (study)* | Ongoing |
|  |  | Youth and parent specific card | *Delivery - interactivity (challenge)* | |  |  |  |  |
|  |  | Avatar modelling | *Delivery - guidance (animation)* |  |  |  |  |  |
|  |  | Visual guides | *Delivery - aesthetics* |  |  |  |  |  |
|  |  | Online option | *Delivery - mode* |  |  |  |  |  |
|  |  | Incentives | *Research involvement - rewards* |  |  |  |  |  |
|  |  | Technical assistance | *Practical support* |  |  |  |  |  |
|  |  | Provision of laptops | *Practical support* |  |  |  |  |  |
|  | Narad ME, Minich N, Taylor HG, Kirkwood MW, Brown TM, Stancin T, et al. (2015) | Initial face-to-face session with counsellor (goal setting and navigation) | *Delivery - professional support features (clinical)* | 5 | 0 | Sessions completed | *Completion rates (session/module)* | Ongoing |
|  |  | Downloadable resources | *Delivery - control features (reviewability)* | | | Time spent on website | *Time or duration (program)* | Ongoing |
|  |  | Video clips modelling skills | *Delivery - guidance (videos)* |  |  |  |  |  |
|  |  | Exercises and assignments | *Delivery - interactivity (challenge + rehearsal)* | | | |  |  |
|  |  | Videoconferencing sessions with counsellor | *Delivery - professional support features (clinical)* | | | |  |  |
|  | Nickerson AB, Livingston JA, Kamper-DeMarco K. (2018) | Videos (information and modelling) | *Delivery - guidance (videos)* | 5 | 0 | Attrition | *Retention/Attrition/Dropout (study)* | Ongoing |
|  |  | Pause and replay functions | *Delivery - control features (reviewability)* | | | |  |  |
|  |  | Email and text reminders | *Research involvement - reminders* |  |  |  |  |  |
|  |  | Tunnelled access and delivery of videos | *Delivery - control features (tunnelling)* | | |  |  |  |
|  |  | Reimbursement (research assessment only) | *Research involvement - rewards* |  |  |  |  |  |
|  | O’Shea A, Kaplan K, Solomon P, Salzer MS. (2019) | Quiz | *Delivery - interactivity (challenge)* | 5 | 0 | Frequency of reading, writing and receiving messages | *Intensity (specific component usage)* | Quality |
|  |  | Homework assignments | *Delivery - interactivity (rehearsal)* | |  |  |  |  |
|  |  | Tailored to age of child | *Delivery - tailoring* |  |  |  |  |  |
|  |  | Online forum for peer support | *Content - social support features (discussion forum)* | | | | |  |
|  |  | Pre-recorded video session as booster | *Delivery - guidance (videos)* |  |  |  |  |  |
|  | Olthuis JV, McGrath PJ, Cunningham CE, Boyle MH, Lingley-Pottie P, Reid GJ, et al. (2018) | Videos modelling skills | *Delivery - guidance (videos)* | 3 | 0 | Call completion | *Completion rates (specific component)* | Quality |
|  |  | Coach calls | *Delivery - professional support features (clinical)* | | | |  |  |
|  |  | Incentives | *Research involvement - rewards* |  |  |  |  |  |
|  | Owen DA, Hutchings J. (2017) | No downloading required | *Delivery - mode* | 10 | 0 | Feedback forms | *Feedback measures* | Qualitative |
|  |  | Video examples of positive parenting | *Delivery - guidance (videos)* |  |  | Session completion | *Completion rates (session/module)* | Ongoing |
|  |  | Setting achievable goals | *Content - behaviour change techniques (goal setting)* | | | | |  |
|  |  | Monitoring goals | *Content - behaviour change techniques (self-monitoring)* | | | | |  |
|  |  | Online feedback | *Content - behaviour change techniques (feedback)* | | | |  |  |
|  |  | Multiple-choice quizzes | *Delivery - interactivity (challenge)* | |  |  |  |  |
|  |  | All-at-once availability | *Delivery - control features (all-at-once)* | | | |  |  |
|  |  | Praise | *Content - rewards (praise)* |  |  |  |  |  |
|  |  | Audio options | *Delivery - ease of use* |  |  |  |  |  |
|  |  | Bullet-point format (minimal text), colourful images to complement text and audio buttons | *Delivery - ease of use* |  |  |  |  |  |
|  | Perrino T, Estrada Y, Huang S, St George S, Pantin H, Cano MA, et al. (2018) | Targeted recruitment strategy | *Targeted recruitment strategy* | 9 | 0 | Attendance | *Frequency (attendance)* | Ongoing |
|  |  | Facilitator led family sessions via videoconferencing | *Delivery - professional support features (clinical)* | | | Total participation | *Completion rates (program)* | Ongoing |
|  |  | Recording of group discussion amongst parents | *Delivery - guidance (videos)* |  |  | Initial engagement | *Completion rates (program)* | Ongoing |
|  |  | Culturally syntonic telenova | *Delivery - narrative* |  |  | Session completion | *Completion rates (session/module)* | Ongoing |
|  |  | Drag and drop activities | *Delivery - interactivity* |  |  |  |  |  |
|  |  | Identifying goals for adolescents | *Content - behaviour change techniques (goal setting)* | | | | |  |
|  |  | Programming prevented fast-forwarding | *Delivery - control features (tunnelling)* | | |  |  |  |
|  |  | Reflective questions on current parenting practices | *Delivery - interactivity (reflection)* | |  |  |  |  |
|  |  | Questionnaire reimbursement | *Research involvement - rewards* |  |  |  |  |  |
|  | Piotrowska PJ, Tully LA, Collins DAJ, Sawrikar V, Hawes D, Kimonis ER, et al. (2020) | Survey data to inform design | *Design - end user feedback* | 15 | 2 | Enrolment rates | *Enrolment rates* | Initial |
|  |  | Media campaign | *Targeted recruitment strategy* |  |  | Survey | *Satisfaction measures* | Qualitative |
|  |  | Inclusion of fathers as narrators and actors | *Delivery - credibility* |  |  | Satisfaction | *Satisfaction measures* | Qualitative |
|  |  | usability testing | *Design - end user testing* |  |  |  |  |  |
|  |  | Interactive video presentations | *Delivery - guidance (videos) + interactivity* | | | |  |  |
|  |  | Unlimited time for completions | *Delivery - control features* |  |  |  |  |  |
|  |  | Sequential unlocking to promote strategy implementation | *Delivery - control features (tunnelling)* | | |  |  |  |
|  |  | Recommended modules | *Delivery - personalisation* |  |  |  |  |  |
|  |  | Certificate of completion | *Content - rewards* |  |  |  |  |  |
|  |  | Motivational interviewing component for reflection and readiness | *Delivery - interactivity (reflection)* | |  |  |  |  |
|  |  | Father-specific design | *Delivery - aesthetics* |  |  |  |  |  |
|  |  | Exercises/worksheets | *Delivery - interactivity* |  |  |  |  |  |
|  |  | Downloadable homework sheets, workbook and tip sheets | *Delivery - control features (reviewability)* | | | |  |  |
|  |  | Goal setting | *Content - behaviour change techniques (goal setting)* | | | | |  |
|  |  | Review of progress | *Content - behaviour change techniques (self-monitoring)* | | | | |  |
|  |  | Problem solving | *Delivery - interactivity (challenge)* | |  |  |  |  |
|  |  | Tailored feedback assessment summaries | *Content - behaviour change techniques (feedback + tailoring)* | | | | |  |
|  |  | Email prompts | *Content - reminders* |  |  |  |  |  |
|  | Porzig-Drummond R, Stevenson RJ, Stevenson C. (2015) | Role play video vignettes | *Delivery - guidance (videos)* | 4 | 0 | Video completion | *Completion rates (session/module)* | Ongoing |
|  |  | Emailed tip sheet summaries | *Content - summaries* |  |  | Strategy implementation | *Intensity (specific component usage)* | Quality |
|  |  | Choice of delivery (DVD or online) | *Delivery - mode* |  |  | Satisfaction | *Satisfaction measures* | Qualitative |
|  |  | Email reminders | *Research involvement - reminders* |  |  |  |  |  |
|  | Potharst ES, Boekhorst MGBM, Cuijlits I, van Broekhoven KEM, Jacobs A, Spek V, et al. (2019) | Mindfulness trainer in video format | *Delivery - guidance (videos)* | 6 | 0 | Retention | *Retention/Attrition/Dropout (study)* | Ongoing |
|  |  | Formal meditation activities | *Delivery - interactivity (rehearsal)* | |  | Adherence | *Adherence* | Quality |
|  |  | Strategies for completing meditation | *Delivery - guidance* |  |  |  |  |  |
|  |  | Psychoeducation on mindful parenting | *Delivery - guidance* |  |  |  |  |  |
|  |  | Exercises for daily home practice | *Delivery - interactivity (rehearsal)* | |  |  |  |  |
|  |  | Invitation to write as reflection | *Delivery - interactivity (reflection)* | |  |  |  |  |
|  | Prinz RJ, Metzler CW, Sanders MR, Rusby JC, Cai C. (2021) | Module sequencing | *Delivery - control features (tunnelling)* | 9 | 0 | Program completion | *Completion rates (program)* | Ongoing |
|  |  | Video-based modelling | *Delivery - guidance (videos)* |  |  |  |  |  |
|  |  | Parental perspectives | *Delivery - credibility* |  |  |  |  |  |
|  |  | Goal-setting | *Content - behaviour change techniques (goal setting)* | | | | |  |
|  |  | Content review | *Delivery - control features (reviewability)* | | | |  |  |
|  |  | Feedback | *Content - behaviour change techniques (feedback)* | | | |  |  |
|  |  | Interactive exercises | *Delivery - interactivity* |  |  |  |  |  |
|  |  | Downloadable worksheets | *Delivery - control features (reviewability)* | | | |  |  |
|  |  | Podcasts | *Delivery - mode* |  |  |  |  |  |
|  |  | Customisable/printable workbook | *Delivery - control features (reviewability)* | | | |  |  |
|  |  | Technical and general telephone support calls | *Delivery - professional support features (non-clinical)* | | | | |  |
|  | Rabbitt SM, Carrubba E, Lecza B, McWhinney E, Pope J, Kazdin AE. (2016)* | Live or recorded formats | *Delivery - mode* | 4 | 0 | No. of telephone contacts made | *Intensity (specific component usage)* | Quality |
|  |  | Ability to re-watch old videos | *Delivery - control features (reviewability)* | | | Therapeutic alliance | *Satisfaction measures* | Qualitative |
|  |  | Role plays (active or passive) | *Delivery - guidance (videos)* |  |  | Adherence - parent | *Adherence* | Quality |
|  |  | Coach call with individualised support | *Delivery - professional support features (clinical)* | | | Adherence - therapist | *Adherence* | Quality |
|  | Raj SP, Shultz EL, Zang H, Zhang N, Kirkwood MW, Taylor HG, et al. (2018) | Live coaching via videoconferencing | *Delivery - professional support features (clinical)* | 4 | 0 | Attrition (research) | *Retention/Attrition/Dropout (study)* | Ongoing |
|  |  | Self-directed web content | *Delivery - control features* |  |  |  |  |  |
|  |  | Practical support | *Practical support* |  |  |  |  |  |
|  |  | Choice of supplemental sessions | *Delivery - control features* |  |  |  |  |  |
|  | Razuri EB, Hiles Howard AR, Parris SR, Call CD, DeLuna JH, Hall JS, et al. (2016) | Video instruction | *Delivery - guidance (videos)* | 4 | 0 | Attrition | *Retention/Attrition/Dropout (study)* | Ongoing |
|  |  | Video modelling | *Delivery - guidance (videos)* |  |  |  |  |  |
|  |  | Authentic examples in various contexts | *Delivery - credibility* |  |  |  |  |  |
|  |  | 24/7 availability | *Delivery - control features (all-at-once)* | | | |  |  |
|  | Richardson HL. (2021) | Targeted recruitment strategy | *Targeted recruitment strategy* | 5 | 0 | Enrolment rates | *Enrolment rates* | Initial |
|  |  | Video-based modelling | *Delivery - guidance (videos)* |  |  | Attrittion | *Retention/Attrition/Dropout (study)* | Ongoing |
|  |  | Choice of skills to view | *Delivery - control features* |  |  |  |  |  |
|  |  | Immediate feedback | *Content - behaviour change techniques (feedback)* | | | |  |  |
|  |  | Reimbursement (research assessment only) | *Research involvement - rewards* |  |  |  |  |  |
|  | Ristkari T, Kurki M, Suominen A, Gilbert S, Sinokki A, Kinnunen M, et al. (2019) | Targeted recruitment approach | *Targeted recruitment strategy* | 10 | 0 | Recruitment rate | *Enrolment rates* | Initial |
|  |  | Goal setting (during introductory call from coach) | *Content - behaviour change techniques (goal setting)* | | | Call duration | *Time or duration (specific component)* | Quality |
|  |  | Program progress summaries sent to staff | *Content - summaries* |  |  | Therapeutic alliance | *Satisfaction measures* | Qualitative |
|  |  | Non-binding program agreement form | *Content - behaviour change techniques (action plans)* | | | Time spent on website | *Time or duration (program)* | Ongoing |
|  |  | Video clips | *Delivery - guidance (videos)* |  |  | Attrition (Research) | *Retention/Attrition/Dropout (study)* | Ongoing |
|  |  | Interactive exercises | *Delivery - interactivity* |  |  |  |  |  |
|  |  | Action planning | *Content - behaviour change techniques (action plans)* | | | | |  |
|  |  | Supplemental resources | *Delivery - control features* |  |  |  |  |  |
|  |  | Reminders (automatic and coach) | *Content - reminders* |  |  |  |  |  |
|  |  | Coach calls (motivation and reflection) | *Delivery - professional support features (clinical)* | | | |  |  |
|  | Rizzo CJ, Houck C, Barker D, Collibee C, Hood E, Bala K. (2020) | Prototype testing with stakeholders | *Design – stakeholder testing* | 10 | 2 | Session debrief | *Feedback measures* | Qualitative |
|  |  | Acceptability testing with families | *Design - end user testing* |  |  | Module completion | *Completion rates (session/module)* | Ongoing |
|  |  | Games | *Delivery - interactivity (gamification)* | | | Retention | *Retention/Attrition/Dropout (study)* | Ongoing |
|  |  | Avatars narrating progress | *Delivery - guidance* |  |  |  |  |  |
|  |  | Activity prompts | *Delivery - interactivity* |  |  |  |  |  |
|  |  | Summaries | *Content - summaries* |  |  |  |  |  |
|  |  | Module summaries to partner (via email) | *Content - summaries* |  |  |  |  |  |
|  |  | Tunnelling (within module) | *Delivery - control features (tunnelling)* | | |  |  |  |
|  |  | Module pace (mostly) determined by parents and teens | *Delivery - control features* |  |  |  |  |  |
|  |  | Praise language | *Delivery - message tone* |  |  |  |  |  |
|  |  | Tech support from study staff | *Delivery - professional support features (non-clinical)* | | | | |  |
|  |  | Reimbursement (research assessment only) | *Research involvement - rewards* |  |  |  |  |  |
|  | Rudd BN, Holtzworth-Munroe A, Reyome JG, Applegate AG, D’Onofrio BM. (2015) | Certificate of completion | *Content - rewards* | 4 | 0 | None |  |  |
|  |  | Exercises (interactive activities) | *Delivery - interactivity* |  |  |  |  |  |
|  |  | Choice of commitments and exercises | *Delivery - control features* |  |  |  |  |  |
|  |  | Choice of when and where to complete program (before next court date) | *Delivery - control features* |  |  |  |  |  |
|  | Russell BS, Maksut JL, Lincoln CR, Leland AJ. (2016) | Live audio and chat capabilities | *Delivery - interactivity* | 3 | 0 | Client Satisfaction Questionnaire | *Satisfaction measures* | Qualitative |
|  |  | Group facilitator | *Delivery - professional support features (non-clinical)* | | | | |  |
|  |  | Hand-raising function | *Delivery - interactivity* |  |  |  |  |  |
|  | Sanders MR, Baker S, Turner KMT. (2012) | User-friendly navigation | *Delivery - ease of use* | 10 | 0 | Satisfaction- additional items | *Satisfaction measures* | Qualitative |
|  |  | Video demonstrations | *Delivery - guidance (videos)* |  |  | Satisfaction | *Satisfaction measures* | Qualitative |
|  |  | Parent-driven review features | *Delivery - control features* |  |  | Sessions completed | *Completion rates (session/module)* | Ongoing |
|  |  | Computer-assisted goal setting | *Content - behaviour change techniques (goal setting)* | | | | |  |
|  |  | Probes and exercises for mastery | *Delivery - interactivity (challenge + rehearsal)* | | | |  |  |
|  |  | Downloadable worksheets | *Delivery - control features (reviewability)* | | | |  |  |
|  |  | Podcasts to review | *Delivery - mode* |  |  |  |  |  |
|  |  | Printable notebook for review | *Delivery - control features (reviewability)* | | | |  |  |
|  |  | Reminder calls and emails | *Content - reminders* |  |  |  |  |  |
|  |  | Voucher incentives | *Research involvement - rewards* |  |  |  |  |  |
|  | Sanders MR, Dittman CK, Farruggia SP, Keown LJ. (2014)* | Video demonstrations | *Delivery - guidance (videos)* | 7 | 0 | Satisfaction | *Satisfaction measures* | Qualitative |
|  |  | Parent-driven branching to review or gain more information | *Delivery - control features* |  |  | Sessions completed | *Completion rates (session/module)* | Ongoing |
|  |  | Computer-assisted goal setting | *Content - behaviour change techniques (goal setting)* | | | | |  |
|  |  | Probes and exercises to check mastery | *Delivery - Interactivity* |  |  |  |  |  |
|  |  | Downloadable worksheets | *Delivery - control features (reviewability)* | | | |  |  |
|  |  | Podcasts | *Delivery - mode* |  |  |  |  |  |
|  |  | Printable notebook for goals and responses to exercises | *Delivery - control features (reviewability)* | | | |  |  |
|  | Sawyer A, Kaim A, Le HN, McDonald D, Mittinty M, Lynch J, et al. (2019) | Iterative process of development with mothers | *Design - end user testing* | 5 | 1 | Number of comments made and replied | *Intensity (specific component usage)* | Quality |
|  |  | Chat room | *Content - social support features (discussion forum)* | | | App logins | *Frequency (logins)* | Ongoing |
|  |  | Nurse facilitating and moderating intervention via an app | *Delivery - professional support features (clinical)* | | | Time spent in app | *Time or duration (program)* | Ongoing |
|  |  | Timeline of developmental milestones and mood ratings to mark complete | *Content - behaviour change techniques (self-monitoring)* | | | | |  |
|  |  | Informational resources | *Content - supplemental resources* | |  |  |  |  |
|  |  | Private messaging to nurse via app | *Delivery - professional support features (clinical)* | | | |  |  |
|  | Scholer SJ, Hudnut-Beumler J, Dietrich MS. (2010) | Targeted recruitment strategy | *Targeted recruitment strategy* | 4 | 0 | Recruitment rates | *Enrolment rates* | Initial |
|  |  | Video-based modelling | *Delivery - guidance (videos)* |  |  | Video completion | *Completion rates (session/module)* | Ongoing |
|  |  | Choice of skills to view | *Delivery - control features* |  |  |  |  |  |
|  |  | Language options | *Delivery - ease of use* |  |  |  |  |  |
|  | Schramm DG, McCaulley G. (2012)* | Online delivery of program | *Delivery - mode* | 4 | 0 | Satisfaction rating | *Satisfaction measures* | Qualitative |
|  |  | Quiz formats | *Delivery - interactivity (challenge)* | |  |  |  |  |
|  |  | Feedback to quizzes | *Content - behaviour change techniques (feedback)* | | | |  |  |
|  |  | Videos modelling positive and negative interactions | *Delivery - guidance (videos)* |  |  |  |  |  |
|  | Self-Brown S, Cowart-Osborne M, Baker E, Thomas A, Boyd C, Chege E, et al. (2015) | Motivational interviewing techniques | *Delivery - interactivity (reflection)* | 7 | 0 | Satisfaction survey | *Satisfaction measures* | Qualitative |
|  |  | Tailored feedback | *Content - behaviour change techniques (feedback + tailoring)* | | | Qualitative interview | *Interviews* | Qualitative |
|  |  | Culturally familiar avatar coach | *Delivery - credibility* |  |  |  |  |  |
|  |  | Video demonstrations | *Delivery - guidance (videos)* |  |  |  |  |  |
|  |  | Completion certificate | *Content - rewards* |  |  |  |  |  |
|  |  | Reimbursement (research assessment only) | *Research involvement - rewards* |  |  |  |  |  |
|  |  | Provision of tablet | *Practical support* |  |  |  |  |  |
|  | Self-Brown S, Reuben K, Perry EW, Bullinger LR, Osborne MC, Bielecki J, et al. (2020) | Virtual adaptation due to COVID-19 | *Delivery - mode* | 3 | 0 | Survey | *Feedback measures* | Qualitative |
|  |  | Text messaging | *Content - reminders* |  |  | None |  |  |
|  |  | Providing tablets or smart devices to families in need | *Practical support* |  |  |  |  |  |
|  | Self-Brown SR, C. Osborne M, Rostad W, Feil E. (2017) | Alpha and beta testing with parenting experts | *Design - expert testing* | 5 | 2 | Semi-structured interview | *Interviews* | Qualitative |
|  |  | Alpha and beta testing with parents | *Design - end user testing* |  |  |  |  |  |
|  |  | Videos | *Delivery - guidance (videos)* |  |  |  |  |  |
|  |  | Audio narration for all text | *Delivery - ease of use* |  |  |  |  |  |
|  |  | Engaging questions | *Delivery - interactivity* |  |  |  |  |  |
|  |  | Talk show manner | *Delivery - message tone* |  |  |  |  |  |
|  |  | Device compatibility | *Delivery - mode* |  |  |  |  |  |
|  | Sheeber LB, Seeley JR, Feil EG, Davis B, Sorensen E, Kosty DB, et al. (2012) | Focus on instructional design responsive to depressive symptoms | *Design- theory-based approach* | 9 | 1 | Time spent in calls | *Time or duration (specific component)* | Quality |
|  |  | Varied multimedia materials | *Delivery - ease of use* |  |  | Calls made | *Intensity (specific component usage)* | Quality |
|  |  | Interactive elements | *Delivery - interactivity* |  |  | Visits to bulletin | *Frequency (logins)* | Ongoing |
|  |  | Knowledge acquisition tests at each session | *Delivery - interactivity (challenge)* | |  | Modules completed | *Completion rates (session/module)* | Ongoing |
|  |  | Mastery before proceeding | *Delivery - control features (tunnelling)* | | | Program visits | *Frequency (logins)* | Ongoing |
|  |  | Feedback for incorrect answers | *Content - behaviour change techniques (feedback)* | | | Time spent on program | *Time or duration (program)* | Ongoing |
|  |  | Coach conference calls via telephone | *Delivery - professional support features (clinical)* | | | Satisfaction | *Satisfaction measures* | Qualitative |
|  |  | Motivational interviewing techniques | *Delivery - interactivity (reflection)* | |  | Homework experience | *Feedback measures* | Qualitative |
|  |  | Coach dashboard to monitor and tailor content | *Delivery - tailoring* |  |  | Therapeutic alliance | *Feedback measures* | Qualitative |
|  |  | Bulletin board | *Content - social support features (discussion forum)* | | | | |  |
|  | Sim WH, Fernando LMN, Jorm AF, Rapee RM, Lawrence KA, Mackinnon AJ, et al. (2020) | Tailored feedback | *Content - behaviour change techniques (feedback + tailoring)* | 7 | 0 | Intervention completion | *Completion rates (program)* | Ongoing |
|  |  | Recommended + self-selection of modules | *Delivery - control features* |  |  | Intervention adherence | *Adherence* | Quality |
|  |  | Tunnelled access to modules | *Delivery - control features (tunnelling)* | | |  |  |  |
|  |  | Email reminders | *Content - reminders* |  |  |  |  |  |
|  |  | Activities and quizzes with feedback | *Delivery - interactivity (challenge)* | |  |  |  |  |
|  |  | Goal setting exercises | *Content - behaviour change techniques (goal setting)* | | | | |  |
|  |  | Weekly check-in calls (not clinical) | *Delivery - professional support features (non-clinical)* | | | | |  |
|  | Sourander A, McGrath PJ, Ristkari T, Cunningham C, Huttunen J, Lingley-Pottie P, et al. (2016) | Exercises | *Delivery - interactivity (rehearsal)* | 8 | 0 | Satisfaction | *Satisfaction measures* | Qualitative |
|  |  | Instructional videos and Audio clips demonstrating skills | *Delivery - guidance (videos)* |  |  |  |  |  |
|  |  | Personalised with child's name, problems, strengths and preferred activities. | *Delivery - personalisation* |  |  |  |  |  |
|  |  | Coach call | *Delivery - professional support features (clinical)* | | | |  |  |
|  |  | Progression through mastery | *Delivery - control features (tunnelling)* | | |  |  |  |
|  |  | Booster session | *Delivery - interactivity (rehearsal)* | |  |  |  |  |
|  |  | Reminders of upcoming appointments if website visits are infrequent | *Content - reminders* |  |  |  |  |  |
|  |  | Targeted recruitment strategy | *Targeted recruitment strategy* |  |  |  |  |  |
|  | Spence SH, Prosser SJ, March S, Donovan CL. (2020) | Weekly email feedback and support | *Delivery - professional support features (clinical)* | 10 | 0 | Compliance | *Completion rates (session/module)* | Ongoing |
|  |  | Automated emails reminding of next session | *Content - reminders* |  |  |  |  |  |
|  |  | Tutorial on using the program | *Delivery - guidance* |  |  |  |  |  |
|  |  | Relaxation CD and handouts | *Delivery - control features (reviewability)* | | | |  |  |
|  |  | Visual appeal | *Delivery - aesthetics* |  |  |  |  |  |
|  |  | Minimal text | *Delivery - ease of use* |  |  |  |  |  |
|  |  | Quizzes | *Delivery - interactivity (challenge)* | |  |  |  |  |
|  |  | Automated feedback | *Content - behaviour change techniques (feedback)* | | | |  |  |
|  |  | Interactive games | *Delivery - interactivity (gamification)* | | |  |  |  |
|  |  | Alliance building activities | *Delivery - interactivity + credibility* | |  |  |  |  |
|  | Stalker KC, Rose RA, Bacallao M, Smokowski PR. (2018) | Targeted at-risk county (youth violence and socioeconomic disadvantage) for recruitment | *Targeted recruitment strategy* | 5 | 0 | Attrition (Research) | *Retention/Attrition/Dropout (study)* | Ongoing |
|  |  | Providing laptops with program materials to participants' homes | *Practical support* |  |  |  |  |  |
|  |  | Vignettes of parent-child interactions | *Delivery - guidance (videos)* |  |  |  |  |  |
|  |  | Interactive prompts and questions | *Delivery - interactivity (challenge)* | |  |  |  |  |
|  |  | Self-paced | *Delivery - control features* |  |  |  |  |  |
|  | Stevens J, Scribano PV, Marshall J, Nadkarni R, Hayes J, Kelleher KJ. (2015) | Advocacy support via telephone | *Delivery - professional support features (clinical)* | 3 | 0 | Number of calls made | *Intensity (specific component usage)* | Quality |
|  |  | Incentives | *Research involvement - rewards* |  |  | Time spent in call | *Time or duration (specific component)* | Quality |
|  |  | Practical support (free phone calls) | *Practical support* |  |  | Enrolment | *Enrolment rates* | Initial |
|  | Sung JY, Mumper E, Schleider JL. (2021) | Freedom to start at any time or place | *Delivery - control features* | 3 | 1 | Enrolment | *Enrolment rates* | Initial |
|  |  | Evidence-based design for single session interventions | *Design - evidence-based approach* | |  | Program Completion rates | *Completion rates (program)* | Ongoing |
|  |  | Personalised action plan | *Content - behaviour change techniques (action plans + personalisation)* | | | Accetability survey | *Feedback measures* | Qualitative |
|  |  | Interactive exercise | *Delivery - interactivity* |  |  | Attrition rates | *Retention/Attrition/Dropout (study)* | Ongoing |
|  | Taylor LC, Leary KA, Boyle AE, Bigelow KE, Henry T, DeRosier M. (2015) | Videos of in-person group discussions | *Delivery - guidance (videos)* | 6 | 0 | None |  |  |
|  |  | Supplemental homework activities | *Delivery - interactivity (challenge + rehearsal)* | | | |  |  |
|  |  | Instructional content | *Delivery - guidance* |  |  |  |  |  |
|  |  | Demonstration videos | *Delivery - guidance (videos)* |  |  |  |  |  |
|  |  | Tunnelled access to sessions (released weekly) | *Delivery - control features (tunnelling)* | | |  |  |  |
|  |  | Continued access to sessions | *Delivery - Control features* |  |  |  |  |  |
|  | Thomson M. (2011) | Subsidised fees | *Practical support* | 4 | 0 | None |  |  |
|  |  | Video streaming | *Delivery - mode* |  |  |  |  |  |
|  |  | Features to share visual information | *Delivery - guidance* |  |  |  |  |  |
|  |  | Note-taking features | *Delivery - interactivity* |  |  |  |  |  |
|  | Tiwari A, Yuk H, Pang P, Fong DYT, Yuen F, Humphreys J, et al. (2012) | Enhanced choice making and problem-solving skills | *Delivery - professional support features (clinical) + behaviour change techniques* | 2 | 0 | None |  |  |
|  |  | 24-hour access to a hotline | *Delivery - professional support features (clinical)* | | | |  |  |
|  | Traube DE, Hsiao HY, Rau A, Hunt-O’Brien D, Lu L, Islam N. (2020) | Parent-led sessions | *Delivery - control features* | 9 | 0 | Satisfaction questionnaire | *Satisfaction measures* | Qualitative |
|  |  | Assigned parent education | *Delivery - Professional support features (clinical)* | | | Satisfaction questionnaire | *Satisfaction measures* | Qualitative |
|  |  | Tailored planning | *Delivery - tailoring* |  |  |  |  |  |
|  |  | Group connections | *Content - social support features* |  |  |  |  |  |
|  |  | Written summaries of assessments | *Content - behaviour change techniques (feedback)* | | | |  |  |
|  |  | Goal setting (parent led) | *Content - behaviour change techniques (goal setting)* | | | | |  |
|  |  | Resource network/directory | *Content - supplemental resources* | |  |  |  |  |
|  |  | Loaning of tablets | *Practical support* |  |  |  |  |  |
|  |  | Activity kits | *Delivery - interactivity (rehearsal)* | |  |  |  |  |
|  | Tully LA, Piotrowska PJ, Collins DAJ, Frick PJ, Anderson V, Moul C, et al. (2019) | Focus groups to develop message content | *Design - end user consultation* | 3 | 1 | Exposure to campaign | *Other: exposure to material* | Ongoing |
|  |  | Delivery through a range of modes | *Delivery - mode* |  |  |  |  |  |
|  |  | Diverse cast in videos | *Delivery - credibility* |  |  |  |  |  |
|  |  | Well-known presenters in videos and radio | *Delivery - credibility* |  |  |  |  |  |
|  | Turner JJ, Kopystynska O, Bradford K, Schramm DG, Higginbotham BJ. (2021) | Checkpoint questions | *Delivery - interactivity (challenge)* | 8 | 0 | Survey completion (assessment only( | *Retention/Attrition/Dropout (study)* | Ongoing |
|  |  | Videos | *Delivery - guidance (videos)* |  |  |  |  |  |
|  |  | Narrated written slides | *Delivery - ease of use* |  |  |  |  |  |
|  |  | Vignettes | *Delivery - narrative* |  |  |  |  |  |
|  |  | Timed responses, requirement to restart if failure | *Delivery - control features (tunnelling)* | | |  |  |  |
|  |  | Prohibited from skipping sections | *Delivery - control features (tunnelling)* | | |  |  |  |
|  |  | Certificate of completion | *Content - rewards* |  |  |  |  |  |
|  |  | Ability to return later | *Delivery - control features (reviewability)* | | | |  |  |
|  | van der Zanden RAP, Speetjens PAM, Arntz KSE, Onrust SA. (2010) | Targeted recruitment strategy | *Targeted recruitment strategy* | 8 | 0 | Course satisfaction- subscale | *Satisfaction measures* | Qualitative |
|  |  | Chat sessions with peers | *Content - social support features (discussion forum)* | | | Course satisfaction- subscale | *Satisfaction measures* | Qualitative |
|  |  | Videos | *Delivery - guidance (videos)* |  |  | Course satisfaction- subscale | *Satisfaction measures* | Qualitative |
|  |  | Home exercises | *Delivery - interactivity (rehearsal)* | |  | Course satisfaction- subscale | *Satisfaction measures* | Qualitative |
|  |  | Professional facilitator | *Delivery - professional support features (clinical)* | | | Course adherence | *Adherence* | Quality |
|  |  | Action plans | *Content - behaviour change techniques (action plans)* | | | Dropouts | *Retention/Attrition/Dropout (study)* | Ongoing |
|  |  | Parenting atmosphere meter | *Content - behaviour change techniques (self-monitoring)* | | | | |  |
|  |  | Downloadable transcripts | *Delivery - control features (reviewability)* | | | |  |  |
|  | Wade SL, Cassedy AE, Shultz EL, Zang H, Zhang N, Kirkwood MW, et al. (2017) | Targeted recruitment strategy | *Targeted recruitment strategy* | 16 | 2 | Session completion | *Completion rates (session/module)* | Ongoing |
|  |  | Reimbursement | *Practical support* |  |  |  |  |  |
|  |  | Stakeholder consultation: consultants and program developers (Express) | *Design - stakeholder consultation* | |  |  |  |  |
|  |  | User consultation (Express) | *Design - end user consultation* |  |  |  |  |  |
|  |  | Assessor set up computer and internet connections | *Practical support* |  |  |  |  |  |
|  |  | Loaning of computer materials | *Practical support* |  |  |  |  |  |
|  |  | Program instruction | *Practical support* |  |  |  |  |  |
|  |  | Program engagement info sent to therapist | *Delivery - tailoring* |  |  |  |  |  |
|  |  | Fixed order, tunnelling | *Delivery - control features (tunnelling)* | | |  |  |  |
|  |  | Option for supplemental sessions | *Delivery - control features* |  |  |  |  |  |
|  |  | Goal setting with therapist | *Content - behaviour change techniques (goal setting)* | | | | |  |
|  |  | Videos narrating experiences | *Delivery - narrative* |  |  |  |  |  |
|  |  | Videos modelling skills | *Delivery - guidance (videos)* |  |  |  |  |  |
|  |  | Exercises to practice skills | *Delivery - interactivity (rehearsal)* | |  |  |  |  |
|  |  | Therapy sessions via videoconferencing | *Delivery - professional support features (clinical)* | | | |  |  |
|  |  | Range of formats (written, audio, video) | *Delivery - mode* |  |  |  |  |  |
|  |  | Therapist availability during non-traditional hours | *Delivery - professional support features (clinical)* | | | |  |  |
|  |  | Reminder texts, calls and emails | *Content - reminders* |  |  |  |  |  |
|  | White L, Delaney R, Pacifici C, Nelson C, Dickinson SL, Golzarri-Arroyo L. (2019) | Group discussion board exercises (monitored by staff) | *Content - social support features (discussion forum + professional support features)* | 6 | 0 | Attrition (Research) | *Retention/Attrition/Dropout (study)* | Ongoing |
|  |  | Interactive exercises | *Delivery - interactivity* |  |  | User satisfaction | *Satisfaction measures* | Qualitative |
|  |  | Tunnelled access/order of online meetings | *Delivery - control features (tunnelling)* | | | Feedback questionnaire | *Feedback measures* | Qualitative |
|  |  | Portrayal of children’s' stories | *Delivery - narrative* |  |  | Time spent in workshop | *Time or duration (session or module)* | Ongoing |
|  |  | Reimbursement (per research evaluation) | *Research involvement - rewards* |  |  |  |  |  |
|  |  | Certificate of completion (online training) | *Content - rewards* |  |  |  |  |  |
|  | Yap MBH, Mahtani S, Rapee RM, Nicolas C, Lawrence KA, Mackinnon A, et al. (2018) | Parent stakeholder consultations | *Design - end user consultation* | 7 | 2 | Intervention completion | *Completion rates (program)* | Ongoing |
|  |  | Adolescent stakeholder consultations | *Design - end user consultation* |  |  | Intervention adherence | *Adherence* | Quality |
|  |  | Tailored feedback report | *Content - behaviour change techniques (feedback + tailoring)* | | | Attrition | *Retention/Attrition/Dropout (study)* | Ongoing |
|  |  | Self-selection of modules | *Delivery - control features* |  |  |  |  |  |
|  |  | Tunnelled access to modules | *Delivery - control features (tunnelling)* | | |  |  |  |
|  |  | Email reminders | *Content - reminders* |  |  |  |  |  |
|  |  | Activities and quizzes with feedback | *Delivery - interactivity (challenge)* | |  |  |  |  |
|  |  | Goal setting exercises | *Content - behaviour change techniques (goal setting)* | | | | |  |
|  |  | Weekly check-in calls (not clinical) | *Delivery - professional support features (non-clinical)* | | | | |  |
|  | Yap MBH, Martin PD, Jorm AF. (2018) | Practical strategies | *Delivery - guidance* | 2 | 0 | Open-ended question on usefulness and feedback | *Feedback measures* | Qualitative |
|  |  | Downloadable resource | *Delivery - control features (reviewability)* | | | |  |  |
|  | Zlotnick C, Tzilos Wernette G, Raker CA. (2019) | Parrot avatar with female voice | *Delivery - guidance* | 11 | 0 | Calls completed | *Completion rates (specific component)* | Quality |
|  |  | Talks to user by their name | *Delivery - personalisation* |  |  | Satisfaction | *Satisfaction measures* | Qualitative |
|  |  | Content read aloud | *Delivery - ease of use* |  |  |  |  |  |
|  |  | Motivational interviewing | *Delivery - interactivity (reflection)* | |  |  |  |  |
|  |  | Collaborative and non-confrontational | *Delivery - message tone* |  |  |  |  |  |
|  |  | Range of resources presented | *Delivery - control features* |  |  |  |  |  |
|  |  | Personalised safety plan | *Content - behaviour change techniques (action plans)* | | | | |  |
|  |  | Videos (of other women's experiences) | *Delivery - narrative + credibility* |  |  |  |  |  |
|  |  | Printouts | *Delivery - control features (reviewability)* | | | |  |  |
|  |  | Assessment rewards | *Research involvement - rewards* |  |  |  |  |  |
|  |  | Booster session via telephone | *Delivery - professional support features (clinical)* | | | |  |  |

* = study included in secondary outcome analysis

## **Table 3.** Summary of Between-Group Engagement Results of Studies Included in Secondary Outcome

| **Study** | **Program name** | **Engagement measure(s) coded** | **Component of engagement** | **Engagement outcome statistic** | **p-value** | **Interpretation comments** |
| --- | --- | --- | --- | --- | --- | --- |
| Agazzi H, Hayford H, Thomas N, Ortiz C, Salinas-Miranda A. A (2021)* | iHOT-DOCTS (BPT) | *Satisfaction measures* | Qualitative | p=<.01 | p=<.01 | Participant satisfaction scores (TAI) were significantly different between caregivers in the two class types, with higher mean scores for the in-person modality compared to telehealth |
|  |  | *Frequency (attendance)* | Ongoing | p=.67 | p=.67 | Almost all of the attendees attended at least five sessions. |
| Antonini TN, Raj SP, Oberjohn KS, Cassedy A, Makoroff KL, Fouladi M, et al. (2014)* | i-INTERACT | *Satisfaction measures* | Qualitative |  |  |  |
|  |  | *Adherence* | Quality |  |  |  |
|  |  | *Time or duration (specific component)* | Quality | χ2(3, N = 32) =7.03, p = .07 | p = .07 | There were no significant group differences in reported time spent online for study-related didactic activities. |
| Breaux R, Shroff DM, Cash AR, Swanson CS, Carlton C, Bertollo JR, et al. (2021)* | RELAX | *Frequency (attendance)* | Ongoing |  |  |  |
|  |  | *Feedback measures* | Qualitative | ts = 0.00−2.06, ps > .05 | ps > .05 | No significant differences in feedback were found between the in-person and telehealth groups (except for adolescent report of utility of session handouts). |
|  |  | *Completion rates (specific component)* | Quality |  |  |  |
| Breitenstein SM, Fogg L, Ocampo EV, Acosta DI, Gross D. (2016)* | ezParent (adapted from Chicago Parent Program) | *Time or duration (program)* | Ongoing |  |  |  |
|  |  | *Time or duration (session or module)* | Ongoing |  |  |  |
|  |  | *Frequency (logins)* | Ongoing |  |  |  |
|  |  | *Completion rates (session/module)* | Ongoing | 85.4% (34/40) of modules (95% CI = 74.5%-96.4%),*p* <.05 | p <.05 | The ezPARENT dose rate was higher than the upper CI for the group CPP dose, suggesting that parent participation rates of the ezPARENT and group based CPP were significantly different. Across all six modules, the average ezPARENT module completion by parent was 85.4%. In contrast, average parent attendance for the face-to-face CPP groups was 50.6%. |
|  |  | *Completion rates (specific component)* | Quality |  |  |  |
|  |  | *Adherence* | Quality |  |  |  |
|  |  | *Satisfaction measures* | Qualitative | Kendall’s Tau-b = 0.25; P<0.05 | p<0.05 | Parents in the ezP ARENT group were more likely to recommend the ezP ARENT Program to other parents than parents recommending the health promotion site. |
| Cefai J, Smith D, Pushak RE. (2010)* | Parenting Wisely | *Interviews* | Qualitative |  |  |  |
|  |  | *Satisfaction measures* | Qualitative | x2(3, N = 83) = 9.95, p = .02, Cramer’s V = .35; 11.10, p = .01, Cramer’s V = .37 | | Parents in the individual format found the program to be significantly more enjoyable and were more satisfied than parents in the group format. |
|  |  | *Retention/Attrition/Dropout (intervention)* | | | |  |
|  |  | *Retention/Attrition/Dropout (study)* | | | |  |
| Comer JS, Furr JM, Miguel EM, Cooper-Vince CE, Carpenter AL, Elkins RM, et al. (2017)* | Internet Parent Child Interaction Therapy (I-PCIT) | *Adherence* | Quality | t(26) = 0.35, p = .73 | p = .73 | Number of sessions did not differ across conditions |
|  |  | *Satisfaction measures* | Qualitative |  |  |  |
|  |  | *Other: perceived barriers to treatment* | Qualitative | F(3,29) = 7.62, p = .001 | p = .001 | I-PCIT was associated with fewer barriers to treatment participation than clinic-based PCIT. |
| Czymoniewicz-Klippel M, Chesnut R, DiNallo J, Perkins D. (2019)* | Grow Online | *Satisfaction measures* | Qualitative | t(45)= 3.27, p = 0.002 | p = 0.002 | Grow F2F ratings were higher than Grow Online ratings. |
|  |  | *Feedback measures* | Qualitative | Not reported | 0.5 (Not reported) | Grow F2F ratings were higher than Grow Online ratings. |
|  |  | *Interviews* | Qualitative |  |  |  |
| Dadds MR, Thai C, Mendoza Diaz A, Broderick J, Moul C, Tully LA, et al. (2019)* | AccessEI | *Completion rates (session/module)* | Ongoing | *t(112)= 1.348, p = .001* | p = .001 | Families in the AccessEI group had a significantly greater number of treatment sessions than those in face-to-face. |
|  |  | *Time or duration (specific component)* | Quality | t(62)= 2.86, p= .006. | p= .006 | Families in the AccessEI also had a significant greater overall treatment time in minutes than families in face-to-face. |
|  |  | *Feedback measures* | Qualitative | Not reported | 0.5 (Not reported) | |
|  |  | *Feedback measures* | Qualitative | F(1,39) = 3.91, p = .05 | p = .05 | The relationship with the clinician was rated higher for face-to-face than AccessEI. |
|  |  | *Satisfaction measures* | Qualitative | Not reported | 0.5 (Not reported) | |
|  |  | *Satisfaction measures* | Qualitative | Not reported | 0.5 (Not reported) | |
|  |  | *Retention/Attrition/Dropout (study)* | Ongoing |  |  |  |
|  | AccessEI | *Completion rates (session/module)* | Ongoing | Not reported | 0.5 (Not reported) | No significant differences between groups in average number of treatment sessions. |
|  |  | *Feedback measures* | Qualitative | Not reported | 0.5 (Not reported) | No significant differences between groups in average overall treatment time in minutes. |
|  |  | *Feedback measures* | Qualitative | F(1, 63) = 4.00, p = .05 | p = .05 | Higher ratings for mothers and fathers in the AccessEI group when compared with the face-to-face group. |
|  |  | *Feedback measures* | Qualitative | F(1, 42) = 3.99, p = .05 | p = .05 |  |
|  |  | *Satisfaction measures* | Qualitative | Not reported | 0.5 (Not reported) | No differences between treatment conditions on satisfaction ratings |
|  |  | *Satisfaction measures* | Qualitative | Not reported | 0.5 (Not reported) | No differences between treatment conditions on satisfaction ratings |
|  |  | *Retention/Attrition/Dropout (study)* | Ongoing |  |  |  |
| Day JJ, Sanders MR. (2018)* | Triple P Online (TPOL) | *Completion rates (specific component)* | Quality |  |  |  |
|  |  | *Time or duration (specific component)* | Quality |  |  |  |
|  |  | *Completion rates (session/module)* | Ongoing | Mann–Whitney U = 1,090, p<.001, r = .37 | p<.001 | Participants in the TPOLe condition completed significantly more modules than TPOL. |
|  |  | *Time or duration (session or module)* | Ongoing |  |  |  |
| Epstein M, Oesterle S, Haggerty KP. (2019) | Parenting in the Middle School Years (PIMSY) | *Completion rates (specific component)* | Quality |  |  |  |
|  |  | *Interviews* | Qualitative |  |  |  |
|  |  | *Completion rates (specific component)* | Quality | Not reported | 0.5 (Not reported) | |
|  |  | *Satisfaction measures* | Qualitative | p < .10 | p < .10 | Parents in the materials-only group were marginally more likely to say that they would recommend the program to other parents than parents in the Facebook group condition. |
| Jones DJ, Forehand R, Cuellar J, Parent J, Honeycutt A, Khavjou O, et al. (2014) | TE-HNC (Technology-enhanced Helping the Non Compliant Child) | *Satisfaction measures* | Qualitative | *d* = 1.48 | Not reported | Fewer sessions did not compromise family satisfaction with the TE-HNC relative to HNC program. Rather, between-group effect size analysis favoured TE-HNC. |
|  |  | *Retention/Attrition/Dropout (study)* | Ongoing |  |  |  |
|  |  | *Frequency (attendance)* | Ongoing | *d* = 0.88 | Not reported | Families in TE-HNC were more likely to attend weekly sessions than families in HNC. |
|  |  | *Completion rates (specific component- calls)* | Quality | *d* = 2.59 | Not reported | Families in TE-HNC were more likely to participate in midweek calls than families in HNC. |
|  |  | *Completion rates (specific component- homework)* | Quality | *d* = 0.63 | Not reported | Families in TE-HNC were more likely to complete their Child’s Game home practice than families in HNC. |
| Jones DJ, Loiselle R, Zachary C, Georgeson AR, Highlander A, Turner P, et al. (2021)* | TE-HNC (Technology-enhanced Helping the Non Compliant Child) | *Frequency (attendance)* | Ongoing | *d* = 0.3 | 0.5 (Not reported) | Average session attendance revealed that it was relatively high and similar (ES = 0.30) for both HNC and TE-HNC |
|  |  | *Completion rates (specific component)* | Quality | ES = 1.06; TE-HNC 85.6%; HNC 59.7%, p < .001, d = 1.06 | p < .001 | TE- HNC families had significantly better mid-week call participation |
|  |  | *Completion rates (specific component)* | Quality | ES = 0.56; TE-HNC 88.5%; HNC 59.7%, p < .05, d = 0.56 | p < .05 | TE- HNC families had significantly better homework compliance than HNC. |
|  |  | *Satisfaction measures* | Qualitative | *d* = 0.43 | 0.5 (Not reported) | There were no significant differences in program satisfaction or ease of use of skills |
| Kavanagh DJ, Connolly J, Fisher J, Halford WK, Hamilton K, Hides L, et al. (2021)* | Baby Steps Wellbeing | *Frequency (logins)* | Ongoing | t=-0.60, df=487.4, p=.550 | p=.550 | No substantial differences due to treatment; access and use were suboptimal. |
|  |  | *Time or duration (program)* | Ongoing | t=-0.24, df=488.4, p=.809 | p=.809 |  |
|  |  | *Intensity (core module interaction)* | Ongoing | t=-3.40, df=492, p=<.01 | p=<.01 | More Baby Care modules were viewed by the participants in the Baby Care treatment. |
|  |  | *Intensity (core module interaction)* | Ongoing | t=-0.33, df=488.8, p=.743 | p=.743 | No difference between treatments in total modules viewed. |
|  |  | *Satisfaction measures* | Qualitative | t=0.54, df=397.5, p=.592 | p=.592 | Satisfaction with the program was high among the participants who accessed it at least once, with no substantial differences due to treatment. |
| Kirkman JJL, Hawes DJ, Dadds MR. (2016)* | Integrated Family Intervention for Child Conduct Problems (BPT) | *Feedback measures* | Qualitative |  |  |  |
|  |  | *Completion rates (session/module)* | Ongoing |  |  |  |
|  |  | *Satisfaction measures* | Qualitative |  |  |  |
|  |  | *Frequency (logins)* | Ongoing |  |  |  |
|  |  | *Feedback measures* | Qualitative |  |  |  |
|  |  | *Feedback measures* | Qualitative |  |  |  |
|  |  | *Satisfaction measures* | Qualitative |  |  |  |
|  |  | *Feedback measures* | Qualitative |  |  |  |
|  |  | *Feedback measures* | Qualitative | t(33.48) = 1.37, p = .18 | p = .18 | There was no significant difference in parents’ rating of session engagement between clinic-based and e-health conditions. |
|  |  | *Feedback measures* | Qualitative | t(44) = 2.56, p = .03, d = .67 | p = .03 | Clinicians’ rating of parent engagement was significantly higher for clinic-based families compared to e-health families . |
|  |  | *Frequency (attendance)* | Ongoing | (M = .41, SD = .91), U = 131.00, z = –3.180, p = .001 | p = .001 | Clinic-based clients had significantly more cancellations than e-health clients. |
|  |  | *Feedback measures* | Qualitative |  |  |  |
|  |  | *Retention/Attrition/Dropout (intervention)* | Ongoing |  |  |  |
| Morgan AJ, Rapee RM, Bayer JK. (2016)* | Cool Little Kids Online | *Intensity (specific component usage)* | Quality |  |  |  |
|  |  | *Frequency (logins)* | Ongoing | t(45) = −1.62, p = 0.113, d = 0.51. | p = 0.113 | There were fewer logins in the unsupported group than the supported group and the difference was medium in size, though not statistically significant, |
|  |  | *Intensity (module interaction)* | Ongoing | t(34) = 0.42, p = 0.676, d = 0.15. | p = 0.676 | The number of self-reported modules completed by parents in the unsupported arm was not significantly different from parents in the supported arm. |
|  |  | *Completion rates (session/module)* | Ongoing |  |  |  |
|  |  | *Time or duration (session or module)* | | | |  |
|  |  | *Other: reasons for non-completion* | Qualitative |  |  |  |
|  |  | *Satisfaction measures* | Qualitative |  |  |  |
| Morgan AJ, Rapee RM, Salim A, Goharpey N, Tamir E, McLellan LF, et al. (2017) | Cool Little Kids | *Retention/Attrition/Dropout (study)* | Ongoing | Unadjusted: 1.79 (1.15–2.79), p = 0.010. Adjusted: 1.90 (1.18–3.07) p = 0.008. | p = 0.008 | The odds of completing the questionnaire were significantly higher in the informed group than the uninformed group. |
|  |  | *Frequency (logins)* | Ongoing |  |  |  |
|  |  | *Intensity (module interaction)* | Ongoing |  |  |  |
|  |  | *Completion rates (session/module)* | Ongoing |  |  |  |
|  |  | *Time or duration (program)* | Ongoing |  |  |  |
|  |  | *Other: reasons for non-completion* | Qualitative |  |  |  |
|  |  | *Satisfaction measures* | Qualitative |  |  |  |
| Murry VM, Berkel C, Liu N. (2018)* | Pathways for African American Success (PAAS) | *Enrolment rates* | Initial | R2 = .03, χ2(1) = 6.08, p <.01; (β = 0.62 p <.01) | p <.01 | Not reported |
|  |  | *Frequency (attendance)* | Ongoing | R2 = .09, F(1,277) = 27.00, p <.001; (β = 1.58 p <.001 | p <.001 | Attendance was significantly higher in the technology condition than in the group condition for all of the indicators. |
|  |  | *Satisfaction measures* | Qualitative |  |  |  |
|  |  | *Retention/Attrition/Dropout (study)* | Ongoing |  |  |  |
| Rabbitt SM, Carrubba E, Lecza B, McWhinney E, Pope J, Kazdin AE. (2016)* | Parent Management Training (PMT) | *Intensity (specific component usage)* | Quality | t(58) = 1.52, p = .13 | p = .13 | There were no differences between the groups in terms of telephone communication. |
|  |  | *Satisfaction measures* | Qualitative | Parent Alliance: t(58) \ 1, ns; Therapist Alliance: t(58) \1, ns. | 0.5 (Not reported) | Parents and therapists did not differ in their ratings of the therapeutic alliance for the Reduced and Full Contact treatment conditions. Interestingly, the alliance between the therapist and client did not vary as a function of frequent versus reduced contact with the therapist. |
|  |  | *Adherence* | Quality | t(57) = 1.61, p = .11 | p = .11 | Parents in the two treatment groups did not differ in their self-reported adherence to treatment. |
|  |  | *Adherence* | Quality | t(57) = 3.06, p = .003, d = 0.77 | p = .003 | Therapists rated parents in the Full Contact PMT as more adherent to treatment than parents in the Reduced Contact group , indicating that therapists perceived parents in the Full Contact group as using the skills more frequently and more consistently than parents in the Reduced Contact group. |
| Sanders MR, Dittman CK, Farruggia SP, Keown LJ. (2014)* | Triple P Online (TPOL) | *Satisfaction measures* | Qualitative | Mothers: t(172) \ 1.00, ns. Fathers: t(118) \ 1.00, ns. | 0.5 (Not reported) | No significant difference in levels of satisfaction with either program. |
|  |  | *Completion rates (session/module)* | Ongoing |  |  |  |
| Schramm DG, McCaulley G. (2012)* | Focus on Kids (Online) | *Satisfaction measures* | Qualitative | t=8.06 | p <.05 | No differences to questions relating to skills taught in program, but differences in actual satisfaction. |
| Stevens J, Scribano PV, Marshall J, Nadkarni R, Hayes J, Kelleher KJ. (2015) | Not reported | *Intensity (specific component usage)* | Quality |  |  |  |
|  |  | *Time or duration (specific component)* | Quality |  |  |  |
|  |  | *Enrolment rates* | Initial |  |  |  |
|  |  | *Retention/Attrition/Dropout (study and treatment)* | Ongoing | χ2 = 0.155, p = .92 | p = .92 | *There was no significant difference in retention between the two conditions.* |

* = study included in Stouffer’s *p* synthesis of associations between engagement strategies and engagement outcomes

## **Table 4.** Summary of Between-Group ACE Outcomes of Studies Included in Secondary Outcome

|  |  |  |  |  |  |  |
| --- | --- | --- | --- | --- | --- | --- |
| **Study** | **Program name** | **Target ACE** | **ACE Measure** | **ACE outcome** | **p-value** | **Interpretation comments** |
| Agazzi H, Hayford H, Thomas N, Ortiz C, Salinas-Miranda A. A (2021) | iHOT-DOCTS (BPT) | Maladaptive parenting | Parenting Stress- DOCS PSM, adapted from the Autism Parenting Stress Index.^1^ | p=.17 | p=.17 | Post-test score differences for child behaviour and caregiver stress were not significantly different across type of class attended. |
| Antonini TN, Raj SP, Oberjohn KS, Cassedy A, Makoroff KL, Fouladi M, et al. (2014)* | i-INTERACT | Maladaptive parenting | Dyadic Parent–Child Interaction  Coding System (DPICS)^2^ | | | |
|  |  |  | DPICS: positive parenting behaviours | Low income group: RR = 14.07, p < .0001; Higher- income group: RR = 3.29, p = .0001. | p = .0001 | Parents in both lower and higher income intervention more likely to provide pos behaviours than IRC group. |
|  |  |  | DPICS: undesirable parenting behaviours | RR = 2.32, p = .0002 | p = .0002 | Parents in both groups were significantly less likely to provide undesirable statements post-intervention |
|  |  |  | DPICS: parent praise | RR = 15.37, p = .009 | p = .009 | Parents in the I-InTERACT groups had a higher percentage of labelled praises following compliance than parents in the IRC groups |
|  |  |  | DPICS: parent response | Not reported | p>0.5 (Not reported) | No parent in either group at any visit provided clear consequences. |
|  |  |  | DPICS: parent praise in child-directed play x Total sessions | r = .48, p = .05 | p = .05 | Number of sessions that parents in the I-InTERACT groups completed was positively associated with the total number of labeled praises provided during child directed play. |
|  |  |  | DPICS: parent praise in child-directed play x Total sessions | r = .43, p = .09 | p = .09 | Number of sessions that parents in the I-InTERACT groups completed was positively associated with the total number of labelled praises provided during parent directed play. |
| Breaux R, Shroff DM, Cash AR, Swanson CS, Carlton C, Bertollo JR, et al. (2021) | RELAX | Maladaptive parenting | Difficulties in Emotion Regulation Scale^3^ | η2 = .03 | Not reported | Small but non-significant improvements in regulating were found across both groups. |
|  |  |  | Coping with Children’s Negative Emotions^4^ | Fs = 0.20−1.85, ps > .185 | p > .185 | Parent non-supportive reactions were not significantly different across the in-person and telehealth groups. |
|  |  |  | Conflict Behavior Questionnaire^5^ | η2 = .013 | p>0.5 (Not reported) | Caregiver- and adolescent-reports of caregiver-adolescent conflict did not improve significantly across the entire sample for global pre/post measures. |
|  |  |  | Conflict Behavior Questionnaire^5^ | η2 = .003 | p>0.5 (Not reported) | Caregiver-report of caregiver-adolescent conflict from pre- to post- treatment was significantly different across the in- person and telehealth groups (with statistically significant improvements in the in-person group but not the telehealth group). |
| Breitenstein SM, Fogg L, Ocampo EV, Acosta DI, Gross D. (2016) | ezParent (adapted from Chicago Parent Program) | Maladaptive parenting | Parenting Questionnaire^6^: Warmth | F1,77 = 4.82, P<.05 | p<.05 | From T1 to T3, there was a significant difference between conditions for parenting warmth. |
|  |  |  | Parenting Questionnaire^6^: Follow through of discipline | Not reported | p>0.5 (Not reported) | There were no significant differences between the intervention and control conditions on parents’ reports of their follow through on discipline. |
|  |  |  | Parenting Questionnaire^6^: Corporal punishment | Not reported | p>0.5 (Not reported) | There were no significant differences between the intervention and control conditions on parents’ reports of their use of corporal punishment. |
|  |  |  | Parenting Stress Index^7^ - Short Form | Not reported | p>0.5 (Not reported) | There were no significant differences between the intervention and control conditions on parents’ reports of parenting stress. |
| Cefai J, Smith D, Pushak RE. (2010) | Parenting Wisely | Maladaptive parenting | No ACE measure administered in this study. | | | |
| Comer JS, Furr JM, Miguel EM, Cooper-Vince CE, Carpenter AL, Elkins RM, et al. (2017) | Internet Parent Child Interaction Therapy (I-PCIT) | Maladaptive parenting | None administered in this study. | | |  |
| Czymoniewicz-Klippel M, Chesnut R, DiNallo J, Perkins D. (2019) | Grow Online | Maladaptive parenting | None administered in this study. | | |  |
| Dadds MR, Thai C, Mendoza Diaz A, Broderick J, Moul C, Tully LA, et al. (2019) | AccessEI | Maladaptive parenting | None administered in Study A nor Study B. | | |  |
|  |  |  |  |  |  |  |
| Day JJ, Sanders MR. (2018) | Triple P Online (TPOL) | Maladaptive parenting | Parenting Scale^8^: T1 Total | P=.151, d= 0.36 | p=.151 | Both intervention conditions showed significant pre–post change in terms of parenting practices based on PS Total scores. |
|  |  | Maladaptive parenting | Parenting Scale^8^: T3 Total | Bdiff = –0.30, t(243.99) = –2.27, p =.024, d = 0.70. | p =.024 | Participants in the TPOLe condition also showed significantly greater reduction in negative parenting practices at T3 relative to TPOL based on PS Total scores. |
|  |  | Maladaptive parenting | Parental Anger Inventory^9^: T1 | Not reported | p>0.5 (Not reported) | Short-term reductions in the frequency of anger responses (PAI Problem subscale) were found for both conditions. |
| Epstein M, Oesterle S, Haggerty KP. (2019) | Parenting in the Middle School Years (PIMSY) | Maladaptive parenting | Study-specific (measures monitoring, conflict, rewards and consistent discipline). | *p* < .10 | *p* < .10 | Parents in the materials-only group reported a marginal increase in rewards whereas the Facebook group means slightly declined from baseline to 6-month follow-up. |
| Jones DJ, Forehand R, Cuellar J, Parent J, Honeycutt A, Khavjou O, et al. (2014) | TE-HNC (Technology-enhanced Helping the Non Compliant Child) | Maladaptive parenting | None administered in this study. | | |  |
| Jones DJ, Loiselle R, Zachary C, Georgeson AR, Highlander A, Turner P, et al. (2021) | TE-HNC (Technology-enhanced Helping the Non Compliant Child) | Maladaptive parenting | None administered in this study. | | |  |
| Kavanagh DJ, Connolly J, Fisher J, Halford WK, Hamilton K, Hides L, et al. (2021) | Baby Steps Wellbeing | Maladaptive parenting | Relationship satisfaction (CSI-16c)^10^ | t= 2.13(880.7), p= .03 | p= .03 | There was a reduction in relationship satisfaction but this was significantly less for Baby Steps Wellbeing than in Baby Care |
| Kirkman JJL, Hawes DJ, Dadds MR. (2016) | Integrated Family Intervention for Child Conduct Problems (BPT) | Maladaptive parenting | Alabama Parenting Questionnaire Short Form^11^ | F(4, 31) = 1.03, p = .408 | p = .408 | There was no multivariate effect of condition. |
| Morgan AJ, Rapee RM, Bayer JK. (2016) | Cool Little Kids Online | Maladaptive parenting | Overinvolved/Protective Parenting Scale^8^ | F(1.37.6) = 0.45, p = 0.508 | p = 0.508 | The difference between the clinician supported and unsupported groups in the amount of change over time was not significant for any of the secondary outcomes, and the pattern of effect did not consistently favour either group. |
| Morgan AJ, Rapee RM, Salim A, Goharpey N, Tamir E, McLellan LF, et al. (2017) | Cool Little Kids | Maladaptive parenting | Overinvolved/Protective Parenting Scale^8^ | p = .217 | p = .217 | The intervention and control groups did not differ at follow-up on overprotective parenting. Both of these outcomes reduced substantially from baseline to follow-up across trial arms. |
| Murry VM, Berkel C, Liu N. (2018) | Pathways for African American Success (PAAS) | Maladaptive parenting | None administered in this study. | | |  |
| Rabbitt SM, Carrubba E, Lecza B, McWhinney E, Pope J, Kazdin AE. (2016) | Parent Management Training (PMT) | Maladaptive parenting | Parenting Stress Index^7^ | F=2.29 | p>0.5 (Not reported) | *None of the treatment group x time interactions was significant, indicating that patterns of change in parent and family functioning were similar across the two treatment groups.* |
|  |  |  | Management of Children’s Behavior Scale^12^ | [F(1,57) \ 1, ns] | p>0.5 (Not reported) | *Parents in both the Full Contact [t(30) = 4.05, p \.001, d = 0.73] and Reduced Contact [t(28) = 4.70, p \ .001, d = 0.88] groups demonstrated significant improvement in parent practices over the course of treatment. There was no significant difference between the groups on their posttreatment MCBS score after controlling for pre-treatment score [F(1,57) \ 1, ns], suggesting that the pattern of change over time did not differ between the two groups.* |
|  |  |  | Family Environment Scale^13^ | F=0.13 | p>0.5 (Not reported) | *None of the treatment group x time interactions was significant, indicating that patterns of change in parent and family functioning were similar across the two treatment groups.* |
| Sanders MR, Dittman CK, Farruggia SP, Keown LJ. (2014)* | Triple P Online (TPOL) | Maladaptive parenting | Parenting Scale^8^ -Overreactivity | Mother: *d* = -.03; Fathers: d = 0.05. | | TPOL produced short-term intervention effects for child behaviour and dysfunctional parenting that were noninferior to the effects of SHTP. |
|  |  |  | Parenting Scale^8^ - Total x Session completion (Mothers) | β = -.42, SE = 0.03, p < 0.01 | p < 0.01 | Session completion was a significant independent predictor of mothers’ ineffective parenting. |
|  |  |  | Parenting Scale^8^ - Total x Session completion (Fathers) | β = -.22, SE = 0.03, p > .05 | p > .05 | Session completion was not a significant independent predictor of fathers’ ineffective parenting. |
| Schramm DG, McCaulley G. (2012) | Focus on Kids (Online) | Interparental conflict | Study-specific: retrospective rating of understanding of the below statements before and after intervention. | | | FTF participants reporting a significantly greater increase in understanding than participants who completed the course online |
|  |  | Interparental conflict | How children are affected | F(1, 1191) = 7.58, p = .006; partial η2 = .006, | p = .006 |  |
|  |  | Interparental conflict | Parent involvement | F(1, 1285) = 9.35, p = .002; partial η2 = .007 | p = .002 |  |
|  |  | Interparental conflict | Parent cooperation | F(1, 1283) = 3.85, p =.05; partial η2 = .003 | p =.05 |  |
|  |  | Interparental conflict | Different child needs | Not reported | |  |
| Stevens J, Scribano PV, Marshall J, Nadkarni R, Hayes J, Kelleher KJ. (2015) | Not reported | Interparental conflict | Composite Abuse Scale.^14^ | M=9.8, SD= 21.8 (intervention) M= 8.7, SD= 13.7 (control), p > .05 | p > .05 | No group differences in IPV victimization or any health outcomes at either time point. |

* = study included in Stouffer’s *p* synthesis of associations between engagement outcomes and ACE outcomes.

*Note.* References cited for each ACE measure.

^1^Silva, L. M., & Schalock, M. (2012). Autism parenting stress index: Initial psychometric evidence. Journal of Autism and Developmental Disorders, 42(4), 566-574. doi:10.1007/s10803-011-1274-1

^2^Eyberg, S. M., Nelson, M. M., Duke, M., & Boggs, S. R. (2005). Manual for the Dyadic Parent–Child Interaction Coding System (3rd ed.). Gainesville: University of Florida.

^3^Gratz, K. L., & Roemer, L. (2004). Multidimensional assessment of emotion regulation and dysregulation: Development, factor structure, and initial validation of the difficulties in emotion regulation scale. Journal of Psychopathology and Behavioral Assessment, 26(1), 41–54. https://doi.org/10.1023/B:JOBA.0000007455.08539.94

^4^Fabes, R. A., Eisenberg, N., & Bernzweig, J. (1990). The Coping with Children’s Negative Emotions Scale: Procedures and scoring. Arizona State University.

^5^Prinz, R. J., Foster, S., Kent, R. N., & O’Leary, K. D. (1979). Multivariate assessment of conflict in distressed and non-distressed mother-adolescent dyads. Journal of Applied Behavior Analysis, 12(4), 691–700. https://doi.org/10.1901/ jaba.1979.12-691

^6^Gross D, Fogg L, Garvey C, Julion W. Behavior problems in young children: an analysis of cross-informant agreements and disagreements. Res Nurs Health 2004;27:413-425. [doi: 10.1002/nur.20040] [Medline: 15514961]

^7^Abidin, R.R. (1990). Parenting Stress Index (PSI). Charlottesville, VA: Pediatric Psychology Press; Lloyd, B. H., & Abidin, R. R. (1985). Revision of the parenting stress index. Journal of Pediatric Psychology, 10, 169–177.

^8^Arnold, D. S., O’Leary, S. G., Wolff, L. S., & Acker, M. M. (1993). The Parenting Scale: A measure of dysfunctional parenting in discipline situations. Psychological Assessment, 5 (2), 137–144. Retrieved from http://psycnet.apa.orgjournals/ pas/5/2/137

^9^Hansen, D. J., & Sedlar, G. (1998). Manual for the PAI: The Parental Anger Inventory. Lincoln: University of Nebraska, Clinical Psychology Training Program

^10^Funk JL, Rogge RD. Testing the ruler with item response theory: increasing precision of measurement for relationship satisfaction with the Couples Satisfaction Index. J Fam Psychol 2007 Dec;21(4):572-583. [doi: 10.1037/0893-3200.21.4.572] [Medline: 18179329]

^11^Elgar, F. J., Waschbusch, D. A., Dadds, M. R., & Sigvaldason, N. (2007). Development and validation of a short form of the Alabama Parenting Questionnaire. Journal of Child and Family Studies, 16, 243–259. doi:10.1007/s10826-0069082-5

^12^Perepletchikova, F., & Kazdin, A. E. (2004). Assessment of parenting practices related to conduct problems: Development and validation of the management of children’s behavior scale. Journal of Child and Family Studies, 13, 385–403.

^13^Moos, R. H., & Moos, B. S. (1981). Family environment scale manual. Palo Alto: Consulting Psychologists Press.

^14^Hegarty, K., Bush, R., & Sheehan, M. (2005). The Composite Abuse Scale: Further development and assessment of reliability and validity of a multidimensional partner abuse measure in clinical settings. Violence and Victims, 20, 529-547.
