# Supplementary material for: Engaging Parents in Technology-Assisted Interventions for Childhood Adversity: Systematic Review
Source: J Med Internet Res. 2024 Jan 19;26:e43994. doi: 10.2196/43994 (PMC10837762; doi:10.2196/43994)
Supplement: Multimedia Appendix 5 [file jmir_v26i1e43994_app5.docx]

# Appendix 5

# Quality Assessment of Included Studies: Full Summary of Results and Results from Each Included Study

## **Table S1.** Summary of Quality Assessment Outcomes in Included Studies

|  |  |  |  |  |  |  |  |
| --- | --- | --- | --- | --- | --- | --- | --- |
| **Category of study designs** | **Mixed Methods Appraisal Tool (MMAT) methodological quality criteria** | **Ratings** | | | | |  |
|  |  | **Yes** | | **No** | | **Can't tell** | |
|  |  | *n* | **%** | *n* | **%** | *n* | **%** |
| **Screening questions** | S1. Are there clear research questions? | 122 |  | 5 |  | 0 | 0 |
|  | S2. Do the collected data allow to address the research questions? | 123 |  | 4 |  | 0 | 0 |
| **Qualitative studies** | |  |  |  |  |  |  |
| *n = 3* | 1.1. Is the qualitative approach appropriate to answer the research question | 3 | 100 | 0 | 0 | 0 | 0 |
|  | 1.2. Are the qualitative data collection methods adequate to address the research question? | 3 | 100 | 0 | 0 | 0 | 0 |
|  | 1.3. Are the findings adequately derived from the data? | 3 | 100 | 0 | 0 | 0 | 0 |
|  | 1.4. Is the interpretation of results sufficiently substantiated by data? | 3 | 100 | 0 | 0 | 0 | 0 |
|  | 1.5. Is there coherence between qualitative data sources, collection, analysis and interpretation? | 3 | 100 | 0 | 0 | 0 | 0 |
|  | *All criteria met Yes* | *3* | *100* |  |  |  |  |
| **Quantitative randomised controlled trial studies** | | | | |  |  |  |
| *n = 77* | 2.1. Is randomization appropriately performed? | 50 | 64.94 | 4 | 5.19 | 23 | 29.87 |
|  | 2.2. Are the groups comparable at baseline? | 61 | 79.22 | 7 | 9.09 | 9 | 11.69 |
|  | 2.3. Are there complete outcome data? | 42 | 54.55 | 28 | 36.36 | 7 | 9.09 |
|  | 2.4. Are outcome assessors blinded to the intervention provided? | 42 | 54.55 | 8 | 10.39 | 27 | 35.06 |
|  | 2.5 Did the participants adhere to the assigned intervention? | 32 | 41.56 | 24 | 31.17 | 21 | 27.27 |
|  | *All criteria met Yes* | *8* | *10.39* |  |  |  |  |
| **Quantitative non-randomised studies** | | | |  |  |  |  |
| *n = 31* | 3.1. Are the participants representative of the target population? | 24 | 77.42 | 4 | 12.90 | 3 | 9.68 |
|  | 3.2. Are measurements appropriate regarding both the outcome and intervention (or exposure)? | 24 | 77.42 | 3 | 9.68 | 4 | 12.90 |
|  | 3.3. Are there complete outcome data? | 12 | 38.71 | 16 | 51.61 | 3 | 9.68 |
|  | 3.4. Are the confounders accounted for in the design and analysis? | 16 | 51.61 | 13 | 41.94 | 2 | 6.45 |
|  | 3.5. During the study period, is the intervention administered (or exposure occurred) as intended? | 21 | 67.74 | 2 | 6.45 | 8 | 25.81 |
|  | *All criteria met Yes* | *6* | *19.35* |  |  |  |  |
| **Quantitative descriptive studies** | | |  |  |  |  |  |
| *n = 5* | 4.1. Is the sampling strategy relevant to address the research question? | 4 | 80.00 | 1 | 20.00 | 0 | 0.00 |
|  | 4.2. Is the sample representative of the target population? | 2 | 40.00 | 3 | 60.00 | 0 | 0.00 |
|  | 4.3. Are the measurements appropriate? | 3 | 60.00 | 0 | 0.00 | 2 | 40.00 |
|  | 4.4. Is the risk of nonresponse bias low? | 1 | 20.00 | 2 | 40.00 | 2 | 40.00 |
|  | 4.5. Is the statistical analysis appropriate to answer the research question? | 4 | 80.00 | 1 | 20.00 | 0 | 0.00 |
|  | *All criteria met Yes* | *1* | *20.00* |  |  |  |  |
| **Mixed method studies** | |  |  |  |  |  |  |
| *n = 6* | 5.1. Is there an adequate rationale for using a mixed methods design to address the research question? | 5 | 83.33 | 1 | 16.67 | 0 | 0.00 |
|  | 5.2. Are the different components of the study effectively integrated to answer the research question? | 4 | 66.67 | 2 | 33.33 | 0 | 0.00 |
|  | 5.3. Are the outputs of the integration of qualitative and quantitative components adequately interpreted? | 4 | 66.67 | 2 | 33.33 | 0 | 0.00 |
|  | 5.4. Are divergences and inconsistencies between quantitative and qualitative results adequately addressed? | 4 | 66.67 | 1 | 16.67 | 1 | 16.67 |
|  | 5.5. Do the different components of the study adhere to the quality criteria of each tradition of the methods involved? | 5 | 83.33 | 1 | 16.67 | 0 | 0.00 |
|  | *All criteria met Yes* | *3* | *50.00* |  |  |  |  |

## **Table 2.** Quality Assessment Outcomes of Studies Included in Primary Outcome

|  |  |  |  |  |  |  |
| --- | --- | --- | --- | --- | --- | --- |
| Study type | **Study** | **MMAT Items** | | | | |
|  |  | **MMAT screening item 1** | **MMAT screening item 2** |  |  |  |
|  |  | S1. Are there clear research questions? | S2. Do the collected data allow to address the research questions? |  |  |  |
| *Qualitative study* | Franke N, Keown LJ, Sanders MR. (2020) | No | Yes |  |  |  |
| *Descriptive* | Loew B, Rhoades G, Markman H, Stanley S, Pacifici C, White L, et al. (2012) | No | No |  |  |  |
| *Quantitative descriptive* | May CD, St George JM, Lane S. (2021) | No | No |  |  |  |
| *Overview paper* | Thomson M. (2011) | No | No |  |  |  |
| *Quantitative randomised controlled trial studies* |  |  |  |  |  |  |
|  |  | **MMAT Item 1** | **MMAT Item 2** | **MMAT Item 3** | **MMAT Item 4** | **MMAT Item 5** |
|  |  | 2.1. Is randomization appropriately performed? | 2.2. Are the groups comparable at baseline? | 2.3. Are there complete outcome data? | 2.4. Are outcome assessors blinded to the intervention provided? | 2.5 Did the participants adhere to the assigned intervention? |
|  | Antonini TN, Raj SP, Oberjohn KS, Cassedy A, Makoroff KL, Fouladi M, et al. (2014)* | Yes | Yes | Yes | Yes | Yes |
|  | Baggett K, Davis B, Feil E, Sheeber L, Landry S, Leve C, et al. (2017) | Yes | Yes | Yes | Yes | Yes |
|  | Baggett KM, Davis B, Feil EG, Sheeber LL, Landry SH, Carta JJ, et al. (2010) | Yes | Can't tell | Yes | Can't tell | Yes |
|  | Baggett KM, Davis B, Sheeber LB, Ammerman RT, Mosley EA, Miller K, et al. (2020) | Can't tell | Yes | Can't tell | Can't tell | Yes |
|  | Baker M, Biringen Z, Meyer-Parsons B, Schneider A. (2020) | Can't tell | Yes | Yes | Can't tell | Yes |
|  | Baker S, Sanders MR, Turner KMT, Morawska A. A (2017) | Yes | Yes | Yes | No | No |
|  | Bodenmann G, Hilpert P, Nussbeck FW, Bradbury TN. (2014) | Can't tell | Yes | No | Yes | Can't tell |
|  | Boekhorst MG, Hulsbosch LP, Nyklicek I, Spek V, Kastelein A, Bogels S, et al. (2021) | Can't tell | Yes | Yes | Yes | No |
|  | Breitenstein SM, Fehrenbacher C, Holod AF, Schoeny ME. (2021) | Yes | Can't tell | Yes | Yes | No |
|  | Breitenstein SM, Fogg L, Ocampo EV, Acosta DI, Gross D. (2016)* | Yes | Yes | Yes | Can't tell | Yes |
|  | Cardamone-Breen MC, Jorm AF, Lawrence KA, Rapee RM, Mackinnon AJ, Yap MBH. (2018) | Yes | Yes | Yes | Yes | Yes |
|  | Cefai J, Smith D, Pushak RE. (2010)* | Can't tell | No | Yes | Can't tell | Yes |
|  | Choi H, Kim S, Ko H, Kim Y, Park CG. (2016) | Yes | Yes | No | Yes | No |
|  | Comer JS, Furr JM, Miguel EM, Cooper-Vince CE, Carpenter AL, Elkins RM, et al. (2017)* | Can't tell | Yes | No | Yes | Yes |
|  | Dadds MR, Thai C, Mendoza Diaz A, Broderick J, Moul C, Tully LA, et al. (2019)* *Study A* | Yes | Yes | Yes | No | Yes |
|  | Dadds MR, Thai C, Mendoza Diaz A, Broderick J, Moul C, Tully LA, et al. (2019)* *Study B* | Yes | Yes | Yes | Yes | Yes |
|  | Day JJ, Sanders MR. (2018)* | Yes | Yes | No | No | No |
|  | DeGarmo DS, Jones JA. (2019) | Can't tell | No | No | Can't tell | No |
|  | Donovan CL, March S. (2014) | Yes | Yes | No | Yes | No |
|  | Doss BD, Roddy MK, Llabre MM, Georgia Salivar E, Jensen-Doss A. (2020) | Yes | Yes | Can't tell | Yes | Can't tell |
|  | Ehrensaft MK, Knous-Westfall HM, Alonso TL. (2016) | Can't tell | Yes | No | Can't tell | No |
|  | Enebrink P, Högström J, Forster M, Ghaderi A. (2012). | Yes | Yes | No | Yes | Yes |
|  | Epstein M, Oesterle S, Haggerty KP. (2019) | Can't tell | Can't tell | Yes | Yes | Can't tell |
|  | Farris JR, Bert SSC, Nicholson JS, Glass K, Borkowski JG. (2013) | Yes | Yes | No | Can't tell | No |
|  | Feil EG, Baggett K, Davis B, Landry S, Sheeber L, Leve C, et al. (2020) | Can't tell | Yes | Yes | Yes | Yes |
|  | Ford-Gilboe M, Varcoe C, Scott-Storey K, Perrin N, Wuest J, Wathen CN, et al. (2020) | Yes | Yes | Yes | Yes | Can't tell |
|  | Franke N, Keown LJ, Sanders MR. (2020) | Can't tell | Yes | No | Can't tell | Yes |
|  | Gelatt VA, Adler-Baeder F, Seeley JR. (2010) | Can't tell | Yes | Yes | Yes | Yes |
|  | Ghaderi A, Kadesjo C, Bjornsdotter A, Enebrink P. (2018) | Yes | Yes | No | Yes | No |
|  | Hegarty K, Tarzia L, Valpied J, Murray E, Humphreys C, Taft A, et al. (2019) | Yes | Yes | Yes | Yes | Yes |
|  | Hemdi A, Daley D. (2017) | Yes | Yes | Yes | Yes | Can't tell |
|  | Hinton S, Sheffield J, Sanders MR, Sofronoff K. (2017) | Yes | Yes | No | No | Yes |
|  | Holden GW, Brown AS, Baldwin AS, Croft Caderao K. (2014) | Can't tell | No | Yes | Can't tell | Yes |
|  | Hudson DB, Campbell-Grossman C, Hertzog M. (2012) | Yes | No | Yes | Can't tell | Can't tell |
|  | Irvine AB, Gelatt VA, Hammond M, Seeley JR. (2015) | Can't tell | Yes | Yes | Yes | Can't tell |
|  | Jones DJ, Forehand R, Cuellar J, Parent J, Honeycutt A, Khavjou O, et al. (2014)* | Can't tell | Yes | No | Can't tell | Yes |
|  | Jones DJ, Loiselle R, Zachary C, Georgeson AR, Highlander A, Turner P, et al. (2021)* | Yes | Yes | Yes | Yes | Yes |
|  | Jones S, Calam R, Sanders M, Diggle PJ, Dempsey R, Sadhnani V. (2014) | Yes | Yes | No | Yes | Can't tell |
|  | Kaplan K, Solomon P, Salzer MS, Brusilovskiy E. (2014) | Yes | Yes | No | Can't tell | No |
|  | Kavanagh DJ, Connolly J, Fisher J, Halford WK, Hamilton K, Hides L, et al. (2021)* | Yes | Yes | Yes | Yes | No |
|  | Koziol-McLain J, Vandal AC, Wilson D, Nada-Raja S, Dobbs T, McLean C, et al. (2018) | Yes | Yes | Yes | Yes | Can't tell |
|  | Gulirmak K, Orak OS. (2020) | Yes | Yes | Yes | Can’t tell | Yes |
|  | Loew B, Rhoades G, Markman H, Stanley S, Pacifici C, White L, et al. (2012) | Can't tell | Yes | No | Can't tell | Yes |
|  | Mast JE, Antonini TN, Raj SP, Oberjohn KS, Cassedy A, Makoroff KL, et al. (2014) | Can't tell | Yes | Yes | No | Yes |
|  | Mello MJ, Bromberg JR, Baird J, Wills H, Gaines BA, Lapidus G, et al. (2019) | Yes | Yes | Yes | Yes | No |
|  | Morawska A, Tometzki H, Sanders MR. (2014) | No | Yes | Can't tell | Yes | Can't tell |
|  | Morgan AJ, Rapee RM, Bayer JK. (2016)* | Yes | No | No | Yes | No |
|  | Morgan AJ, Rapee RM, Salim A, Goharpey N, Tamir E, McLellan LF, et al. (2017) | Yes | Yes | No | Yes | No |
|  | Murry VM, Berkel C, Liu N. (2018)* | Can't tell | Can't tell | No | Yes | No |
|  | Narad ME, Minich N, Taylor HG, Kirkwood MW, Brown TM, Stancin T, et al. (2015) | Can't tell | Yes | Can't tell | Yes | Can't tell |
|  | Nickerson AB, Livingston JA, Kamper-DeMarco K. (2018) | Yes | Yes | Yes | Yes | Yes |
|  | O’Shea A, Kaplan K, Solomon P, Salzer MS. (2019) | Yes | Yes | No | Yes | No |
|  | Olthuis JV, McGrath PJ, Cunningham CE, Boyle MH, Lingley-Pottie P, Reid GJ, et al. (2018) | Yes | Yes | Can't tell | Can't tell | Can't tell |
|  | Porzig-Drummond R, Stevenson RJ, Stevenson C. (2015) | Yes | Yes | No | Can't tell | Yes |
|  | Potharst ES, Boekhorst MGBM, Cuijlits I, van Broekhoven KEM, Jacobs A, Spek V, et al. (2019) | Can't tell | Yes | Yes | Can't tell | No |
|  | Prinz RJ, Metzler CW, Sanders MR, Rusby JC, Cai C. (2021) | Yes | Yes | Yes | Yes | No |
|  | Rabbitt SM, Carrubba E, Lecza B, McWhinney E, Pope J, Kazdin AE. (2016)* | No | Yes | No | No | Can't tell |
|  | Raj SP, Shultz EL, Zang H, Zhang N, Kirkwood MW, Taylor HG, et al. (2018) | Yes | Yes | No | Can't tell | Can't tell |
|  | Razuri EB, Hiles Howard AR, Parris SR, Call CD, DeLuna JH, Hall JS, et al. (2016) | No | Yes | Yes | Can't tell | Can't tell |
|  | Richardson HL. (2021) | No | Yes | Yes | Can't tell | Can't tell |
|  | Rizzo CJ, Houck C, Barker D, Collibee C, Hood E, Bala K. (2020) | Yes | Yes | Yes | Can't tell | Yes |
|  | Rudd BN, Holtzworth-Munroe A, Reyome JG, Applegate AG, D’Onofrio BM. (2015) | Yes | No | Yes | Can’t tell | Can't tell |
|  | Sanders MR, Baker S, Turner KMT. (2012) | Yes | Yes | Yes | Can't tell | No |
|  | Sanders MR, Dittman CK, Farruggia SP, Keown LJ. (2014)* | Yes | Yes | Yes | Yes | No |
|  | Sawyer A, Kaim A, Le HN, McDonald D, Mittinty M, Lynch J, et al. (2019) | Yes | No | No | Yes | Can't tell |
|  | Scholer SJ, Hudnut-Beumler J, Dietrich MS. (2010) | Yes | Yes | Yes | No | Yes |
|  | Sheeber LB, Seeley JR, Feil EG, Davis B, Sorensen E, Kosty DB, et al. (2012) | Yes | Yes | Yes | Yes | Yes |
|  | Sim WH, Fernando LMN, Jorm AF, Rapee RM, Lawrence KA, Mackinnon AJ, et al. (2020) | Yes | Can't tell | Yes | Yes | No |
|  | Sourander A, McGrath PJ, Ristkari T, Cunningham C, Huttunen J, Lingley-Pottie P, et al. (2016) | Yes | Yes | Yes | Yes | Can't tell |
|  | Stevens J, Scribano PV, Marshall J, Nadkarni R, Hayes J, Kelleher KJ. (2015) | Yes | Yes | No | Can't tell | No |
|  | Sung JY, Mumper E, Schleider JL. (2021) | Yes | Can't tell | No | Yes | Yes |
|  | Taylor LC, Leary KA, Boyle AE, Bigelow KE, Henry T, DeRosier M. (2015) | Can't tell | Can't tell | Can't tell | No | Can't tell |
|  | Tiwari A, Yuk H, Pang P, Fong DYT, Yuen F, Humphreys J, et al. (2012) | Can't tell | Yes | Can't tell | Can't tell | Can't tell |
|  | Wade SL, Cassedy AE, Shultz EL, Zang H, Zhang N, Kirkwood MW, et al. (2017) | Yes | Yes | No | Yes | Yes |
|  | White L, Delaney R, Pacifici C, Nelson C, Dickinson SL, Golzarri-Arroyo L. (2019) | Can't tell | Can't tell | No | Can't tell | Yes |
|  | Yap MBH, Mahtani S, Rapee RM, Nicolas C, Lawrence KA, Mackinnon A, et al. (2018) | Yes | Yes | Yes | Yes | No |
|  | Zlotnick C, Tzilos Wernette G, Raker CA. (2019) | Yes | Can't tell | Yes | Yes | Yes |
| *Quantitative non-randomised studies* |  |  |  |  |  |  |
|  |  | 3.1. Are the participants representative of the target population? | 3.2. Are measurements appropriate regarding both the outcome and intervention (or exposure)? | 3.3. Are there complete outcome data? | 3.4. Are the confounders accounted for in the design and analysis? | 3.5. During the study period, is the intervention administered (or exposure occurred) as intended? |
|  | Agazzi H, Hayford H, Thomas N, Ortiz C, Salinas-Miranda A. A (2021)* | Yes | Yes | No | Yes | Yes |
|  | Becher EH, Cronin S, McCann E, Olson KA, Powell S, Marczak MS. (2015) | No | No | Yes | No | Can't tell |
|  | Bloom TL, Glass NE, Case J, Wright C, Nolte K, Parsons L. (2014) | Yes | Yes | No | Yes | Yes |
|  | Breaux R, Shroff DM, Cash AR, Swanson CS, Carlton C, Bertollo JR, et al. (2021)* | Yes | **Yes** | Yes | Yes | Yes |
|  | Brophy-Herb HE, Moyses K, Shrier C, Rymanowicz K, Pilkenton A, Dalimonte-Merckling D, et al. (2021) | No | Yes | No | No | Can't tell |
|  | Cotter KL, Bacallao M, Smokowski PR, Robertson CIB. (2013) | Yes | Yes | Can't tell | Can't tell | Yes |
|  | Czymoniewicz-Klippel M, Chesnut R, DiNallo J, Perkins D. (2019) | Yes | Can't tell | No | No | No |
|  | Schramm DG, McCaulley G. (2012)* | Yes | No | Yes | No | Can't tell |
|  | Ferraro AJ, Oehme K, Bruker M, Arpan L, Opel A. (2020) | Can't tell | Yes | Yes | No | Can't tell |
|  | Fleming GE, Kohlhoff J, Morgan S, Turnell A, Maiuolo M, Kimonis ER. (2021) | Yes | Yes | No | Yes | Yes |
|  | Fletcher R, Campbell L, Sved Williams A, Rawlinson C, Dye J, Baldwin A, et al. (2019) | Yes | Yes | Yes | Yes | Yes |
|  | Flujas-Contreras JM, Garcia-Palacios A, Gomez I. (2021) | No | Yes | Yes | No | Yes |
|  | H. Mohammadinasab, M. Mazaheri, M. Reazaeizade, M. Heydari. (2020) | No | Yes | Yes | No | Can't tell |
|  | Huebner DM, Rullo JE, Thoma BC, McGarrity LA, Mackenzie J. (2013) | Yes | Can't tell | No | No | Yes |
|  | James Riegler L, Raj SP, Moscato EL, Narad ME, Kincaid A, Wade SL. (2020) | Can't tell | Yes | No | Can't tell | Can't tell |
|  | Khor SPH, Fulgoni CM, Lewis D, Melvin GA, Jorm AF, Lawrence K, et al. (2021) | Yes | Yes | Yes | Yes | Yes |
|  | Kirkman JJL, Hawes DJ, Dadds MR. (2016)* | Yes | Yes | Yes | Yes | Yes |
|  | Kopystynska O, Turner JJ, Schramm DG, Higginbotham B. (2020) | Yes | Yes | No | No | Yes |
|  | Love SM, Sanders MR, Turner KMT, Maurange M, Knott T, Prinz R, et al. (2016) | Yes | Yes | No | Yes | Yes |
|  | May CD, St George JM, Lane S. (2021) | Yes | Yes | No | Yes | Yes |
|  | Owen DA, Hutchings J. (2017) | Yes | Can't tell | No | Yes | Yes |
|  | Perrino T, Estrada Y, Huang S, St George S, Pantin H, Cano MA, et al. (2018) | Yes | Yes | Can't tell | No | No |
|  | Piotrowska PJ, Tully LA, Collins DAJ, Sawrikar V, Hawes D, Kimonis ER, et al. (2020) | Yes | Yes | No | Yes | Yes? |
|  | Ristkari T, Kurki M, Suominen A, Gilbert S, Sinokki A, Kinnunen M, et al. (2019) | Yes | Yes | Can't tell | No | Yes |
|  | Self-Brown SR, C. Osborne M, Rostad W, Feil E. (2017) | Yes | Yes | No | No | Yes |
|  | Spence SH, Prosser SJ, March S, Donovan CL. (2020) | Yes | Yes | Yes | Yes | Can't tell |
|  | Stalker KC, Rose RA, Bacallao M, Smokowski PR. (2018) | Yes | Yes | Yes | Yes | Yes |
|  | Tully LA, Piotrowska PJ, Collins DAJ, Frick PJ, Anderson V, Moul C, et al. (2019) | Yes | Yes | Yes | Yes | Yes |
|  | Turner JJ, Kopystynska O, Bradford K, Schramm DG, Higginbotham BJ. (2021) | Can't tell | No | No | Yes? | Can't tell |
|  | van der Zanden RAP, Speetjens PAM, Arntz KSE, Onrust SA. (2010) | Yes | Yes | No | Yes | Yes |
|  | Yap MBH, Martin PD, Jorm AF. (2018) | Yes | Can't tell | No | No | Yes |
| *Quantitative descriptive studies* |  |  |  |  |  |  |
|  |  | 4.1. Is the sampling strategy relevant to address the research question? | 4.2. Is the sample representative of the target population? | 4.3. Are the measurements appropriate? | 4.4. Is the risk of nonresponse bias low? | 4.5. Is the statistical analysis appropriate to answer the research question? |
|  | Metcalfe RE, Matulis JM, Cheng Y, Stormshak EA. (2021) | Yes | Yes | Can't tell | Can't tell | Yes |
|  | Metzler CW, Sanders MR, Rusby JC, Crowley RN. (2012) | Yes | Yes | Yes | Yes | Yes |
|  | Russell BS, Maksut JL, Lincoln CR, Leland AJ. (2016) | Yes | No | Yes | No | Yes |
|  | Self-Brown S, Reuben K, Perry EW, Bullinger LR, Osborne MC, Bielecki J, et al. (2020) | No | No | Yes | Can't tell | Yes |
|  | Traube DE, Hsiao HY, Rau A, Hunt-O’Brien D, Lu L, Islam N. (2020) | Yes | No | Can't tell | No | No |
| *Qualitative studies* |  |  |  |  |  |  |
|  |  | 1.1. Is the qualitative approach appropriate to answer the research question | 1.2. Are the qualitative data collection methods adequate to address the research question? | 1.3. Are the findings adequately derived from the data? | 1.4. Is the interpretation of results sufficiently substantiated by data? | 1.5. Is there coherence between qualitative data sources, collection, analysis and interpretation? |
|  | Breitenstein SM, Shane J, Julion W, Gross D. Developing the eCPP: (2015) | Yes | Yes | Yes | Yes | Yes |
|  | Chu JTW, Wadham A, Jiang Y, Whittaker R, Stasiak K, Shepherd M, et al. (2019) | Yes | Yes | Yes | Yes | Yes |
|  | Fulgoni CMF, Melvin GA, Jorm AF, Lawrence KA, Yap MBH. (2019) | Yes | Yes | Yes | Yes | Yes |
| *Mixed method studies* |  |  |  |  |  |  |
|  |  | 5.1. Is there an adequate rationale for using a mixed methods design to address the research question? | 5.2. Are the different components of the study effectively integrated to answer the research question? | 5.3. Are the outputs of the integration of qualitative and quantitative components adequately interpreted? | 5.4. Are divergences and inconsistencies between quantitative and qualitative results adequately addressed? | 5.5. Do the different components of the study adhere to the quality criteria of each tradition of the methods involved? |
|  | Faulkner D, Hammond C, Nisbet L, Fletcher R. (2021) | Yes | No | No | No | Yes |
|  | Fletcher R, May C, Wroe J, Hall P, Cooke D, Rawlinson C, et al. (2016) | Yes | No | No | Can't tell | No |
|  | Hamil J, Gier E, Garfield CF, Tandon D. (2021) | Yes | Yes | Yes | Yes | Yes |
|  | Love SM, Sanders MR, Metzler CW, Prinz RJ, Kast EZ. (2013) | Yes | Yes | Yes | Yes | Yes |
|  | Ragavan MI, Ferre V, Bair-Merritt M. (2020) | No | Yes | Yes | Yes | Yes |
|  | Self-Brown S, Cowart-Osborne M, Baker E, Thomas A, Boyd C, Chege E, et al. (2015) | Yes | Yes | Yes | Yes | Yes |
|  |  |  |  |  |  |  |

* = study included in secondary outcome analysis
