# Supplementary material for: Engaging Parents in Technology-Assisted Interventions for Childhood Adversity: Systematic Review
Source: J Med Internet Res. 2024 Jan 19;26:e43994. doi: 10.2196/43994 (PMC10837762; doi:10.2196/43994)
Supplement: Multimedia Appendix 6 [file jmir_v26i1e43994_app6.docx]

# Appendix 6

# Primary Outcome Tables

| **Table S1.** Studies reporting a strategy used in the design phase | | | |  |
| --- | --- | --- | --- | --- |
| Type of strategy | Total observations | Studies reporting use | | |
|  | *N* | *n* | % | |
| Iterative approach | 3 | 3 | 7 | |
| Evidence-based approach | 1 | 1 | 2 | |
| Theory-based approach | 1 | 1 | 2 | |
| Partnerships | 2 | 2 | 5 | |
| Feedback | 3 | 2 | 5 | |
| End-user | 3 | 2 | 5 | |
| Stakeholder | 0 | 0 | 0 | |
| Expert | 0 | 0 | 0 | |
| Consultation | 53 | 35 | 81 | |
| End-user | 30 | 26 | 60 | |
| Stakeholder | 15 | 15 | 35 | |
| Expert | 7 | 6 | 14 | |
| Testing | 18 | 15 | 35 | |
| End-user | 16 | 15 | 35 | |
| Stakeholder | 2 | 2 | 5 | |
| Expert | 1 | 1 | 2 | |

| **Table S2.** Studies reporting a strategy used in the delivery phase | | | |
| --- | --- | --- | --- |
| Type of strategy | Total observations | Studies reporting use | |
|  | *N* | *n* | % |
| Targeted recruitment strategy | 26 | 25 | 22 |
| Practical support | 39 | 33 | 28 |
| **Content** |  |  |  |
| Behaviour change techniques | 75 | 49 | 42 |
| Action plans | 14 | 13 | 11 |
| Feedback | 25 | 24 | 20 |
| Goal setting | 22 | 22 | 19 |
| Self-monitoring | 14 | 13 | 11 |
| Reminders | 31 | 29 | 25 |
| Rewards | 10 | 10 | 9 |
| Social support features | 24 | 22 | 19 |
| Discussion forum* | 18 | 18 | 16 |
| Summaries | 7 | 6 | 5 |
| Supplemental resources | 7 | 7 | 6 |
|  |  |  |  |
| **Delivery** |  |  |  |
| Aesthetics | 6 | 6 | 5 |
| Control features | 102 | 65 | 56 |
| All-at-once | 4 | 4 | 3 |
| Reviewability | 35 | 27 | 23 |
| Tunnelling | 32 | 28 | 24 |
| Ease of use | 17 | 16 | 14 |
| Credibility | 14 | 11 | 9 |
| Guidance | 97 | 80 | 69 |
| Videos | 69 | 67 | 58 |
| Animation | 2 | 2 | 2 |
| Interactivity | 119 | 85 | 73 |
| Challenge | 43 | 42 | 36 |
| Gamification | 3 | 3 | 3 |
| Reflection | 18 | 17 | 15 |
| Rehearsal | 16 | 13 | 11 |
| Message tone | 10 | 10 | 9 |
| Mode | 23 | 23 | 25 |
| Narrative | 16 | 14 | 12 |
| Novelty | 5 | 4 | 3 |
| Personalisation | 11 | 10 | 9 |
| Tailoring | 18 | 17 | 11 |
| Professional support features | 60 | 54 | 47 |
| Clinical | 49 | 43 | 37 |
| Non-clinical | 11 | 11 | 9 |
|  |  |  |  |
| **Research only** |  |  |  |
| Reminders | 6 | 6 | 5 |
| Rewards/Incentives | 33 | 33 | 28 |

*All other studies’ social support features could not be categorised into one sub-category.

| **Table S3.** Measures used in included studies | | | |
| --- | --- | --- | --- |
| **Component of engagement**  Measure | Total observations | Studies reporting use | |
|  | *n* | *N* | % |
| **Initial engagement** |  | 83 |  |
| Expressions of interest | 9 | 9 | 11 |
| Recruitment rates | 52 | 52 | 63 |
| Enrolment rates | 61 | 61 | 73 |
|  |  |  |  |
| **Ongoing engagement** |  | 78 |  |
| Frequency |  |  |  |
| Attendance | 9 | 8 | 10 |
| Logins | 10 | 9 | 12 |
| Intensity |  |  |  |
| Session or module interaction | 8 | 7 | 9 |
| Completion rates |  |  |  |
| Program | 10 | 9 | 12 |
| Session/Module | 36 | 35 | 45 |
| Time or duration |  |  |  |
| Program | 8 | 8 | 10 |
| Session/Module | 7 | 7 | 9 |
| Retention/Attrition/Dropout rates | |  |  |
| Treatment | 4 | 4 | 5 |
| Study | 32 | 32 | 41 |
| **Quality of engagement** |  | 42 |  |
| Intensity of specific component usage | 17 | 15 | 36 |
| Completion rates of specific components | 14 | 12 | 29 |
| Time or duration spent in specific component | 18 | 12 | 29 |
| Adherence | 10 | 9 | 21 |
| **Qualitative engagement** |  | 89 |  |
| Satisfaction measures | 62 | 52 | 67 |
| Feedback measures | 42 | 35 | 45 |
| Semi-structured interviews | 11 | 11 | 14 |
| ‘Think-aloud' | 2 | 2 | 3 |
| Focus groups | 3 | 3 | 4 |
| Other | 6 | 5 | 6 |
